# Targeted isolation of photoactive pigments from mushrooms yielded a highly potent new photosensitizer: 7,7′-biphyscion

**DOI:** 10.1038/s41598-022-04975-9

**Published:** 2022-01-21

**Authors:** Fabian Hammerle, Isabella Bingger, Andrea Pannwitz, Alexander Magnutzki, Ronald Gstir, Adriano Rutz, Jean-Luc Wolfender, Ursula Peintner, Bianka Siewert

**Affiliations:** 1 Institute of Pharmacy/Pharmacognosy and Center for Molecular Biosciences Innsbruck (CMBI), University of Innsbruck, Innrain 80-82, 6020 Innsbruck, Austria; 2 Department of Biotechnology, MCI Management Center Innsbruck, 6020 Innsbruck, Austria; 3 Leiden Institute of Chemistry, Gorlaeus Laboratories, Leiden University, P.O Box 9502, 2300 RA Leiden, The Netherlands; 4 Austrian Drug Screening Institute GmbH (ADSI) and Institute of Analytical and Radiochemistry, University of Innsbruck, Innrain 80-82, 6020 Innsbruck, Austria; 5 School of Pharmaceutical Sciences, University of Geneva, CMU-Rue Michel-Servet 1, 1211 Geneva 4, Switzerland; 6 Institute of Pharmaceutical Sciences of Western Switzerland, University of Geneva, CMU-Rue Michel-Servet 1, 1211 Geneva 4, Switzerland; 7 Institute of Microbiology, University of Innsbruck, Technikerstraße 25, 6020 Innsbruck, Austria

**Keywords:** Biophysical chemistry, Natural products, Small molecules, Single-molecule biophysics, Drug discovery, Drug discovery and development, Fungi, Drug discovery and development, Photobiology

## Abstract

Pigments of fungi are a fertile ground of inspiration: they spread across various chemical backbones, absorption ranges, and bioactivities. However, basidiomycetes with strikingly colored fruiting bodies have never been explored as agents for photodynamic therapy (PDT), even though known photoactive compound classes (e.g., anthraquinones or alkaloids) are used as chemotaxonomic markers. In this study, we tested the hypothesis that the dyes of skin-heads (dermocyboid Cortinarii) can produce singlet oxygen under irradiation and thus are natural photosensitizers. Three photosensitizers based on anthraquinone structures were isolated and photopharmaceutical tests were conducted. For one of the three, i.e., (–)-7,7′-biphyscion (**1**), a promising photoyield and photocytotoxicity of EC_50_ = 0.064 µM against cancer cells (A549) was found under blue light irradiation (λ_exc_ = 468 nm, 9.3 J/cm^2^). The results of molecular biological methods, e.g., a viability assay and a cell cycle analysis, demonstrated the harmlessness of **1** in the dark and highlighted the apoptosis-inducing PDT potential under blue light irradiation. These results demonstrate for the first time that pigments of dermocyboid Cortinarii possess a so far undescribed activity, i.e., photoactivity, with significant potential for the field of PDT. The dimeric anthraquinone (–)-7,7′-biphyscion (**1**) was identified as a promising natural photosensitizer.

## Introduction

Mushrooms, the versatile fruiting bodies of fungi, trigger very different feelings in the western world: they scare the inexperienced mushroom hunter but delight the skilled pickers' tongue. While in Europe mushrooms tend to be reduced to their nutritional value, the Asian culture values fungi also as medicinal remedies. For example, *Ganoderma lucidum* is recognized for its anticancer action^1^ and *Hericium erinaceus* for its anti-inflammatory effects^2^. The phytochemical principles behind these bioactivities are triterpenes and sterols, respectively. Another compound class of fungi with pharmaceutical effect is the class of anthraquinones (AQs), responsible for the laxative effect of certain colorful mushrooms^3^, i.e., species belonging to the subgenus dermocyboid Cortinarii.

Looking with a photochemist's eye at the structure of these fungal AQs^4^, however, an additional potential pharmacological application can be suggested: Photodynamic therapy (PDT)^5^. PDT as a treatment strategy utilizes the synergistic effect of the light and the drug, the so-called photosensitizer (PS). In detail, the PS transforms the absorbed light into toxic reactive oxygen species (ROS), inducing cell death at the irradiation site. PDT is a promising alternative to classic chemotherapy to combat resistant cancer types and nonsurgical tumors^6–9^, which is—due to the needed spatial light activation—expected to reduce the painful side-effects of chemotherapeutics. Nevertheless, the treatment-specific adverse reactions of the most commonly used porphyrin-like photosensitizers (i.e., general photosensitivity of the skin) force the search for new scaffolds^10^.

One alternative PS is hypericin, the active ingredient of *Hypericum perforatum*. Intriguingly, the quinoid PS was not only discovered in several plant species of the genus Hypericaceae but also in colorful mushrooms (i.e., dermocyboid Cortinarii)^11^. Though this is known for more than 30 years, further investigations illuminating the potential of dermocyboid Cortinarii in PDT are limited to a pilot study^12^, in which a *Cortinarius croceus* extract was identified to possess a promising and selective photocytotoxicity. Inspired by this, the present study was designed to isolate PDT-active PSs from colorful dermocyboid Cortinarii. Dermocyboid Cortinarii were initially considered a genus of agaricoid Basidiomycota (i.e., *Dermocybe*)^13^ and were classified into four sections based on pigment occurrence (refer Table 1)^14^. For each section, unique AQ patterns were utilized as chemotaxonomic markers^15^. Some of these AQs equal photosensitizers which are proposed as PDT agents, as e.g., emodin, physcion, or hypericin. Thus, we hypothesize that the subgenus contains several unknown and known photoactive constituents, which may act as PDT-like compounds.

**Table 1:** Selected representatives of the dermocyboid Cortinarii sorted according to their pigmentation type and section.

Species	Pigmentation-type	Section
*C. olivaceofuscus*	Cinnamomea	*Holoxanthae*
*C. cinnamomeoluteus*		*Dermocybe*
*C. uliginosus*		
*C. malicorius*	Malicoria	*Malicoriae*
*C. phoeniceus*	Sanguinea	*Sanguineae*
*C. semisanguineus*		
*C. cinnabarinus*	Cinnabarina	

To test this hypothesis, seven species being representatives of the four pigmentation types were selected (Table 1) and submitted to a photo-activity workflow^12^ consisting of an (i) HPLC-DAD-MS fingerprint analysis, (ii) a singlet-oxygen detection assay (i.e., DMA-assay), and (iii) a (photo)cytotoxicity screening. Subsequently, the most active extract was subjected to in-depth mycochemical analysis, including the photoactivity-guided isolation of secondary metabolites.

This study reports the extracts' photo- and bioactivities, the activity-guided isolation, and the photopharmacological evaluation of the most active fungal PSs. All technical results of this multidisciplinary research study are reported and discussed in detail in the electronic supplementary information (ESI). In contrast, the key results are presented in the main manuscript to achieve concise communication.

## Material and methods

Detailed information on analytical instruments, reagents, and general materials used in this study are provided in the electronic supplementary information (refer ESI, Chapters 1.1 and 1.2).

### Fungal biomaterial

Fruiting bodies of the investigated *Cortinarius* species were collected in different European countries in the late twentieth century. *C.* *malicorius* was collected in 2018 in Italy. Sampling permissions were obtained for all voucher materials collected in Italy. All study/experimental protocols involving fungal materials were conducted in accordance with institutional, national, and international guidelines and legislation. The Conventions on Biological Diversity were followed and, where applicable, The Nagoya Protocol on Access to Genetic Resources and the Fair and Equitable Sharing of Benefits Arising from their Utilization to the Convention on Biological Diversity. All exact collection sites and names of the respective collectors are listed in Table 2. Voucher specimens of all species are deposited in the mycological collection of the Tiroler Landesmuseen (IBF, Naturwissenschaftliche Sammlungen|Tiroler Landesmuseen 2021 (tiroler-landesmuseen.at).

**Table 2:** *Cortinarius* collections used in this study with respective voucher numbers and collection data.

	Voucher	leg. et det.	Origin	Year of collection
*C. uliginosus* Berk.	IBF19951120	Pilzverein Jenbach/M. Moser	Austria, Tyrol	1995
*C. phoeniceus* (Bull.) R. Maire (*C. purpureus* (Bull. ex Pers.) Bidaud, Moënne-Locc. & Reumaux)	IBF19750022	M. Moser	Germany, Baden-Württemberg	1975
*C. semisanguineus* (Fr.) Gillet	IBF19740665	M. Moser	Sweden, Småland, Femsjö	1974
*C. cinnabarinus* Fr.	IBF19801005	R. Pöder	Sweden, Småland, Femsjö	1980
*C. olivaceofuscus* Kühner *(D. carpineti* nom. inval.)	IBF19750023	M. Moser	Swiss, Basel	1975
*C. cinnamomeoluteus* P.D. Orton	IBF19710420	M. Moser	Swiss, Luzern	1971
*C. malicorius* Fr.	IBF2018009	D. Borghi	Italy, South Tyrol	2018

### Preparation of extracts

After the collection, the fresh fungal material was air dried and stored in paper bags. The dried fruiting bodies of the seven dermocyboid *Cortinarius* species were ground to a fine powder using a laboratory mill (mesh size = 0.5 mm) and then subjected to Soxhlet extraction. Approximately two grams of each species were weighed into extraction thimbles and defatted using petroleum ether (V = 500 mL, t = 8 h 20 min, T_water bath_ = 76 °C). After the first extraction step, the biomaterials were allowed to dry overnight at room temperature. Then, exhaustive extraction with methanol was conducted (V = 500 mL, t = 7 h 15 min, T_water bath_ = 90 °C). The extracts were evaporated to dryness at T = 40 °C under reduced pressure and stored in a desiccator. All steps were done under the exclusion of light. The yields and the color of the extracts, as well as the starting materials, are given in the supplementary information (refer ESI, Chapter 1.4, Table S1).

For the in-depth study of *C.* *uliginosus*, the extraction was carried out on a larger scale. Ground fruiting bodies (m = 78.84 g) were weighed into a large extraction thimble and subjected to Soxhlet extraction under reduced pressure. First, the powder was defatted with petroleum ether (V = 2500 mL, t = 17 h 35 min, p = 350 mbar, T_water bath_ = 30 °C) and then dried overnight at room temperature. The petroleum ether was removed after decantation using vacuum rotary evaporation at T = 40 °C to yield m = 1.31 g (η = 1.7% d.w.). Subsequently, the same biomaterial was extracted with methanol (V = 1900 mL, t = 19 h 22 min, p = 275–310 mbar, T_water bath_ = 40 °C). The solution was evaporated to dryness at T = 40 °C under reduced pressure to yield m = 18.47 g (η = 23.4% d.w.) of methanol extract.

All extraction procedures were conducted under the exclusion of direct sunlight using aluminum foil. The extracts were stored in a desiccator at room temperature prior to further analysis.

### Liquid–liquid fractionation

An aliquot of the *C.* *uliginosus* methanolic extract (m = 18.4 g) was dissolved in water (V = 500 mL) and transferred into a separating funnel. The solution was partitioned with diethyl ether (V = 350 mL, n = 4), ethyl acetate (V = 300 mL, n = 4), and water-saturated n-butanol (V = 220 mL, n = 3). The individual fractions were dried using vacuum rotary evaporation at T = 40 °C and stored in a desiccator. The supplementary information (refer ESI, Chapter 1.8, Table S3, Fig. S3) provides the yields of the fractions, their colors, and their HPLC profiles.

### Photoactivity-guided isolation of secondary metabolites

The isolation of compounds **1**–**3** from *C.* *uliginosus* is reported in detail in the supplementary information (refer ESI, Chapters 1.9–1.11). Briefly, after liquid–liquid fractionation of the methanolic extract, fractions were further separated and analyzed using standard phytochemical methods (e.g., column chromatography and HPLC). All generated fractions were evaluated on their ability to generate singlet oxygen with the DMA assay^12^. Compound **1** (η = 8.3 mg, 0.04% w/w_MeOH_) was isolated from the diethyl ether fraction employing dry column vacuum chromatography. Compounds **2** (η = 1.8 mg, 0.01% w/w_MeOH_) and **3** (η = 4.5 mg, 0.02% w/w_MeOH_) were obtained from the ethyl acetate fraction using flash chromatography, dry column vacuum chromatography, and preparative HPLC. The spectral and physicochemical data of **1**–**3** are provided in the supplementary information (refer ESI, Chapter 1.12).

### Analytical profiling

The 14 fungal extracts (i.e., seven MeOH and seven PE extracts) were dissolved in DMSO (c = 1 mg/mL) and analyzed by high performance liquid chromatography (HPLC) using an Agilent Technologies 1200 Series system equipped with a Synergi MAX-RP 80 Å column (150 × 4.60 mm, 4 micron) from Phenomenex (Aschaffenburg, Germany) as stationary phase. The mobile phase (A) was water, (B) consisted of acetonitrile and 0.1% formic acid. Elution was performed in gradient mode starting with 10% B to 50% from 0 to 30 min, 50% B to 90% B from 30–34 min, 90% B to 90% B from 34–50 min, 90% B to 10% B from 50–55 min, followed by t = 10 min of re-equilibration with 90% A. The DAD was set to 210, 254, 330, and 468 nm, and flow rate, sample volume and column temperature were adjusted to 0.5 mL/min, V = 5 μL, and T = 35 °C, respectively. The chromatograms observed at λ = 468 nm are depicted in Fig. S1. For HPLC–MS analyses, an Agilent Technologies 1260 Infinity II HPLC system was coupled to an amaZon iontrap mass spectrometer (Bruker, Bremen, Germany) utilizing the same stationary phase and mobile phase as mentioned above. MS spectra were recorded in negative ESI mode, with a drying gas temperature of T = 320 °C, the nebulizer gas (nitrogen) set to 25 psi, and a nebulizer flow (nitrogen) of 12 l/min. The scanned mass range was between m/z 70–1500, at a capillary voltage of 4.5 kV.

### Photochemical and photophysical investigation

The fungal extracts were dissolved in DMSO, mixed with an ethanolic 9,10-dimethylanthracene (DMA)-solution, and irradiated with blue light (λ = 468 nm)^12^. The generated singlet oxygen was then indirectly quantified via the quenching of absorbance at λ = 377 nm. Berberine was used as a reference compound. An accurate protocol for conducting the DMA-assay is provided in the supplementary (ESI chapter 1.6). The photophysical investigation of the isolated compounds **1**–**3**, i.e., the calculation of the singlet oxygen quantum yields, the recording of luminescence spectra, and the determination of photostability, was carried out as described in the supplementary (refer ESI, Chapter 3).

### Molecular networking

The methanolic extract of *C.* *phoeniceus* as well as the methanolic extract of *C.* *uliginosus,* including its four fractions resulting from the liquid–liquid extraction (i.e., diethyl ether, ethyl acetate, n-butanol, and water), were subjected to a feature-based molecular networking analysis. Briefly, the extracts and fractions were analyzed by UHPLC-DAD-MS/MS. Acquired MS data were first converted from .RAW standard format to .mzXML format and then treated using the MZmine software suite v. 2.38^16^. After mass detection, chromatogram building, deconvolution, adduct search, dereplication using an in-house database of fungal pigments, alignment, and gap-filling, a feature-based molecular network was created with the online workflow on the GNPS website. The visualization of the network was done with the software Cytoscape^17^. Metabolite annotation was performed combining in-house library dereplication, in-silico annotation with Sirius^18^, and a script for taxonomically informed metabolite annotation. The detailed workflow of the molecular networking analysis is given in the supplementary material (refer ESI, Chapter 2).

### Stability testing of isolated secondary metabolites

A stability evaluation of the isolated compounds **1**–**3** was conducted to investigate the influence of different temperature and light conditions. Briefly, **1** was dissolved in dimethylformamide (DMF) and aliquots were pipetted into white-glass HPLC-vials. The vials were stored under five different light/temperature conditions, and the residual concentration of **1** (relative to the original concentration) was determined after a whole week (refer ESI, Chapter 4). In addition, the stability of **1**–**3** under cell culture conditions was investigated. The DMSO stock solutions of **1**–**3** were stored in brown-glass HPLC-vials at room temperature and under the exclusion of light. Over a period of 33 h and 19 min, the solutions were repeatedly measured by HPLC, and the remaining concentrations of **1**–**3** were calculated relative to their original concentration (ESI chapter 4.2). Serial dilutions of the DMSO stocks of **1**–**3** in Opti-MEM^®^ were also subjected to stability testing. The dilutions were analyzed by HPLC before and after a period of 24 h at 37 °C (refer ESI, Chapter 4.2). The influence of blue light on the stability of **1** was assessed as well. Dilutions of a DMSO stock of **1** in phosphate buffered saline (PBS), DMEM, and Opti-MEM^®^ were analyzed by HPLC before and after blue light (λ = 468 nm) irradiation (refer ESI, Chapter 4.3).

### (Photo)cytotoxicity assay and cell culture maintenance

Cells of the non-small cell lung cancer cell line A549 (ATCC, Sigma-Aldrich), the cervical cancer cell line HeLa (kindly donated by A. Trockenbacher (Management Center Innsbruck, Austria)), the stomach cancer cell line AGS (CLS, Eppelheim), and the human urinary bladder carcinoma cell line (CLS, Eppelheim) were maintained in T-flasks (25 cm^2^) and MEM-medium containing FCS (10%) and penicillin/streptomycin (1%). Cells were trypsinized every other day and used for approximately 4 weeks. Cell freezing and de-freezing was done according to standard procedures. The (photo)cytotoxicity assay was done as previously published^19^. Briefly, cells (AGS: 2500 cells/well, T24 & A549 & HeLa: 2000 cells/well) were seeded in Opti-MEM^®^ (2.5% FCS, P/S) and after 24 h treated with each extract (50, 25, and 5 µg/mL, stock solution 10 mg/mL in DMSO, max DMSO 0.65%). After additional 24 h, the medium was aspirated and replaced by fresh Opti-MEM^®^. Thereafter, the plate was irradiated for the indicated amount of time (i.e. 0, 2.5, 5, or 7.5 min; 0, 3.1, 6.2, or 9.3 J/cm^2^ respectively). The cells were fixed with chilled trichloroacetic acid (100 µL) after 72 h in total. After washing (4 times 200 µL, water) the wells were stained with sulforhodamine B (SRB, 0.4% in acetic acid (1%)) for 30 min. Then, the plates were rewashed (4 times 200 µL, acetic acid 1%) and dried under air. The dried dye was dissolved with a TRIS solution (10 mM in water, 100 µL) and the absorbance measured at 540 nm with a plate reader (Tecan, Spark, M10). The EC_50_ of berberine and the pure compounds **1**–**3** was calculated with Prism 5.0 employing the relative Hill-Slope equation and is given with its confidence interval (95%). For the extracts, no cytotoxicity was defined if more than 50% of the cell population was existent at 50 µg/mL. Moderate activity was assigned for an extract resulting at 50% cell population in a range of 25–50 µg/mL, high activity between 5 and 25 µg/mL, and exorbitant under 5 µg/mL, respectively. The selectivity indices (S.I.) express the ratio of cells killed in the dark and cells killed under irradiation. It is calculated from the EC_50_ values as the quotient of EC_50|Dark_ and EC_50|Irradiated_.

### Anthraquinone uptake studies

The cellular uptake of the isolated anthraquinones **1**–**3** was investigated via combining in vitro cell culture experiments and liquid-chromatographic analyses (i.e., HPLC–DAD). The exact protocols are given in the supplementary material (refer ESI, Chapter 5.4).

### Metabolic activity assay, cell cycle analysis, and cell viability assay

The metabolic activity of A549 cancer cells treated with **1** was investigated using the resazurin assay (refer ESI, Chapter 5.5). A cell cycle analysis was performed with A549 cancer cells treated with **1** employing flow cytometric analysis (refer ESI, Chapter 5.6). The viability of A549 cancer cells treated with **1** was tested utilizing Annexin V and DRAQ7 (refer ESI, Chapter 5.7).

### Photocytotoxicity inhibition studies

Various antioxidants were assessed for their ability to chemically quench singlet oxygen (DMA studies/refer ESI, Chapter 5.8.1) and to inhibit photodamage induced by **1** on A549 and T24 cancer cells (refer ESI, Chapter 5.8.2).

## Results and discussion

### Photobiological screening and photoactivity guided isolation

**Figure 1:**
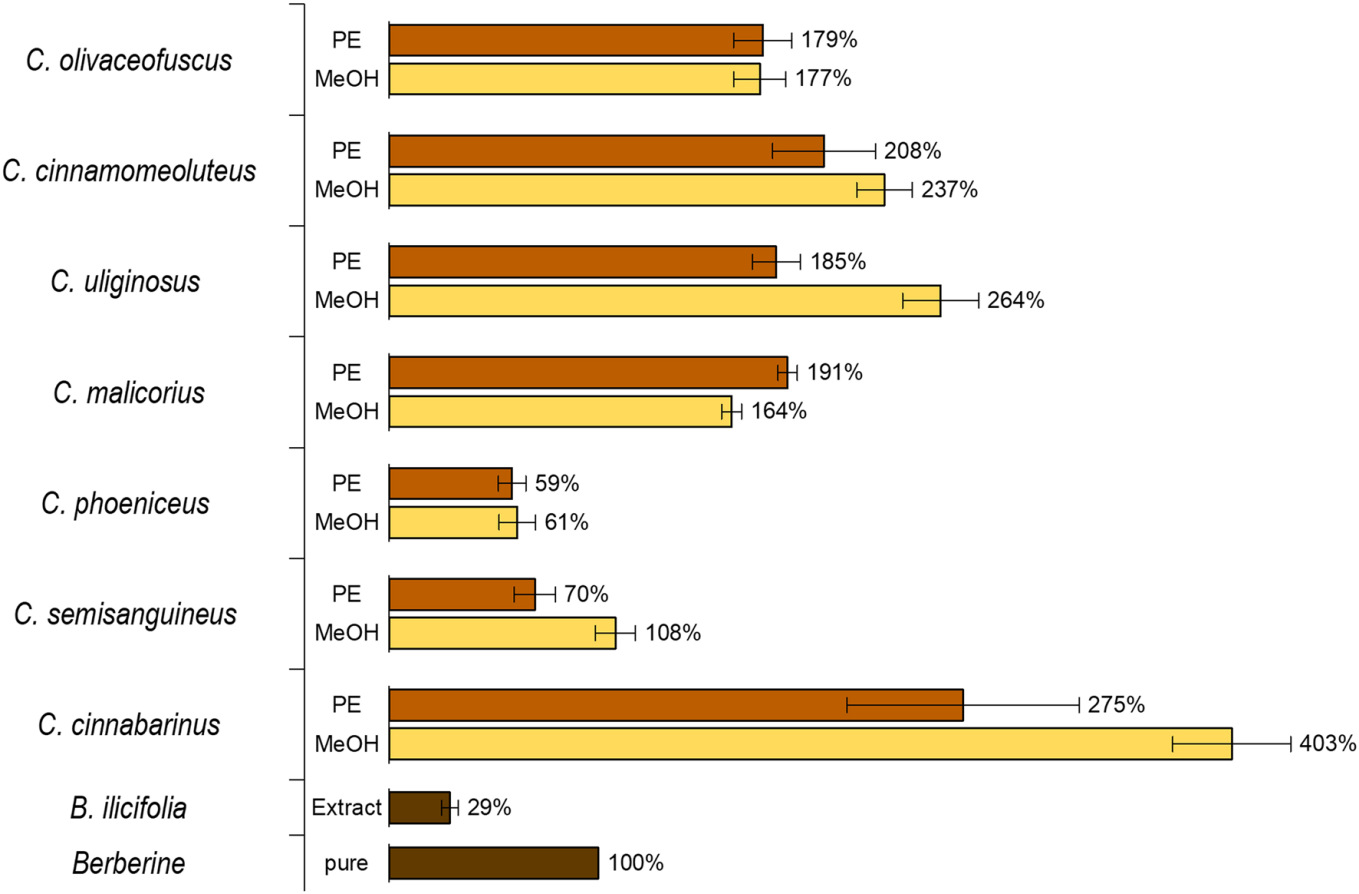
Relative singlet oxygen formation efficacy of fungal extracts (PE = petroleum ether extract, MeOH = methanolic extract, 1 mg/mL) under blue light irradiation (468 nm, 24.3 J/cm^2^). All investigated fungal species were more prone to produce singlet oxygen than an extract containing berberine (λ_max, EtOH_ = 429 nm, ϕ_EtOH_ = 0.05), i.e., a *Berberis ilicifolia* root extract. The latter is a plant species commonly known as barberry, which contains the photosensitizer berberine in its roots.

The HPLC–DAD-MS analysis of the fungal extracts revealed that the apolar fractions were characterized at most with two major pigments (mono- and/or bis-anthraquinones). In comparison, the polar extracts consisted of up to seven pigments (refer ESI, Fig. S1). These results corresponded with the previous work utilizing complete extracts and thin-layer chromatographic methods^15^. An UV–Vis analysis disclosed that most pigments were characterized by an absorbance maximum between 450 and 500 nm (refer ESI, Table S2). As a consequence, the DMA-assay was done employing blue light (λ_irr_ = 468 nm, H = 24.3 J/cm^2^) and the natural photosensitizer berberine (λ_max, EtOH_ = 429 nm, ϕ_Δ,EtOH_ = 0.05^20^). Additionally, a methanolic extract of the roots of *Berberis ilicifolia* was used to rank the fungal extract's potential compared to an herbal extract. As shown in Fig. 1, all extracts were more active than the *B.* *ilicifolia* extract, and most extracts were more active than the standard berberine (Fig. 1). Thus,—for the first time—a general photoactivity of fruiting bodies was revealed in European species of dermocyboid Cortinarii.

The activity profile of the mushrooms could be ranked according to their pigment profile (Table 1). In detail, mushrooms belonging to the sanguinea pigment type were less active than those belonging to the malicoria and cinnamomea pigment type. Fruiting bodies of the cinnabarina pigmentation type were the most active and thus was *C. cinnabarinus* (403%). The cinnabarina pigmentation type is atypical for European species and more common for species of the Southern Hemisphere^15,21^.

The results of the photo-cytotoxicity assay (refer ESI, Table S10) showed, however, that a high singlet-oxygen production rate alone is a poor indicator for a selective photo-cytotoxic activity. The PE extract of *C.* *cinnabarinus*, for example, was an efficient producer of ^1^O_2_ (403%), but its dark cytotoxicity was too pronounced (EC_50_ = 28 µg/mL, A549 cells) and consequently the by light induced selectivity factor of three not prominent enough (Table S10, EC_50, BL, A549_ = 9 µg/mL *vs* EC_50, Dark, A549_ = 28 µg/mL). Moreover, the MeOH extract of *C. cinnabarinus* (^1^O_2_ = 275%) lacked activity under irradiation, indicating cellular uptake deficits. In analogy, the other extracts of the fruiting bodies belonging to Moser's section sanguinea (i.e., *C.* *phoeniceus* and *C.* *semisanguineus*) lacked either photo-activity in vitro (MeOH extracts) or held insignificant selectivity indices (PE extracts, SI_DarK/BL_ = max. 4). The PE extracts of the three investigated species of the cinnamomea pigmentation type (i.e., *C. olivaceofuscus*, *C.* *cinnamomeoluteus*, and *C.* *uliginosus*) showed a similar pattern. The results of the respective methanolic fractions, however, were of utmost interest: All extracts of the cinnamomea pigmentation type were highly active (EC_50_ = 2–7 µg/mL) under irradiation (468 nm, 9.3 J/cm^2^) while being inactive in the dark (EC_50_ > 50 µg/mL). For the isolation, the most promising dermocyboid Cortinarius, i.e., *C.* *uliginosus*, was chosen.

**Figure 2:**
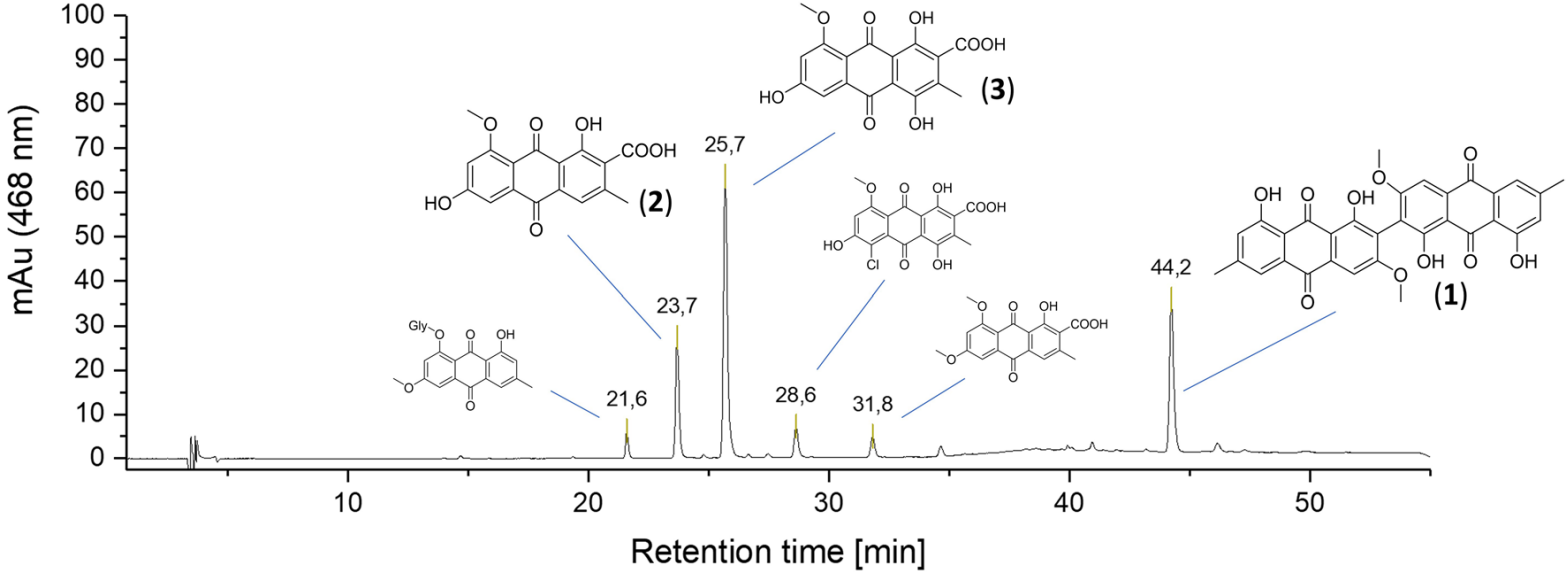
HPLC chromatogram (λ_det_ = 468 nm) of the EtOAc fraction of *C.* *uliginosus* obtained by a liquid–liquid fractionation from the methanol extract. The detection wavelength was fitted to the excitation wavelength of the accompanied assays (i.e., (photo)cytotoxicity and DMA assay). Peaks of interest are indicated by their retention time and by the identified structure. The small structures indicate solely annotated structures, while the normal-sized ones are such which were isolated and are thus structurally verified. Stationary phase: Max RP, Mobile Phase (H_2_O/ACN + 0.1%FA), gradient (for details refer SI, Chapter 1.5).

Starting with dried and ground fruiting bodies (m = 80.0 g), a sequential Soxhlet-extraction with PE and MeOH yielded a red (1.7 wt %, 1.3 g) and an orange (23.5 wt %, 18.5 g) viscose fluid. The MeOH fraction was separated further into four fractions via liquid–liquid extraction. The highest photoactivities were found in the diethyl ether (316%) and ethyl acetate (251%) fractions (Fig. S3). While the diethyl ether fraction consisted of only one prominent peak (t_r_ = 44.2 min) absorbing at 468 nm (Fig. S1), the ethyl acetate fraction showed (Fig. 2) two additional significant (t_r_ = 23.7 and 25.7 min) and three minor pigments (t_r_ = 21.6, 28.6, and 31.8 min).

Feature-based molecular networking (FBMN)^22^ analysis was conducted to support the annotation of *C.* *uliginosus*' secondary metabolites. FBMN, a state-of-the-art metabolomics tool, is used to visualize complex data obtained through untargeted LC–MS/MS metabolite profiling analyses. Furthermore, it is employed to identify extract constituents based on structural similarity. Hence, the two methanolic extracts (i.e., *C.* *uliginosus* and *C.* *phoeniceus*) and four fractions (i.e., diethyl ether, ethyl acetate, n-butanol, and water) were subjected to UHPLC-HRMS/MS analysis. The acquired data were pretreated and a molecular network was generated using the GNPS platform^22^. The principles of bioactive natural product prioritization^23^ were used to organize and explore the network to spot unknown metabolites of interest. Evidently (refer ESI, Chapter 2 for the detailed results of the FBMN study), the bioactivity can be attributed to AQs in the apolar fractions. Based on all gathered insights, the compound appearing at 44.2 min was annotated as 7,7′-biphyscion (**1**) and isolated via dry column vacuum chromatography (refer ESI, Chapters 1.7–1.10 for the detailed discussion of the isolation process) as an orange powder (8.32 mg, 0.04% d.w.). The annotation as 7,7′-biphyscion (**1**) was confirmed via standard techniques (NMR, IR, UV–Vis, HRMS, m.p., refer ESI Chapter 1.12.1 for the complete structure discussion). The specific rotation of **1** was determined to be levorotary ([α]_D, T = 25 °C_ = − 178 (c = 0.1 mg/mL)).

Via photoactivity-guided fractionation of the ethyl acetate fraction and semi-preparative HPLC, the peaks at t_r_ = 23.6 min and t_r_ = 25.7 min were additionally isolated (for details refer ESI, Chapter 1.11). These metabolites could be identified as the known fungal AQs^24^ dermolutein (**2**) and dermorubin (**3**), respectively (refer ESI, Chapters 1.12.2 and 1.12.3 for the complete structural assignment). The minor peaks were putatively annotated as physcion-8-*O*-glycoside (t_r_ = 21.6 min), 5-chloro-dermorubin (t_r_ = 28.6 min), and endocrocin-6,8-dimethylether (t_r_ = 31.8 min) based on a comparison of the literature data^15^ with the detected MS signals (refer ESI, Chapter 2).

### Photochemical and photobiological evaluation of the isolated pigments

The three isolated pigments **1**–**3** were submitted to photochemical and -physical studies (refer ESI Chapter 3). As shown in Table 3, all isolated fungal metabolites produced singlet oxygen under irradiation (ACN, 450 nm, 50 mW, d_4_-MeOH). With a quantum yield of 20%, **1** is the most efficient PS of the isolated metabolites of *C.* *uliginosus*. The monomeric AQs **2** and **3** held a singlet oxygen quantum yield (ϕ_Δ_) of 3% and 8%, respectively. Thus, compared to the other known natural PSs based on a dimeric AQ structure, i.e., 5,5′-bisoranjidiol ϕ_Δ_ = 18%^25^, **1** showed similar photophysical properties. The photophysical activity of the monomeric AQs **2** and **3** blended into the wide range of quantum yields described for AQs^26^. Furthermore, luminescence spectra (refer ESI Fig. S30) were recorded, lifetime measurements were done, and emission quantum yields were determined (Table 3, refer ESI Fig. S29). In terms of luminescence, the monomeric AQs were more efficient than the bisanthraquinone **1**, with **3** being the most efficient. The obtained lifetimes in the nanosecond scale indicated the fluorescent nature of the luminescence, which is in line with reports on AQs^27^. Additionally, we were interested in the photostability of the isolated metabolites. A decomposition was observed for the monomeric AQs in methanol under blue light irradiation (450 nm LED, refer ESI Chapter 3.4). The evolution of several new species was detected for **1**. These transformations appeared with a quantum yield of 0.78% in the first 75 min. Thus, only a marginal part of the natural PS **1** decomposes under the given light conditions, and a photo-activated biological effect is highly likely.

**Table 3:** Photochemical properties of **1**–**3** in methanol.

	1	2	3	MeOH extract
λ_abs_^[a]^ [nm] (log ε)	440 (3.48)	427 (3.89)	490 (3.92)	420
		440 (3.85)	530 (3.58)	
λ_ems_^[b]^ [nm]	607	625	593	593
ϕ_F_^[b,c]^	0.016 ± 0.005	0.048 ± 0.005	0.087 ± 0.002	0.04 ± 0.01
τ^[d]^ [ns]	0.8	1.6	4.3	1.1/4.7
ϕ_Δ_^[b,c]^	20 ± 2%	3 ± 0.3%	8 ± 2%	10 ± 1%
ϕ_decom_	0.78%	1.6%	0.18%	–

[a] In MeOH [b] In air-saturated D_4_MeOH [c] Relative measurement using [Ru(bpy)_3_]Cl_2_ as standard with Φ_P_ = 0.015. Laser settings: 450 nm, 15 mW [d] With a resolution of 0.3 ns.

**Figure 3:**
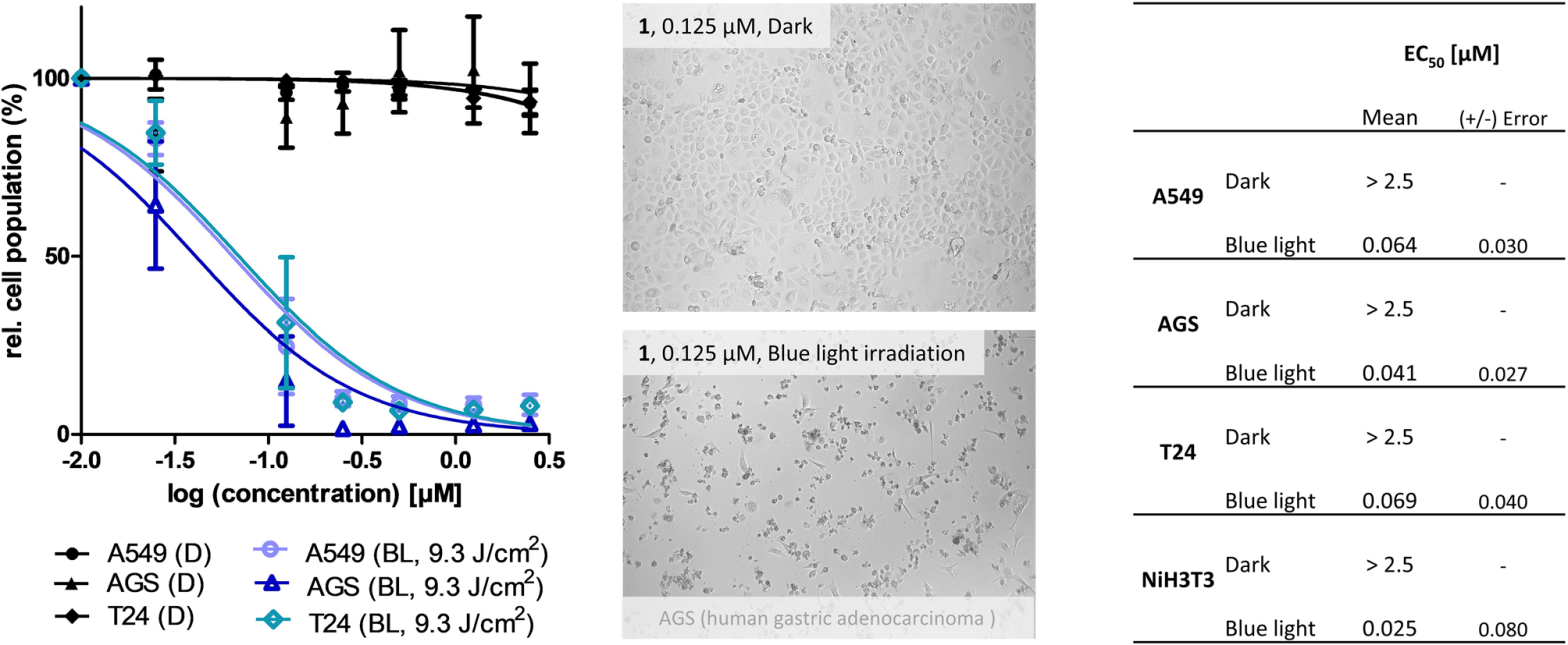
(**Left**) Dose–response curves of the three cell lines treated with **1** under blue light irradiation (λ_exc_ = 468 nm blueish plots) and dark conditions (black plots). Error = standard deviation. (**Middle**) Micrographs (brightfield, 10× objective) of treated (and irradiated) cells. (**Right**) Results of the (photo)cytotoxicity assay given as effective concentrations (EC_50_) in µM with confidence interval (95%).

The photo-activated effect of **1**–**3** was tested via a (photo)cytotoxicity assay employing cells of three malignant cell lines (A549/human lung carcinoma, AGS/human gastric adenocarcinoma, T24/human bladder carcinoma). Figure 3 summarizes the results. A conducted stability assay (ESI Chapter 4) in the medium (refer ESI, Tables S8, S9, Fig. S31) confirmed the stability of the isolated metabolites **1**–**3** beforehand.

The monomeric AQs **2** and **3,** as displayed in Figs. S46–48, were neither in the dark nor under irradiation toxic (EC_50_ > 12.5 µM). A missing cellular uptake might explain the lack of photoactivity. Indeed, a HPLC–DAD-based uptake study showed that **2** and **3** are not intracellularly present (refer ESI, Chapter 5.4 and Fig. S33A). The bisanthraquinone **1**, however, was taken up by the cells (refer ESI, Figs. S33A and S34) and exhibited a significant cytotoxic effect in a nanomolar range (Fig. 3) after being irradiated with blue light (468 nm, 9.3 J/cm^2^) while **1** lacked activity in the dark (EC_50_ > 2.5 µM). The natural PS **1** was also phototoxic for cells of the non-malignant cell line NIH3T3 (Fig. 3). Such lack of selectivity is a severe problem for classic chemotherapeutics. For PSs, however, it is less problematic as the spatial irradiation of the tumorous tissues induces the toxic effect selectively. Under dark conditions, **1** was non-toxic for the fibroblasts.

**Figure 4:**
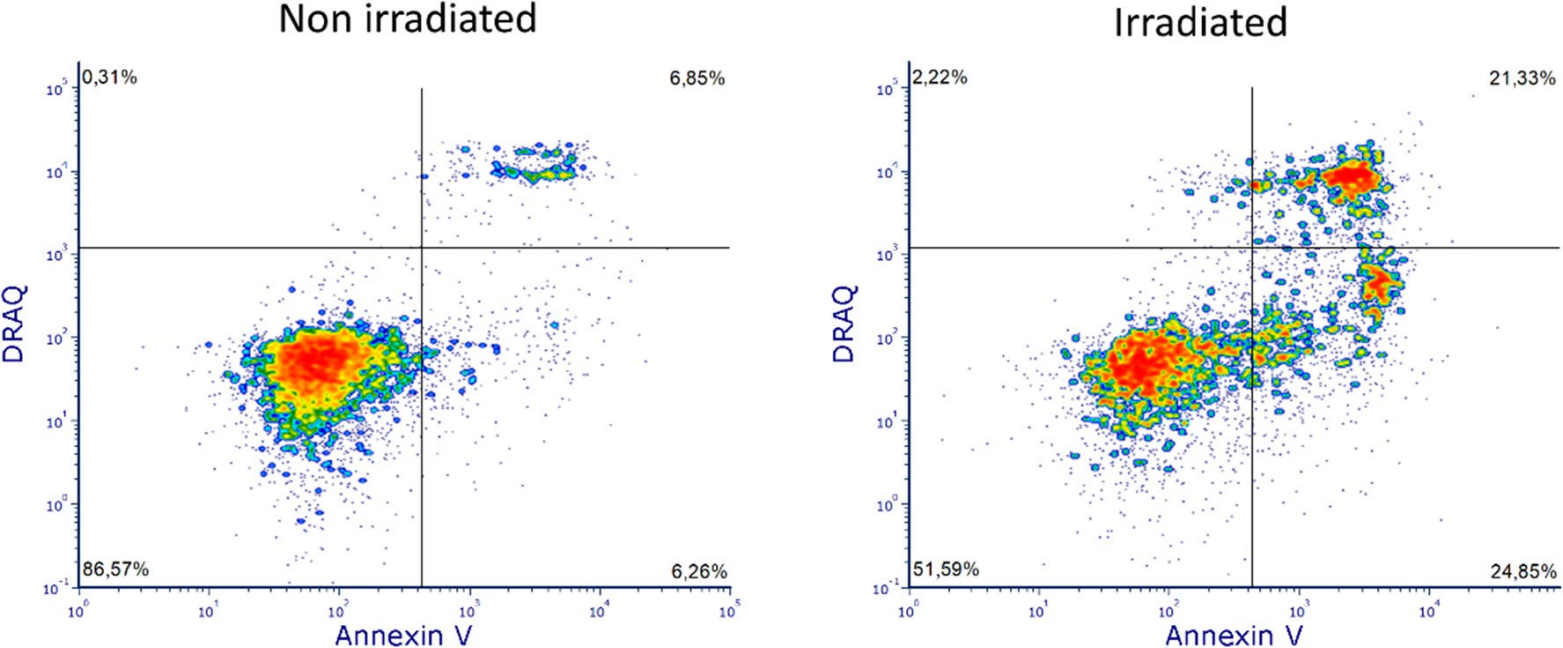
Annexin V/DRAQ7 assay 24 h after (mock) irradiation of A549 cells treated with **1** (c = 0.5 µM). Left) non-irradiated cells showing no sign of induced apoptosis and right) irradiated cell population containing a clear apoptotic cell population.

To verify the harmlessness of **1** in the dark, we conducted (i) a metabolic activity assay, which results proved that **1**—without irradiation—does not interfere with the metabolic activity of A549 cells (refer to ESI, Fig. S36). Furthermore, we conducted (ii) a cell cycle analysis. The results (refer to ESI, Fig. S37) showed no differences between control and treatment, portraying **1** as innocuous in the dark. And finally, (iii) a viability assay (annexin V/DRAQ7) also confirmed the harmlessness of **1** in the dark (Figs. 4 and S38–S41).

Under irradiation, a different pattern was observed for cells being treated with **1**: 24 h after the external trigger, 45% percent of all cells were apoptotic (22% early apoptotic and 23% secondary apoptotic) (Fig. 4). At higher concentrations of **1** (i.e., c = 1 µM), more apoptotic cells were observed (i.e., 79% refer ESI, Figs. S40 and S41). These results are in line with the noticed morphological changes. Cells treated with **1** and blue light were shrunken, membranes blebbed, and nuclei condensed (Figs. S47–S49). In contrast, cells treated under dark conditions did not show any morphological changes compared to the non-irradiated control.

To understand the effect of light irradiation on the bioactivity of **1** in more detail, we performed several analyses: (i) Irradiating **1** in OMEM medium (w/ FCS) showed that 6.1% of **1** were degraded after 7.5 min (9.3 J/cm^2^, 468 nm) (Fig. S32). In contrast, in solutions without FCS, significantly more degradation was observed (Fig. S31, up to 65% were degraded after irradiation). As a consequence, an involvement of ROS in the degradation mechanism is implied since FCS is a known ROS scavenger^28^. However, a single photochemical degradation product of **1** was not detected in any of the HPLC–DAD analyses (Fig. S35). To (ii) test whether a solution of **1** could be used in several irradiation cycles, cells were treated with pre-irradiated solutions of **1** and (mock)-irradiated after 24 h of incubation again. After this subsequent irradiation, a similar EC_50_ value (51 ± 14 nM) was calculated compared to the standard protocol. This result proved the opportunity of a pulsed irradiation cycle. Also, under dark conditions, the pre-irradiation of **1** did not change the activity profile (i.e., pre-irradiated **1** is non-toxic too (EC_50, Dark_ > 2.5 µM)), which proved the harmlessness of the produced degradation products.

Photocytotoxicity inhibition studies were conducted with different antioxidants (refer ESI, Chapter 5.4) to understand the induced ROS better. Under cell-free conditions, 75% of the ROS production was quenched by sodium azide (Table S11). This reduction proved singlet oxygen as major ROS, as sodium azide is a physical quencher of singlet oxygen^29^. Sodium azide is, however, not recommended for in vitro assays due to its dual function as singlet oxygen quencher^29^ and potent inhibitor of the cytochrome oxidase of the electron transport system^30^. Thus, a ROS-dependent mode-of-action was demonstrated by a co-treatment with N-acetyl-l-cysteine and β-carotene. In cells of the A549 and T24 cell lines, the in vitro photodamage was reduced by up to 52% (refer ESI chapter 5.4.2).

With these results, **1** is one of the most potent AQ-based PS so far known: With its average EC_50, BL_ of approx. 0.05 µM **1** exceeds the photocytotoxicity of the reported monomeric AQs rubiadin (EC_50, MCF7_ = 74 µM) and soranjidiol (EC_50, MCF7_ = 37 µM)^31^ by more than 1300 fold. Thus, **1** represents a natural compound with a true potential as PDT hit structure. Furthermore, **1** demonstrated in vitro activities not inferior to those of transition metal complexes already used in clinical trials (i.e., Ru(II) polypyridiyl complex TLD1433 with an EC_50_ of 51 nM against cells of the U87 human glioblastoma cell line, activated by green light (530 nm, 45 J/cm^2^))^32,33^. Finally, the apoptosis-inducing character of **1** under irradiation rendered this new natural photosensitizer highly promising.

## Conclusion

In this study, the first PSs (**1**–**3**) were isolated from fungal fruiting bodies in a targeted manner. Their structures belong to the photoactive class of anthraquinones^26^. In the plant kingdom (Plantae), photoactive AQs are part of the plant defense mechanisms^34–37^. This fact hints towards a potentially undiscovered ecological function of the fungal AQs.

The ability of 7,7′-biphyscion (**1**) to produce (i) singlet oxygen (ϕ_Δ_ = 20%), (ii) its stability, and (iii) its photopharmacological potential with a selectivity index of over 39, (iv) an EC_50_ in the lower nanomolar range (i.e., 64 nM, A549), and (v) its ability to induce apoptosis turn this first fungal PS of basidiomycetes into a promising new candidate for PDT. The fact that **1** was also discovered in endophytic fungi^38^ bears the potential of biotechnical production.

## Supplementary Information

Supplementary Information.

## Abbreviations

ACN: Acetonitrile
AQ: Anthraquinone
BL: Blue light
DAD: Diode array detector
DMA: 9,10-Dimethylanthracene
DRAQ: Dark red anthraquinone 7
EC_50_: Half maximal effective concentration
ESI: Electronic supplementary information
FBMN: Feature-based molecular network
FCS: Fetal calve serum
HPLC: High performance liquid chromatography
HRMS/MS: High-resolution tandem mass spectrometry
IR: Infrared
LED: Light emitting diode
m.p.: Melting point
MeOH: Methanol
MS: Mass spectrometry
NMR: Nuclear magnetic resonance
^1^O_2_: Singlet oxygen
OMEM: Opti-MEM
PDT: Photodynamic therapy
PE: Petrol ether
PS: Photosensitizer
ROS: Reactive oxygen species
TLC: Thin-layer-chromatography
SI: Selectivity indices
UHLPC: Ultra-high performance liquid chromatography
UV–Vis: Ultraviolet–visible
