# Supplementary material for: Targeted isolation of photoactive pigments from mushrooms yielded a highly potent new photosensitizer: 7,7′-biphyscion
Source: Sci Rep. 2022 Jan 21;12:1108. doi: 10.1038/s41598-022-04975-9 (PMC8782903; doi:10.1038/s41598-022-04975-9)
Supplement: Supplementary file 1 — Supplementary Information. [file 41598_2022_4975_MOESM1_ESM.docx]

Supporting Information

# **Targeted Isolation of Photoactive Pigments from Mushrooms Yielded a Highly Potent New Photosensitizer: 7,7’-Biphyscion**

Fabian Hammerle^a^, Isabella Bingger^b^, Andrea Pannwitz^c^, Alexander Magnutzki^d^, Ronald Gstir^d^, Adriano Rutz^e^, Jean-Luc Wolfender^e^, Ursula Peintner^f^, and Bianka Siewert^*a^

# Abstract

Pigments of fungi are a fertile ground of inspiration: they spread across various chemical backbones, absorption ranges, and bioactivities. However, basidiomycetes with strikingly colored fruiting bodies have never been explored as agents for photodynamic therapy (PDT), even though known photoactive compound classes (e.g., anthraquinones or alkaloids) are used as chemotaxonomic markers.

In this study, we tested the hypothesis that the dyes of skin-heads (dermocyboid Cortinarii) can produce singlet oxygen under irradiation and thus are natural photosensitizers. Three photosensitizers based on anthraquinone structures were isolated and photopharmaceutical tests were conducted. For one of the three, i.e., (–)-7,7’-biphyscion (**1**), a promising photoyield and an excellent photocytotoxicity of EC_50_ = 0.064 µM against cancer cells (A549) was found under blue light irradiation (λ_exc_ = 468 nm, 9.3 J/cm²). The results of molecular biological methods, e.g., a viability assay and a cell cycle analysis, demonstrated the harmlessness of **1** in the dark and highlighted the apoptosis-inducing PDT potential under blue light irradiation. These results demonstrate for the first time that pigments of dermocyboid Cortinarii possess a so far undescribed activity, i.e., photoactivity, with significant potential for the field of PDT. The dimeric anthraquinone (–)-7,7´-biphyscion (**1**) was identified as promising natural photosensitizer.

**Table of contents**

[1. Extraction and Activity-Guided Isolation of the Fungal Material 4](#_Toc90301694)

[1.1. Instruments 4](#_Toc90301695)

[1.2. Reagents and materials 4](#_Toc90301696)

[1.3. Extraction of the selected Cortinarii species 5](#_Toc90301697)

[1.4. Analytical profiling 5](#_Toc90301698)

[1.5. The DMA-assay 8](#_Toc90301699)

[1.6. Soxhlet extraction of the *Cortinarius uliginosus* biomaterial 8](#_Toc90301700)

[1.7. Liquid-liquid extraction of the methanolic extract 9](#_Toc90301701)

[1.8. Bioactivity-guided isolation of 1 10](#_Toc90301702)

[1.8.1. Dry column vacuum chromatography of DFH1A_Et2O 10](#_Toc90301703)

[1.8.2. Isolation of 1 from DFH1E_2 11](#_Toc90301704)

[1.9. Exhaustive isolation of (-)-7,7´-biphyscion (1) 11](#_Toc90301705)

[1.10. Bioactivity-guided isolation of dermolutein (2) and dermorubin (3) 12](#_Toc90301706)

[1.10.1. Flash chromatography of DFH1A_EtOAc 12](#_Toc90301707)

[1.10.2. Dry column vacuum chromatography of DFH1B_3 12](#_Toc90301708)

[1.10.3. Preparative HPLC of DFH1B1_2 and DFH1B1_3 13](#_Toc90301709)

[1.11. Structure characterization of the isolated compounds 14](#_Toc90301710)

[1.11.1. (-)-7,7’-Biphyscion (1) [CAS Registry Number: 39772-01-5] 14](#_Toc90301711)

[1.11.2. Dermorubin (3) [CAS Registry Number: 26071-14-7] 18](#_Toc90301712)

[1.11.3. Dermolutein (2) [CAS Registry Number: 26071-13-6] 20](#_Toc90301713)

[2. Phytochemical Profiling and Molecular Networking 23](#_Toc90301714)

[2.1. MS Data Pretreatment 23](#_Toc90301715)

[2.2. Molecular Networks Generation 23](#_Toc90301716)

[2.3. In-house Library 23](#_Toc90301717)

[2.4. Annotation by Sirius 23](#_Toc90301718)

[2.5. Taxonomically Informed Metabolite Annotation Molecular Networks Generation (ISDB-DNP-Taxo) 24](#_Toc90301719)

[2.6. Generation of the Variable “Vis Signal” 24](#_Toc90301720)

[2.7. Untargeted Molecular Network Investigation 24](#_Toc90301721)

[2.8. Targeted annotation of the relevant pigments 30](#_Toc90301722)

[3. Photophysical and Photochemical Investigation 32](#_Toc90301723)

[3.1. Experimental Setup 32](#_Toc90301724)

[3.2. Near infrared spectra 32](#_Toc90301725)

[3.3. Emission spectra 33](#_Toc90301726)

[3.4. Photostability assay – Instrumental setup 33](#_Toc90301727)

[4. Stability testing of **1** 34](#_Toc90301728)

[4.1. Stability testing under cell culture conditions 35](#_Toc90301729)

[4.2. The influence of time and temperature 35](#_Toc90301730)

[4.3. The influence of blue light irradiation on the stability of **1** 36](#_Toc90301731)

[*4.3.1.* The influence of blue light irradiation on the stability of **1** dissolved in OPTI-MEM® (cell culture conditions) 37](#_Toc90301732)

[5. Biological testing 39](#_Toc90301733)

[5.1. (Photo)cytotoxicity-Assay and Cell Culture Maintenance 39](#_Toc90301734)

[5.2. (Photo)toxicity results of the extracts 39](#_Toc90301735)

[5.3. (Photo)toxicity results of **1-3** 41](#_Toc90301736)

[5.4. Uptake studies 41](#_Toc90301737)

[5.4.1. Uptake of 1 42](#_Toc90301738)

[5.4.2. Anthraquinone uptake studies: conclusions 43](#_Toc90301739)

[5.5. Metabolic Activity assay 44](#_Toc90301740)

[5.6. Cell Cycle Analysis 44](#_Toc90301741)

[5.7. Cell viability assay 45](#_Toc90301742)

[5.8. Photocytotoxicity Inhibition Studies 47](#_Toc90301743)

[5.8.1. Singlet oxygen quenching (DMA studies) 47](#_Toc90301744)

[5.8.2. Inhibition of (-)-7,7´-biphyscion (1) photodamage 48](#_Toc90301745)

[5.8.3. Photocytotoxicity studies: conclusions 49](#_Toc90301746)

[5.9. Micrographs of treated cells. 50](#_Toc90301747)

[References 52](#_Toc90301748)

1. Extraction and Activity-Guided Isolation of the Fungal Material
   1. Instruments

The fungal biomaterial was ground with the mill IKA Labortechnik MF10 basic (IKA®-Werke GmbH & Co. KG, Staufen, Germany), which was equipped with a 0.5 mm mesh. For weighing in of samples the weighing instruments KERN ALS 220-4 (KERN & SOHN GmbH, Balingen-Frommern, Germany) and Sartorius Cubis®-series (Sartorius AG, Göttingen, Germany) were used. The evaporation of solvents under reduced pressure was done with the rotary evaporators Heidolph LABOROTA 4000-efficient, Heidolph Hei-VAP Precision (Heidolph Instruments GmbH & CO. KG, Schwabach, Germany), and IKA RV 10. All rotary evaporators were equipped with vacuum pumps and vacuum controllers. Additionally, the ultrasonic bathes Sonorex RK 106, Sonorex RK 52, and Sonorex TK 52 (BANDELIN electronic GmbH & Co. KG, Berlin, Germany) were utilized. Pictures of thin-layer chromatographic analyses were taken with the Camag Reprostar 3 and edited with the software winCATS (CAMAG Chemie-Erzeugnisse & Adsorptionstechnik AG, Muttenz, Switzerland). For flash-chromatography BÜCHI´s Reveleris® X2-system (BÜCHI Labortechnik AG, Flawil, Switzerland) was used. HPLC measurements were carried out using the modular system Agilent Technologies 1260 Infinity II with a quaternary pump, vial sampler, column thermostat, diode-array detector, and mass spectrometer. Moreover, the HPLC-system Agilent Technologies 1200 Series with binary pump, autosampler, column thermostat, and diode-array detector was used. Both mentioned HPLC-systems were purchased from Agilent Technologies, Inc. (Santa Clara, USA). For preparative HPLC chromatography, a Dionex (Thermo Fisher Scientific Inc., Waltham, USA) HPLC-system, consisting of a Dionex UltiMate 3000 pump, an ASi-100 Automated Sample Injector, a Dionex UltiMate 3000 Column Compartment, a Dionex UVD170U, and a Gilson 206 Fraction collector in combination with a Synergi MAX-RP 80 Å column (250 x 10.00 mm, 4 micron) from Phenomenex (Aschaffenburg, Germany) was used. As part of the DMA-assay an illumination setup, consisting of the power adaptor Agilent E3611A DC Power Supply and a 468 nm LED-panel (University Leiden, published in Hopkins et al.^1^), was made use of. Absorption measurements were done with the Shimadzu UV-1800 spectrophotometer (Shimadzu Europa GmbH, Duisburg, Germany) or with the plate reader Tecan Spark® 10M (Tecan Group Ltd., Männedorf, Switzerland). Furthermore, the vortex mixer Vortex-Genie 2 (Scientific Industries, Inc., Bohemia, New York) was used and the adjustment of pH-values was carried out with the pH-meter Mettler Toledo SevenMulti (Mettler-Toledo GmbH, Vienna, Austria). Pipetting was done with tips from Eppendorf AG (Hamburg, Germany) and STARLAB International GmbH (Hamburg, Germany). The pipettes were purchased from the aforementioned companies too. Reagent reservoirs were obtained from Thermo Fischer Scientific (Waltham, Massachusetts, USA). The filtration of organic solutions was performed with Rotilabo®-syringe filters (PFTE) with a mesh size of 0.45 µm (Carl Roth GmbH + Co. KG, Karlsruhe, Germany). ^1^H and ^13^C NMR spectra were acquired using two spectrometers from Bruker, an Avance II 600 spectrometer operating at 600 MHz (^1^H) and 151 MHz (^13^C) and an Avance III HD spectrometer operating at 400 MHz (^1^H) (Bruker Corporation, Billerica, USA). The spectra were recorded in deuterated solvents supplied by Euriso-Top (Cambridge Isotope Laboratories, Inc., Saint-Aubin, France). Optical rotation data was acquired on a JASCO P-2000 polarimeter (Jasco Deutschland GmbH, Pfungstadt, Germany). IR spectra were recorded on an ALPHA FT-IR apparatus (Bruker, Ettlingen, Germany) equipped with a Platinum ATR module. All other specific instruments are listed in the respective chapters.

- 1. Reagents and materials

All solvents for the extraction and isolation processes were purchased from VWR International (Vienna, Austria). Ethyl acetate and acetone were distilled prior to their use. Solvents for HPLC experiments had pro analysis (p.a.) quality at least and were obtained from Merck (Merck KGaA, Darmstadt, Germany). The Sartorius arium® 611 UV purification system (Sartorius AG, Göttingen, Germany) was used to obtain ultrapure water. Silica gel 40–63 μm and pre‐packed cartridges for flash chromatography were purchased from Merck (Darmstadt, Germany) and Büchi (BÜCHI Labortechnik AG, Flawil, Switzerland), respectively. The reagents emodin (product-number: E0500) and 9,10-dimethylanthracene (product-number: D0252) were sourced from TCI Deutschland GmbH. Thin-layer chromatographic analysis was performed using silica TLC plates 60 F254 with a layer thickness of 0.2 mm from Merck.

- 1. Extraction of the selected Cortinarii species

The yields as well as the color of the extracts can be found in Table S1.

**Table S1.** Overview of extraction-yields gained.

|  | Mass [g] | Solvent | Yield [mg, dw] | Colour |
| --- | --- | --- | --- | --- |
| *C. uliginosus* | 2.13 | petroleum ether | 51 (2.4 %) | yellow-green |
|  |  | methanol | 797 (37.4 %) | red |
| *C. phoeniceus* | 1.98 | petroleum ether | 50 (2.5 %) | light orange |
|  |  | methanol | 884 (44.8 %) | dark red |
| *C. semisanguineus* | 2.08 | petroleum ether | 33 (1.6 %) | light orange |
|  |  | methanol | 750 (36.0 %) | red |
| *C. cinnabarinus* | 2.08 | petroleum ether | 37 (1.8 %) | green |
|  |  | methanol | 775 (37.2 %) | orange |
| *C. olivaceofuscus* | 2.06 | petroleum ether | 15 (0.7 %) | light green |
|  |  | methanol | 687 (33.3 %) | dark orange |
| *C. cinnamomeoluteus* | 3.62 | petroleum ether | 18 (0.5 %) | yellow |
|  |  | methanol | 885 (24.4 %) | brown |
| *C. malicorius* | 2.08 | petroleum ether | 22 (1.1 %) | yellow |
|  |  | methanol | 666 (31.9 %) | dark red |

- 1. Analytical profiling

The chromatograms (observed at λ = 468 nm) obtained through the HPLC analysis of the twelve fungal extracts are depicted in Figure S1.


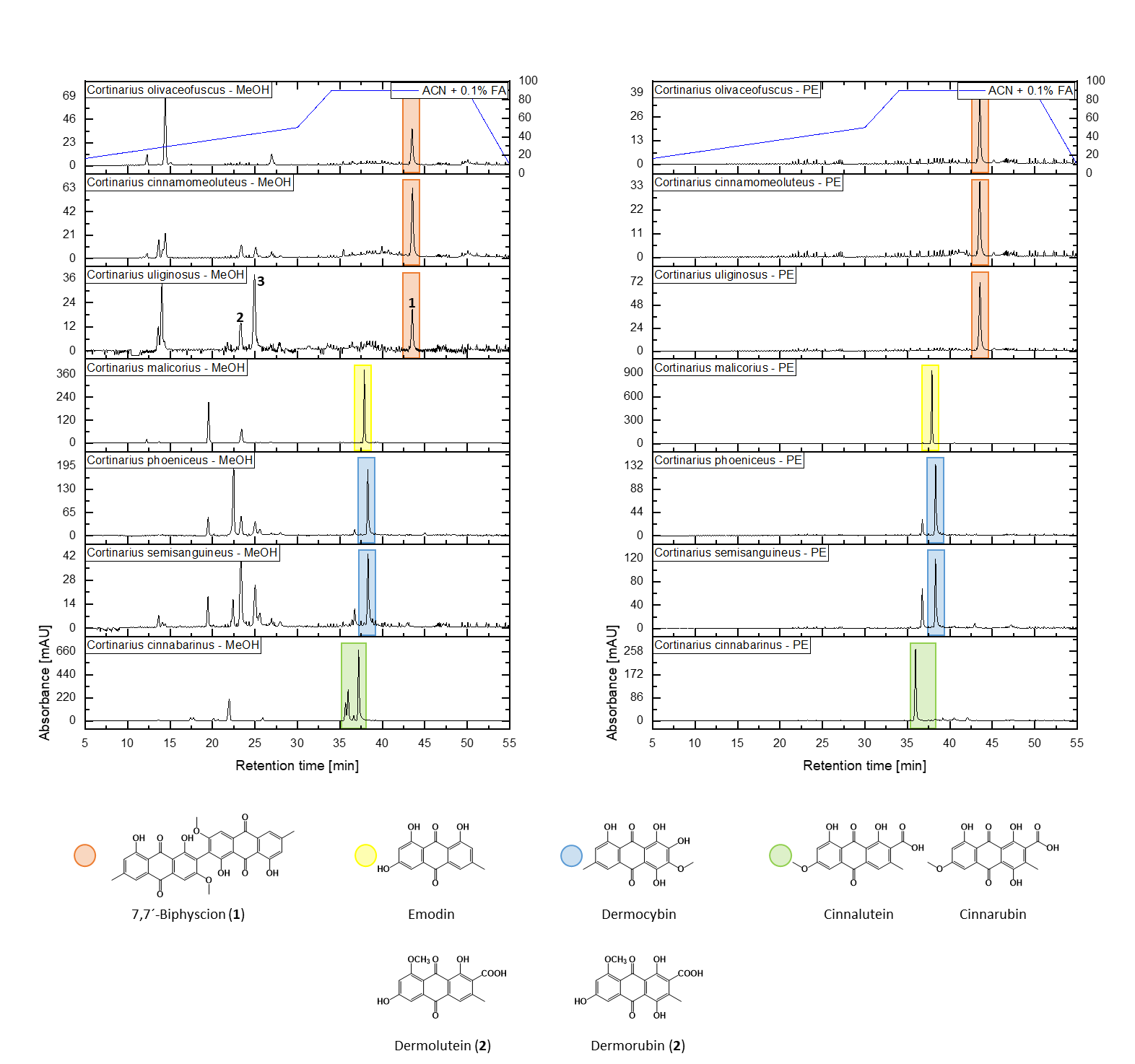


**Figure S1**. Pigment profiling of the investigated Cortinarii. The PE extracts are shown on the left, the methanolic extracts on the right. The lower part of the figure depicts the chemical structures of the putatively annotated compounds. All extracts were dissolved in DMSO (1 mg/mL) for analysis. Stationary Phase: Phenomenex Synergi MAX-RP, Mobile Phase: H_2_O/ACN (+0.1 % FA), ratio of solvent B (% ACN) is displayed in the highest chromatogram by the blue graph. Chromatograms are recorded at λ = 468 nm. In each chromatogram the group specific pigment is highlighted by colored boxes: orange = 7,7´-biphyscion (**1**), yellow = emodin, blue = dermocybin, green = cinnalutein and cinnarubin.

Referring to the four pigmentation types of Cortinarii (cinnamomea, sanguinea, cinnabarina, malicoria) described by Keller^2^, the assignment of the investigated fungi to those groups was possible due to the analytical data gained by HPLC-DAD-MS. *Cortinarius uliginosus*, *C. olivaceofuscus,* and *C. cinnamomeoluteus* formed group 1 (cinnamomea-pigmentation type), which was characterized through a common peak with a retention time of 44.2 minutes ((-)-7,7´-biphyscion (**1**), Figure S1 orange box). Group 2 (sanguinea-pigmentation type), consisting of *C. phoeniceus* and *C. semisanguineus*, showed the same peaks at the retention times 23.4 minutes (dermolutein (**2**)), 25.0 minutes (dermorubin (**3**)), and 22.5 minutes as well as a characteristic peak at a retention time of 38.3 minutes (dermocybin, Figure S1 blue box). The pigment patterns of *C. cinnabarinus*, the sole member of group 3 (cinnabarina-pigmentation type) displaying peaks with retention times of 36.0 (cinnalutein) and 37.6 minutes (cinnarubin, Figure S1 green box), as well as *C. malicorius*, belonging to the malicoria-pigmentation type (group 4) and showing a peak at a retention time of 37.88 minutes (emodin, Figure S1 yellow box), were distinct from all of the other fungi's patterns and therefore assigned to separate groups.

Table S2 lists all major peaks of each fungus with retention times and absorption max.

**Table S2.** All major peaks of each fungal extract with retention times and absorption maxima.

|  | Extract | Retention time [min] | λmax [nm] |
| --- | --- | --- | --- |
| *Cortinarius olivaceofuscus* | MeOH | 12.30 | 438 |
|  |  | 14.45 | 438 |
|  |  | 27.00 | 442 |
|  |  | 43.52 | 442 |
|  | PE | 43.52 | 442 |
| *Cortinarius cinnamomeoluteus* | MeOH | 13.67 | 430 |
|  |  | 14.45 | 438 |
|  |  | 23.41 | 440 |
|  |  | 25.10 | 486 |
|  |  | 43.55 | 440 |
|  | PE | 43.55 | 440 |
| *Cortinarius uliginosus* | MeOH | 13.61 | 428 |
|  |  | 14.04 | 486 |
|  |  | 23.35 | 438 |
|  |  | 24.95 | 482 |
|  |  | 43.53 | 440 |
|  | PE | 43.53 | 440 |
| *Cortinarius malicorius* | MeOH | 12.26 | 416 |
|  |  | 13.72 | 430 |
|  |  | 19.55 | 426 |
|  |  | 23.44 | 438 |
|  |  | 37.88 | 438 |
|  | PE | 37.88 | 438 |
| *Cortinarius phoeniceus* | MeOH | 19.50 | 416 |
|  |  | 22.49 | 484 |
|  |  | 23.39 | 440 |
|  |  | 25.03 | 484 |
|  |  | 25.60 | 442 |
|  |  | 36.74 | 428 |
|  |  | 38.33 | 484 |
|  | PE | 36.75 | 430 |
|  |  | 38.33 | 484 |
| *Cortinarius semisanguineus* | MeOH | 13.66 | 432 |
|  |  | 19.48 | 418 |
|  |  | 22.43 | 480 |
|  |  | 23.39 | 440 |
|  |  | 25.03 | 484 |
|  |  | 25.60 | 442 |
|  |  | 36.74 | 428 |
|  |  | 38.33 | 484 |
|  | PE | 36.74 | 428 |
|  |  | 38.33 | 484 |
| *Cortinarius cinnabarinus* | MeOH | 17.43 | 422 |
|  |  | 17.77 | 488 |
|  |  | 20.15 | 424 |
|  |  | 20.67 | 424 |
|  |  | 21.97 | 478 |
|  |  | 25.93 | 438 |
|  |  | 35.68 | 438 |
|  |  | 35.98 | 436 |
|  |  | 36.64 | 438 |
|  |  | 37.20 | 490 |
|  | PE | 35.98 | 436 |
|  |  | 42.09 | 490 |

- 1. The DMA-assay

The DMA-assay represents a low-cost, medium-throughput possibility to spot photoactive compounds, which can be easily implemented in the activity-guided isolation process of novel photosensitizers.^3^ 9,10-Dimethylanthracene (DMA) is a fluorescent polycyclic aromatic hydrocarbon that reacts selectively with singlet oxygen (^1^O_2_) yielding the non-fluorescent 9,10-endoperoxide (Figure S2) in numerous organic solvents as well as in water.^4^ This reactivity poses the basis of the DMA-assay, where 9,10-dimethylanthracene is used as chemical probe to detect ^1^O_2_ produced by an extract, a fraction, or a pure compound. It is possible to indirectly quantify the singlet oxygen yield by observing the decrease of fluorescence at a wavelength of 436 nm as well as the missing optical density > 350 nm of the 9,10-endoperoxide if an adequate positive control with known ^1^O_2_-production is present.^3^


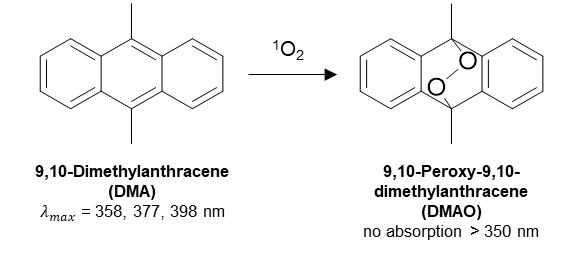


**Figure S2.** Chemical quenching of the DMA absorbance due to singlet oxygen.

**Assay protocol**: Two stock solutions, an ethanolic DMA-solution (1.4 mM) and a L-ascorbic acid solution (100 mM, pH = 7.0-7.4) are prepared. Of these and of pure ethanol, four working solutions (i.e., pure ethanol, DMA-solution (0.3325 mM), L-ascorbic-acid solution (47.5 mM), and a mix of DMA- and L-ascorbic acid-solution at the identical concentration) are generated. Each working solution (190 µL) is pipetted into a 96-well plate. Then the sample solution (1 mg/mL, DMSO, 10 µL) is added. DMSO (10 µL) is used as blank and berberine (1 mg/mL, 2.97 mM, DMSO, 10 µL) as positive control derived from a natural source. Thereafter, the optical density at the wavelengths 377 and 468 nm is measured with a plate reader, followed by four cycles of irradiating the plate with blue light ($\lambda$ = 468 nm, 6.2 J/cm²) for five minutes and subsequent measuring of the absorption at the described wavelengths. Hence a total irradiation time of 20 minutes (468 nm, 24.8 J/cm^2^) was applied. All measurements were done as technical duplicate. The singlet oxygen production was calculated relative to berberine with formula (1).^3^

^1^O_2_ [%] = $\frac{\Delta\mathrm{OD}\left( DMA;Extract \right)-\Delta OD (DMA;Solvent CTR)}{\Delta\mathrm{OD}\left( DMA;Berb \right)-\Delta OD(DMA;Solvent CTR)}\times\frac{1-{10}^{{-OD}_{Berb;468 nm}}}{1-{10}^{{-OD}_{Extract;468 nm}}}\times100$ (1)

- 1. Soxhlet extraction of the *Cortinarius uliginosus* biomaterial

The dried fruiting bodies of *C. uliginosus* were milled to a fine powder (mesh size 0.5 mm) and stored in a paper bag under exclusion of light and moisture. Then the ground biomaterial (m = 78.84 g) was extracted via Soxhlet extraction under reduced pressure. At first, the powder was defatted with petroleum ether (V = 2500 mL) for 17 h 35 min (p = 350 mbar, T_water bath_ = 30 °C). After the defatting process the biomaterial was dried at room temperature. The solution was evaporated to dryness at 40 °C under reduced pressure to yield 1.31 g (1.7 % dw). Subsequent to the first extraction steps, the same biomaterial was extracted with methanol (V = 1900 mL for 19 h 22 min (p = 275-310 mbar, T_water bath_ = 40 °C). The methanolic solution was evaporated to dryness at 40 °C under reduced pressure to yield 18.47 g (23.4 % dw).

- 1. Liquid-liquid extraction of the methanolic extract

The fraction yields, their colors and the results of their photochemical investigation are depicted in the table below (Table S3).

**Table S3.** Yield, colour, and singlet oxygen production [%] of the respective fraction.

| Fraction | Yield [g] | Colour | Results DMA-assay [%] |
| --- | --- | --- | --- |
| diethyl ether | 1.84 (9.9 %) | burnt umber | 316 |
| ethyl acetate | 0.63 (3.4 %) | burnt umber | 251 |
| n-butanol | 14.36 (77.7 %) | burnt umber | 70 |
| water | 1.08 (5.8 %) | dark red | 8 |

The HPLC chromatograms of the methanolic extract and the fractions resulting from the liquid-liquid extraction as well as the results of the DMA-assay are summarized in Figure S3*.*


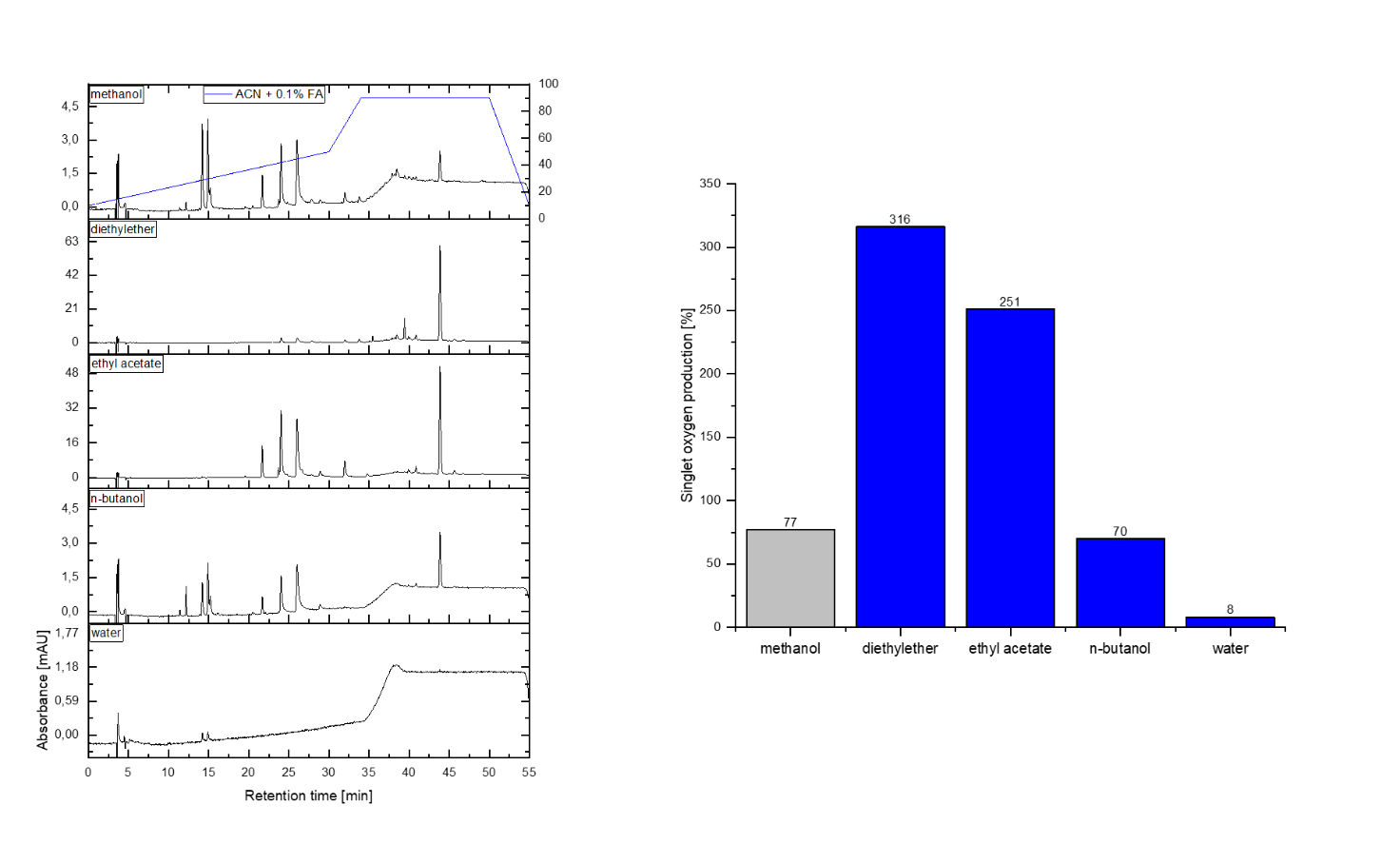


**Figure S3.** The chromatograms of the methanolic extract (labelled "methanol") and the fractions resulting from the liquid-liquid extraction (labelled "diethylether", "ethyl acetate", "n-butanol", "water") recorded at $\lambda$ = 450 nm are shown on the left side. All fractions were solved in DMSO (c = 1 mg/ml) for analysis. Stationary phase: Phenomenex Synergi MAX-RP, Mobile phase: H_2_O/ACN (+ 0.1 % FA). On the right side, the results of the DMA-assay (Singlet oxygen production (%)) of the extract and the fractions are depicted.

- 1. Bioactivity-guided isolation of 1

All conducted isolation and chemical profiling experiments regarding **1** are discussed separately in the following chapters.

- - 1. Dry column vacuum chromatography of DFH1A_Et2O

An aliquot of the diethyl ether fraction (m = 1.46 g), which was obtained through liquid-liquid separation of the methanolic extract (chapter 1.7), was subjected to dry column vacuum chromatography. Isocratic elution was performed under reduced pressure with silica gel 60 (0.040-0.063 mm) purchased from Merck (Darmstadt, Germany) as stationary phase. The solvent mixture Tol:MeOH:EtOAc:FA = 94:2.5:2.5:1 was used as mobile phase. After the isocratic elution, the column was washed with methanol. In total, 53 fractions with a volume of approximately 5 ml each were obtained and subsequently analyzed via thin-layer chromatography (SiO_2_, Tol:MeOH:EtOAc:FA = 94:2.5:2.5:1). Based on a similar elution pattern, the 53 fractions were merged into five. The solutions were evaporated to dryness at 40 °C under pressure and kept in a desiccator. Based on the results of the HPLC analysis (Figure S4) and the DMA-assay (Figure S4) done with these five fractions, fraction DFH1E_2 (m = 167 mg, 11.4 %) was subjected to further isolation steps.


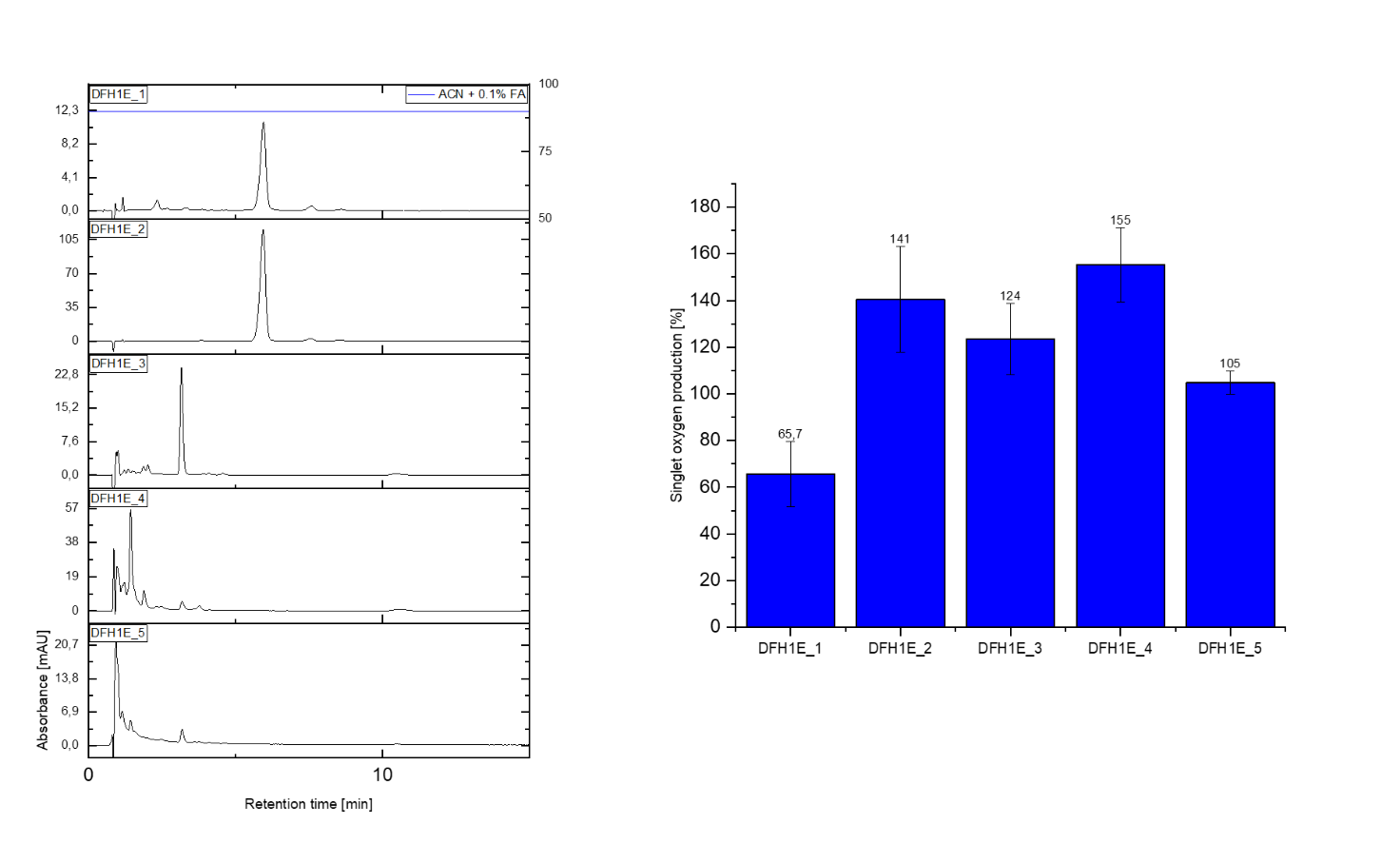


**Figure S4.** On the left side, the chromatograms of the fractions DFH1E_1-5 recorded at $\lambda$ = 468 nm are shown. All fractions were dissolved in DMSO (c = 1 mg/mL) for analysis. Stationary phase: Phenomenex Synergi MAX-RP, Mobile phase: H_2_O/ACN (+ 0.1 % FA). On the right side, the results of the DMA-assay (Singlet oxygen production (%) relative to berberine) of the fractions DFH1E_1-5 are depicted.

- - 1. Isolation of 1 from DFH1E_2

DFH1E_2 (32.3 mg) was washed as powder several times with PE and 1,2-dimethoxyethane. The purity was controlled via thin-layer chromatography (SiO_2_, Tol:MeOH:EtOAc:FA = 94:2.5:2.5:1, derivatised with vanillin-sulfuric acid-reagent) and if necessary washed again, finally yielding the fraction DFH1H_1 (m = 10 mg). An aliquot of DFH1H_1 (m = 5.96 mg) recrystallized from EtOAc (77 °C, 2.5 h) yielding analytical pure DFH1I_1 (m = 1.9 mg). All isolation and purification steps are summarized in the flowchart below (Figure S5).


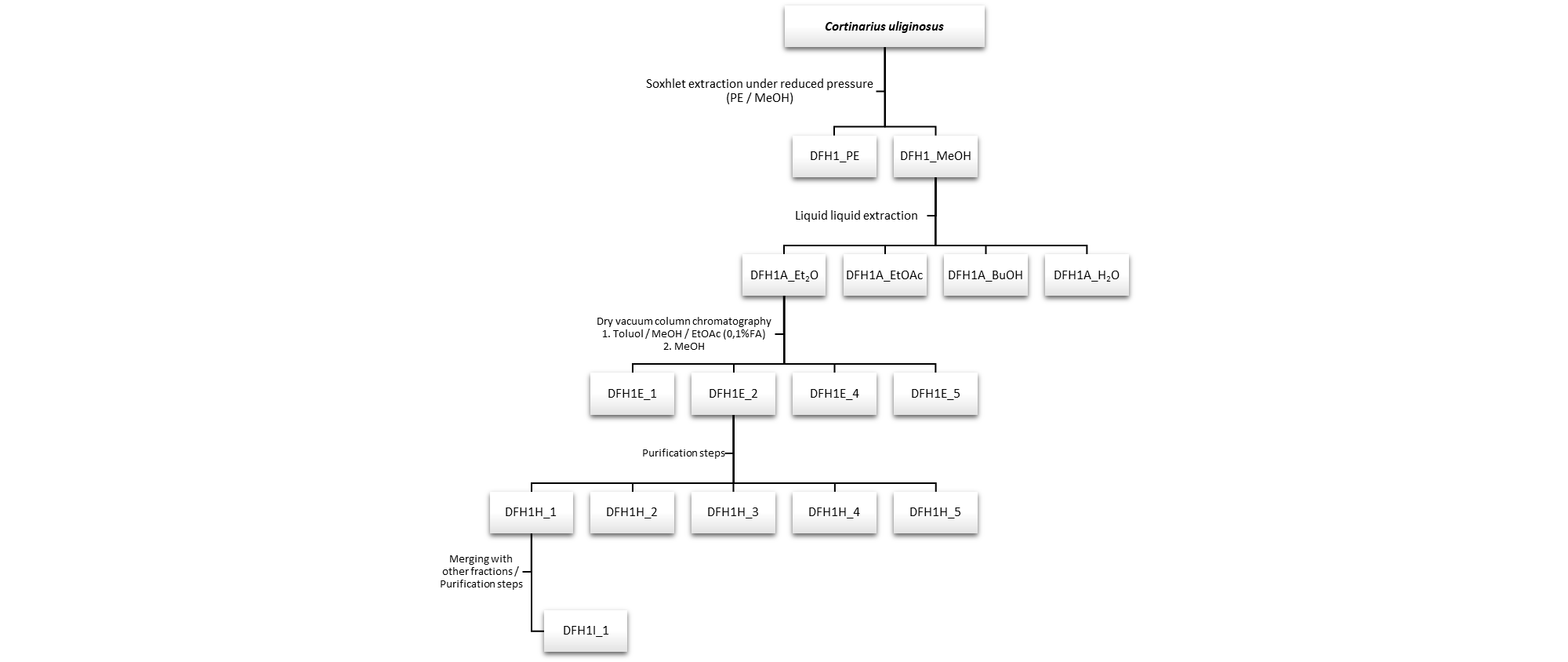


**Figure S5.** Flowchart of the isolation of (-)-7,7´-biphyscion (**1**) yielding 1.9 mg (starting material 78.6 g).

- 1. Exhaustive isolation of (-)-7,7´-biphyscion (1)

The targeted isolation of (-)-7,7´-biphyscion (**1**) from different fractions will be described in this chapter. The fractions were chosen according to their containing pigments (HPLC- and TLC-analysis) and their visual appearance (orange color).

The fractions DFH1H_1 (m = 1.3 mg), DFH1H_2 (m = 9.3 mg), DFH1G_2 (m = 53.5 mg), and DFH1G_3 (m = 12 mg) were merged and subjected to dry column vacuum chromatography. Elution was performed under reduced pressure with silica gel 60 (0.040-0.063 mm) purchased from Merck as stationary phase. First, the sample was washed with petroleum ether (V = 500 ml). After the washing step, isocratic elution with Tol:EtOAc:FA = 10:5:3 was performed. 21 fractions were obtained and analyzed via thin-layer chromatography (mobile phase as described above). The fractions 10-14 were merged and the resulting fraction DFH1K was evaporated to dryness at 40 °C under reduced pressure. Fraction DFH1K was subjected to dry vacuum column chromatography (stationary phase and conduction as described above). Here, the sample was washed with petroleum ether (V = 200 ml) followed by an isocratic elution with Tol:EtOAc:MeOH:FA = 94:2.5:2.5:1. 17 fractions were obtained and analyzed via thin-layer chromatography with the same mobile phase as described beforehand. Fractions 5-10 were merged and the resulting fraction DFH1K was evaporated to dryness at 40 °C under reduced pressure to yield 8.32 mg (0.04 %) of (-)-7,7´-biphyscion (**1**).

- 1. Bioactivity-guided isolation of dermolutein (2) and dermorubin (3)

All conducted isolation and chemical profiling experiments regarding dermolutein (**2**) and dermorubin (**3**) are discussed separately in the following chapters.

- - 1. Flash chromatography of DFH1A_EtOAc

An aliquot (m = 494 mg) of the ethyl acetate fraction (chapter 1.7) was subjected to flash chromatography. The separation was done using a Reveleris® X2-system in combination with a Reveleris® HP Silica 20 µm cartridge. The mobile phases were toluene (A) and methanol (B). Elution was performed in gradient mode with 0 % B to 100 % B from 0 to 45 min and with the flow rate set to 30 mL/min. By this way, 82 fractions with a volume of approximately 20 mL each were obtained, analysed via TLC (SiO_2_, Tol:EtOAc:MeOH = 70:15:15, derivatised with methanolic potassium hydroxide solution), and subsequently merged into 5 fractions (DFH1B1-5). Then the fractions were evaporated to dryness at 40 °C, a small part resolved and submitted to HPLC-DAD analysis and the DMA-assay (Figure S6). Based on the results, fraction DFH1B_3 (164 mg, 33.17 %) was subjected to further isolation steps.


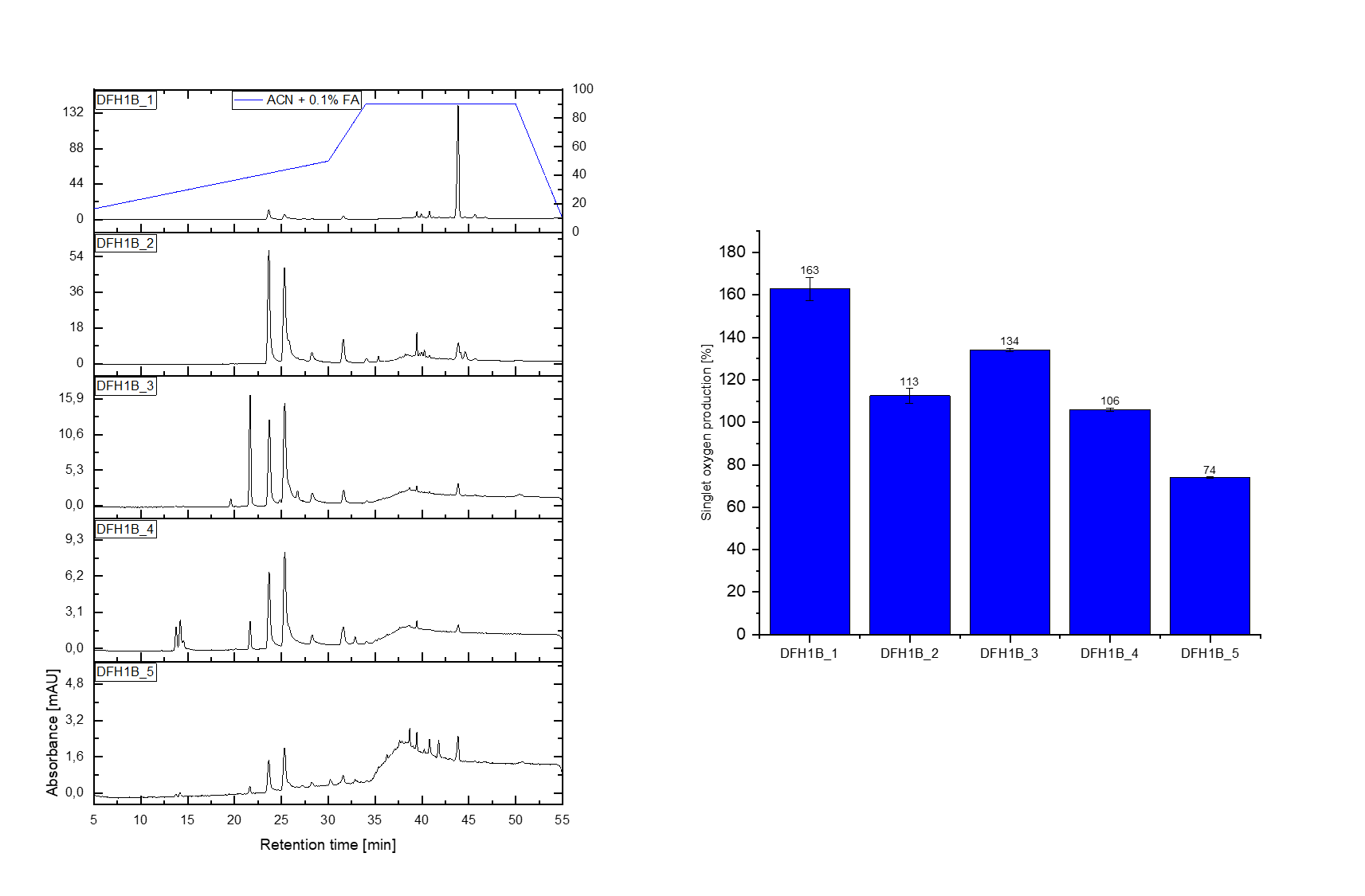


**Figure S6.** On the left side, the chromatograms of the fractions DFH1B_1-5 recorded at $\lambda$ = 468 nm are shown. All fractions were dissolved in DMSO (c = 1 mg/mL) for analysis. Stationary phase: Phenomenex Synergi MAX-RP, Mobile phase: H_2_O/ACN (+ 0.1 % FA). On the right side, the results of the DMA-assay (Singlet oxygen production (%)) of the fractions DFH1B_1-5 are depicted.

- - 1. Dry column vacuum chromatography of DFH1B_3

The fraction DFH1B_3 (m = 164 mg) was subjected to dry column vacuum chromatography. Isocratic elution was performed under reduced pressure with silica gel 60 (0.040-0.063 mm) purchased from Merck as stationary phase. As mobile phase the solvent mixture Tol:MeOH:EtOAc = 7:2.5:2.5 (+ 0.1 % FA) was used. 25 fractions with a volume of approximately 10 mL each were obtained and analyzed via thin-layer chromatography (TLC-system: SiO_2_, Tol:MeOH:EtOAc = 7:2.5:2.5 (+ 0.1 % FA)) and subsequently merged into four fractions (DFH1B1_1-DFH1B1_4). The fractions were analyzed via HPLC-DAD and the DMA-assay (Figure S7). In consequence, DFH1B1_2 (5.08 mg) and DFH1B1_3 (15.44 mg) were subjected to preparative HPLC.


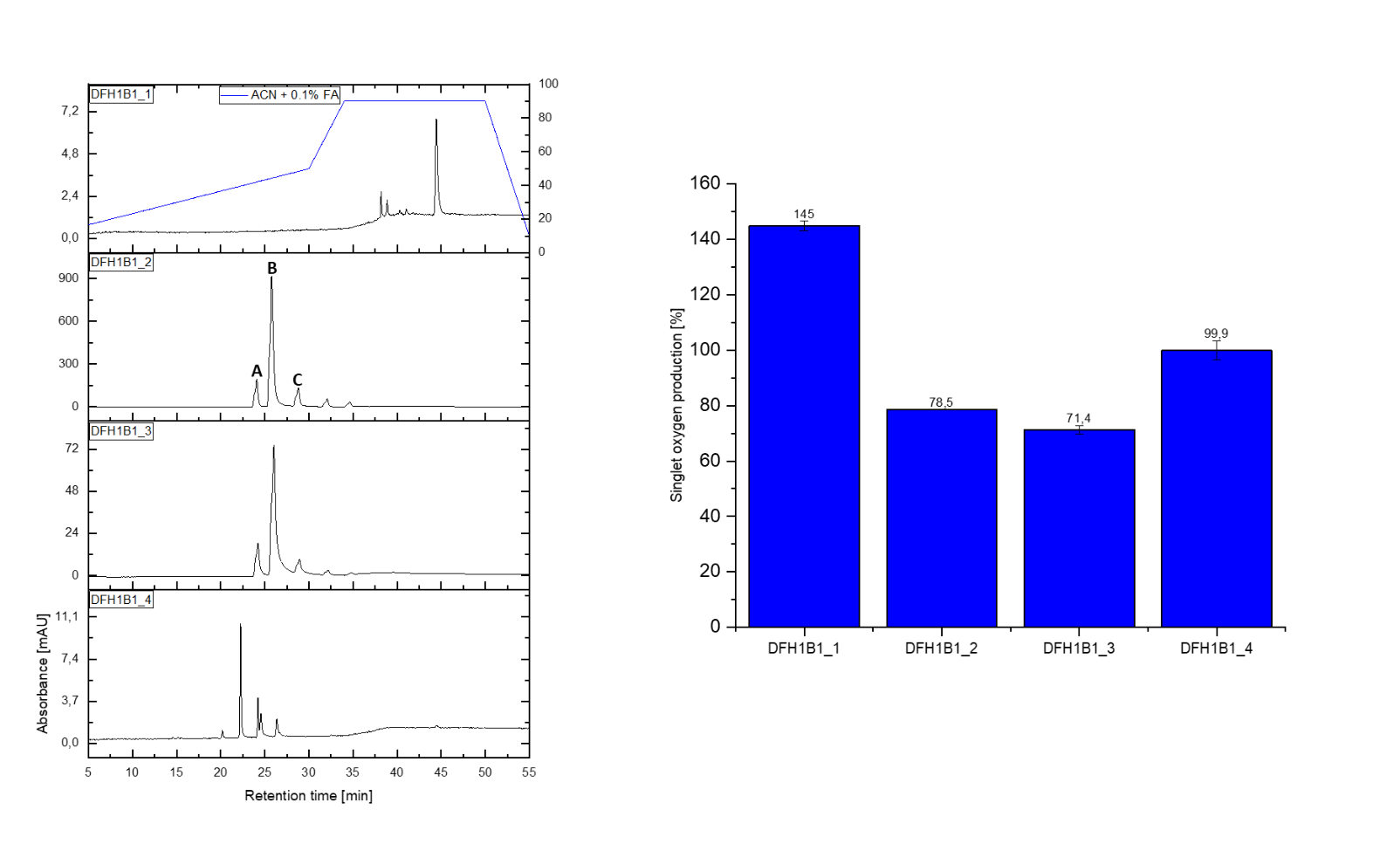


**Figure S7.** On the left side, the chromatograms of the fractions DFH1B1_1-4 recorded at λ = 468 nm are shown. All fractions were dissolved in DMSO (c = 1 mg/mL) for analysis (A … Dermolutein (1.10.3), B … Dermorubin (1.10.3), C … 5-Chlordermorubin). Stationary phase: Phenomenex Synergi MAX-RP, Mobile phase: H_2_O/ACN (+ 0.1 % FA). On the right side, the results of the DMA-assay (Singlet oxygen production (%)) of the fractions DFH1B1_1-2 are depicted.

- - 1. Preparative HPLC of DFH1B1_2 and DFH1B1_3

The fractions DFH1B1_2 (m = 5.08 mg) and DFH1B1_3 (m = 15.44 mg), which were obtained via dry column vacuum chromatography of DFH1B3 (chapter 1.10.2), were dissolved in methanol and separated via preparative HPLC chromatography. The mobile phase (A) was water, (B) consisted of acetonitrile and 0.1 % formic acid. Elution was performed in gradient mode starting with 10 % B to 90 % B from 0 to 13 min, 90 % B to 90 % B from 13-18 min, 90 % B to 10 % B from 18-18.1 min, 10 % B to 10 % B from 18.1-23 min, followed by 6 min of re-equilibration with 90 % A. The detector was set to 468 nm, and flow rate, sample volume and column temperature were adjusted to 1.5 mL/min, 50 µL and room temperature (22 °C), respectively. Three fractions (DFH1B2_1 to DFH1B2_3) were collected, evaporated to dryness at 40 °C under reduced pressure, and stored in a desiccator. The yields as well as the colors of the fractions can be seen below (Table S4).

**Table S4.** Yields and colors of the fractions DFH1B2_1-3.

| Fraction | Yield [mg] | Colour |
| --- | --- | --- |
| DFH1B2_1 | 1.8 | yellow |
| DFH1B2_2 | 4.5 | red |
| DFH1B2_3 | 0.2 | dark red |

All fractions were analyzed via HPLC-MS (Figure S8).

**Figure S8.** Chromatograms of the fractions DFH1B2_1 and DFH1B2_2 recorded at λ = 468 nm. Both fractions were dissolved in DMSO (c = 1 mg/mL) for analysis. Stationary phase: Phenomenex Synergi MAX-RP, Mobile phase: H_2_O/ACN (+ 0.1 % FA).

- 1. Structure characterization of the isolated compounds
     1. (-)-7,7’-Biphyscion (1) [CAS Registry Number: 39772-01-5]

The orange solid was obtained (yield = 0.04 %) by a targeted isolation from dried fruiting bodies as described in the previous chapter (1.8). M.p.: decomposition above 350°C (> 350 °C lit.); [α]D25=−178 (c=0.1 mg/mL in CHCl_3_) (lit.: [α]D25=−174 (c=0.1 mg/mL in CHCl_3_)); R_f_=0.94 (SiO_2_; Mobile phase: Toluene:MeOH:EtOAc:FA = 94:2.5:2.5:1); ^1^H-NMR (400 MHz, CDCl_3_, 25°C) δ=12.45 (s, 2H; OH), 12.09 (s, 2H; OH), 7.67 (d, *J*=1.14 Hz, 2H; CH-4), 7.55 (s, 2H; CH-5), 7.10 (m, 2H; CH-2), 3.97 (s, 6H; OCH_3_), 2.47 (s, 6H; CH_3_) ppm; IR (ART): $\boldsymbol{\nu}^{\sim}$=1673 (w) (C=O), 1619 (m) (C=C), 1598 (m) (C=C), 1558 (m) (C=C) cm^-1^; MS (ESI, negative mode 4.5 kV) m/z (%): 565.2 (100) [M-H]^-^; UV-Vis (MeOH): $\boldsymbol{\lambda}_{\boldsymbol{max}}$ (ε)=202 (5606), 283 (3974), 456 nm (3040 mol^-1^ dm^3^ cm^-1^).

Spectral data – (-)-7,7´-Biphyscion (**1**): The IR spectrum (Figure S9), the UV/Vis spectrum (Figure S10), and a set of NMR-spectra of (**1**) (Figure S11, Figure S12, Figure S13) are displayed below.

**Figure S9.** IR spectrum: (–)-7,7´-Biphyscion (**1**).


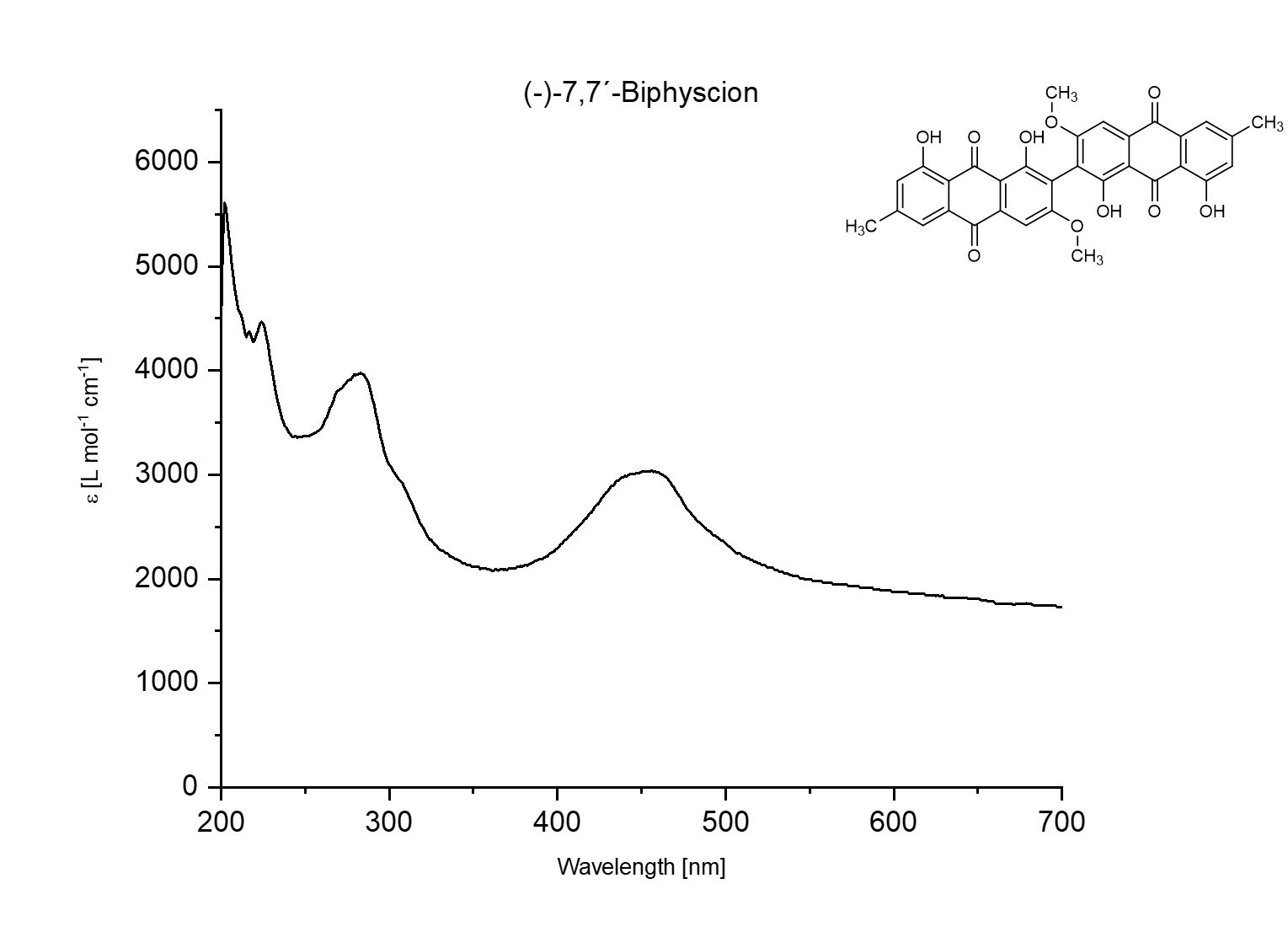


**Figure S10.** UV/Vis spectrum of (-)-7,7´-biphyscion (**1**) in MeOH.


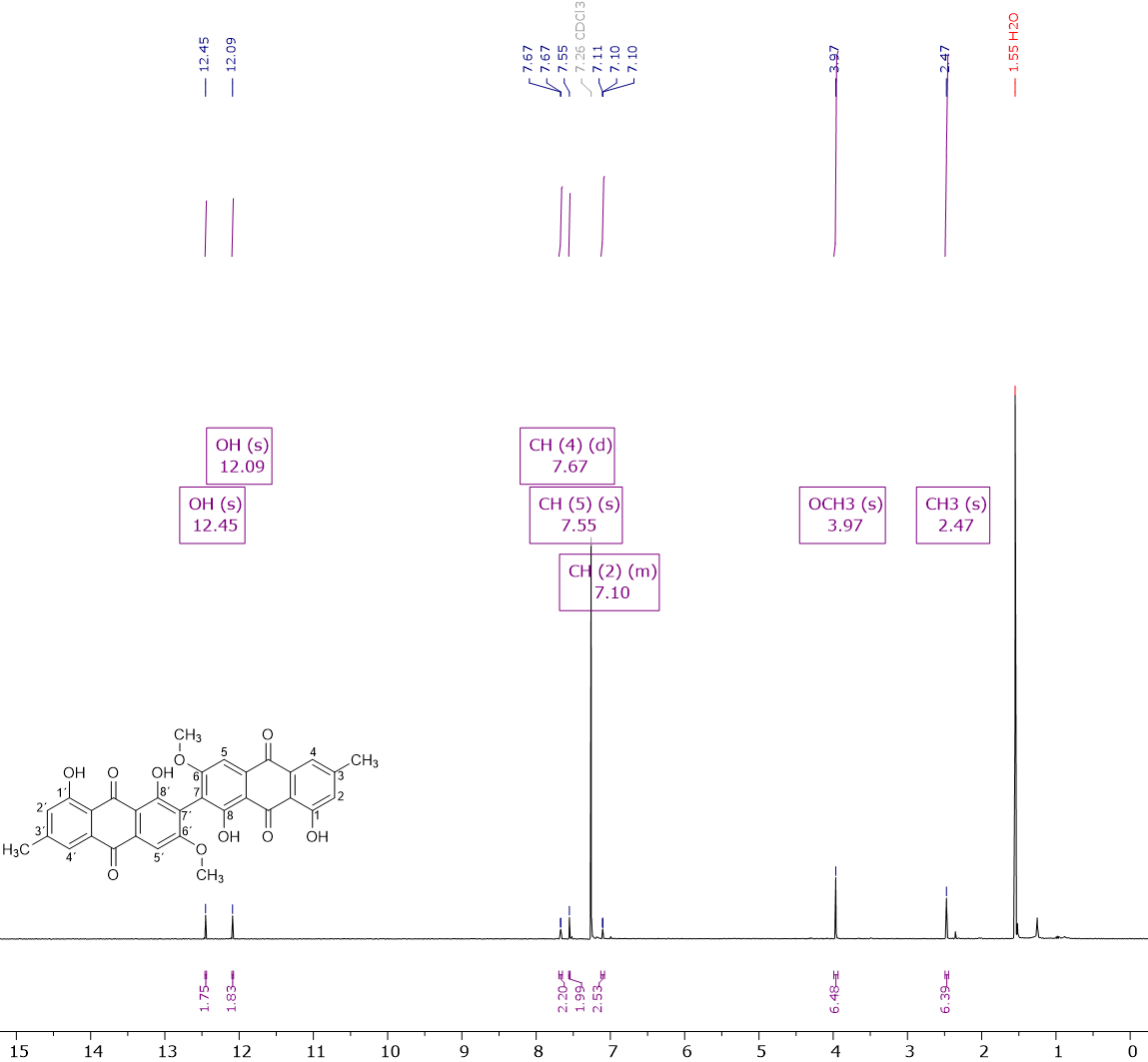


**Figure S11.** ^1^H-NMR (400 MHz, CDCl_3_, 25 °C): (-)-7,7´-Biphyscion (**1**).


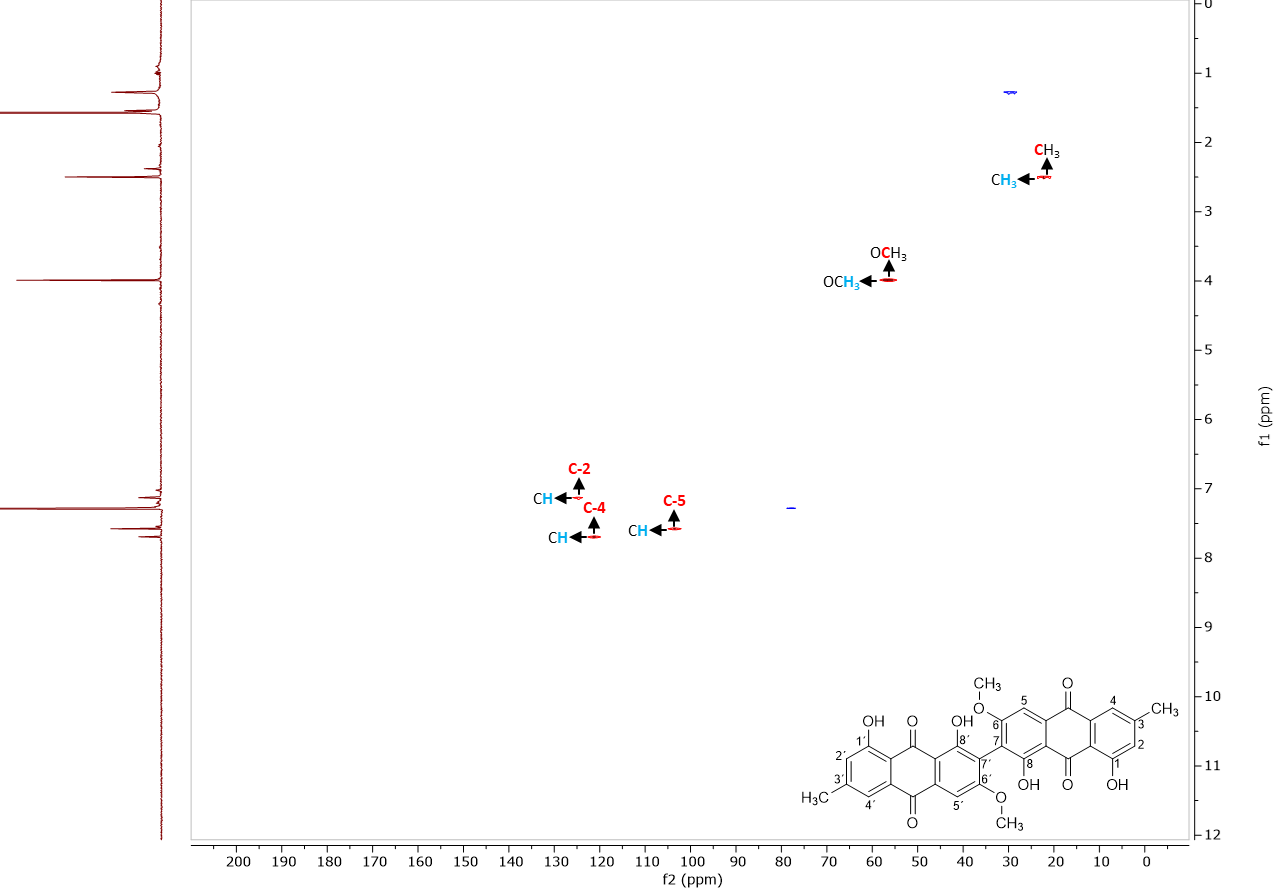


**Figure S12.** HSQC (400 MHz, CDCl_3_, 25 °C): (-)-7,7´-Biphyscion (**1**).


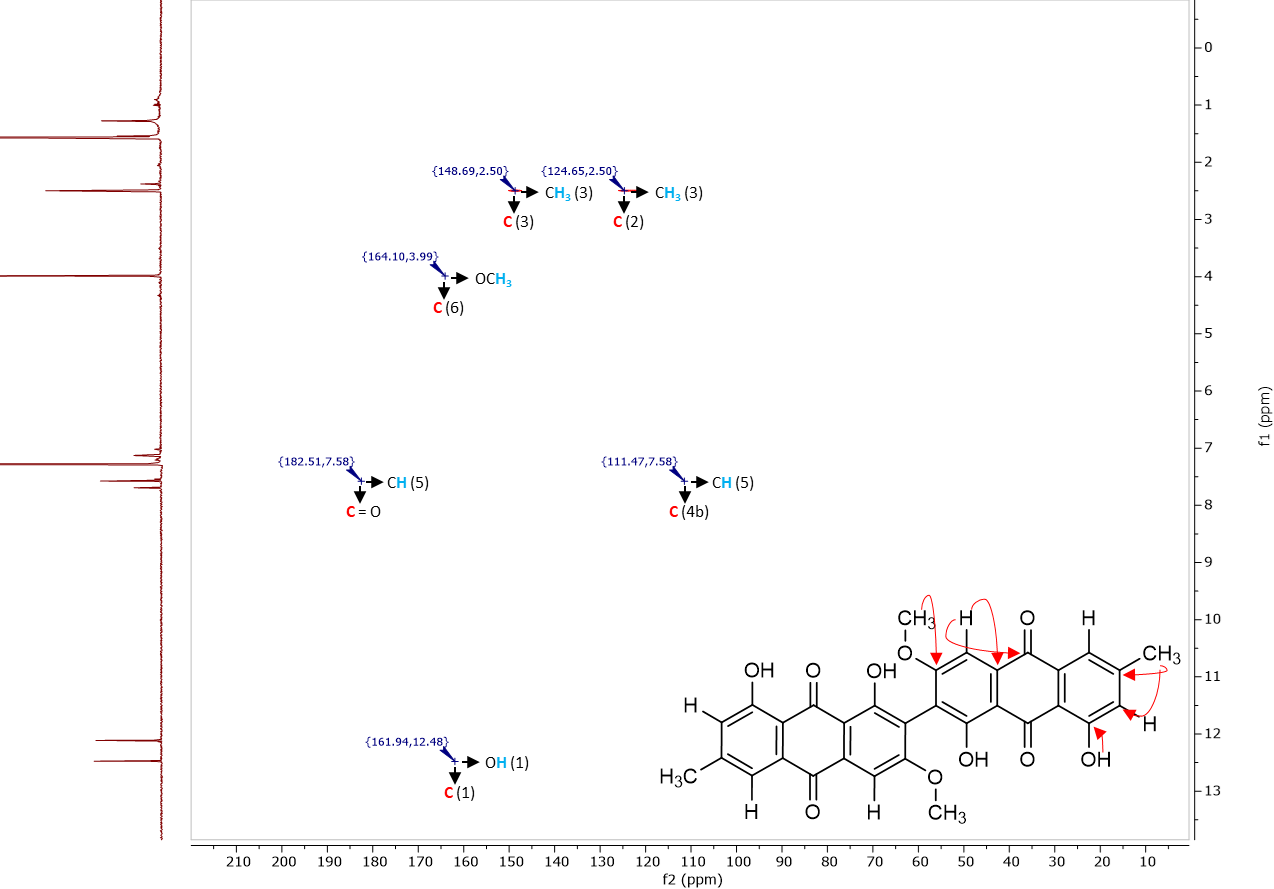


**Figure S13.** HMBC (400 MHz, CDCl_3_, 25 °C): (-)-7,7´-Biphyscion (**1**).

- - 1. Dermorubin (3) [CAS Registry Number: 26071-14-7]

The red solid was obtained (yield = 0.01 %) by a targeted isolation from dried fruiting bodies as described in chapter 1.10. M.p.: decomposition above 265 °C (lit.: decomposition above 300 °C); R_f_=0.45 (SiO_2_; Mobile phase: Toluene:MeOH:EtOAc:FA = 94:2.5:2.5:1); ^1^H-NMR (400 MHz, [D_6_]DMSO, 25 °C, TMS): δ=13.15 (s, 1H; COOH), 7.29 (d, *J*=2.26 Hz, 1H; CH), 6.87 (d, *J*=2.22 Hz, 1H; CH), 3.91 (s, 3H; OCH_3_), 2.19 (s, 3H; CH_3_) ppm; IR (ART): $\boldsymbol{\nu}^{\sim}$=3680-2180 (w, br) (OH), 1707 (w) (C=O), 1561 (m) (C=C) cm^-1^; MS (ESI, negative mode 4.5 kV) m/z (%): 343.1 (100) [M-H]^-^; UV-Vis (MeOH): $\boldsymbol{\lambda}_{\boldsymbol{max}}$ (ε)=234 (15019), 279 (13659), 481 nm (7867 mol^-1^ dm^3^ cm^-1^).

Spectral data – Dermorubin (**3**):

Displayed below are the IR-Spectrum (Figure S14), the ^1^H-NMR spectrum (Figure S15), and the UV/Vis spectrum (Figure S16) of dermorubin (**3**).


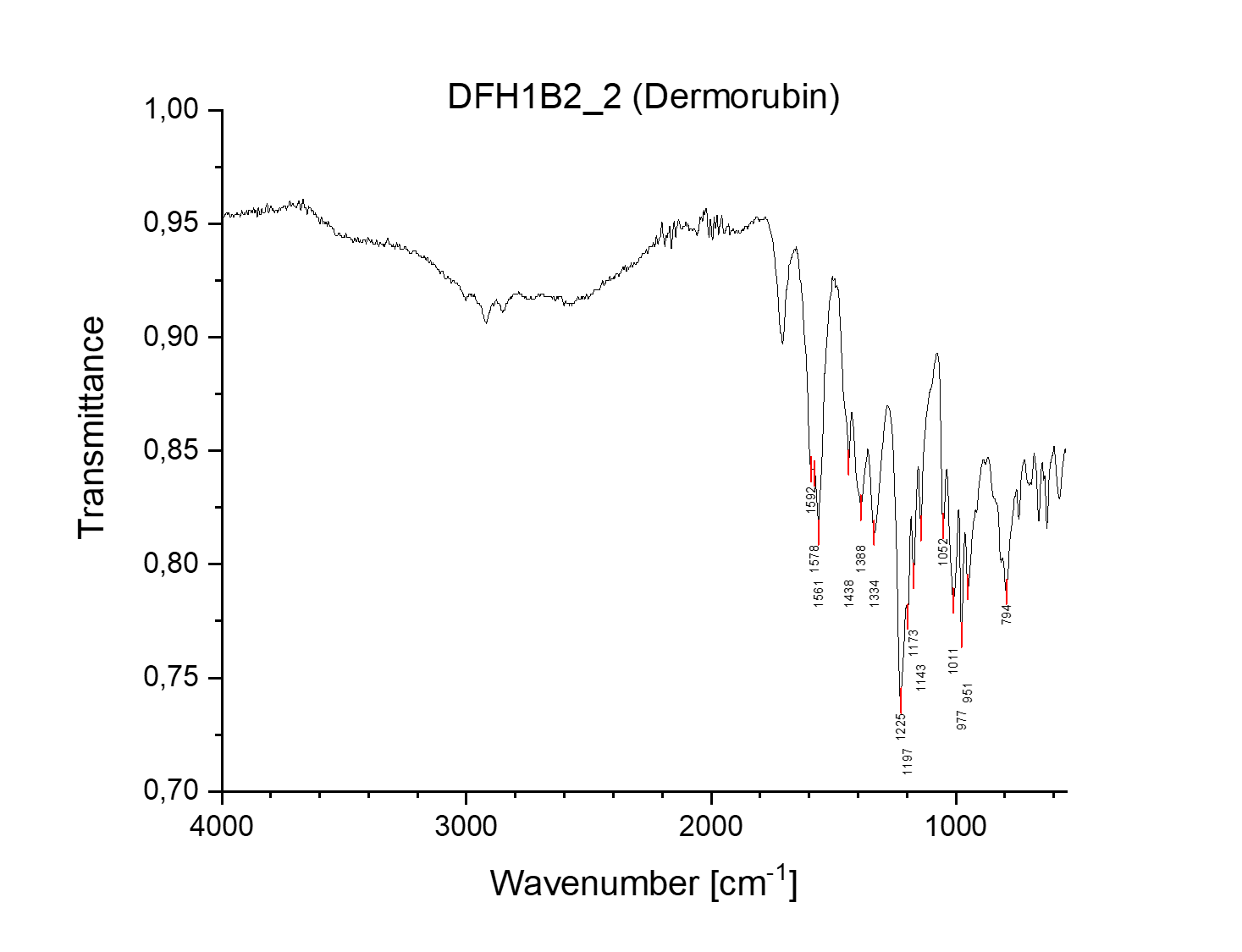


**Figure S14.** IR spectrum: Dermorubin (**3**).


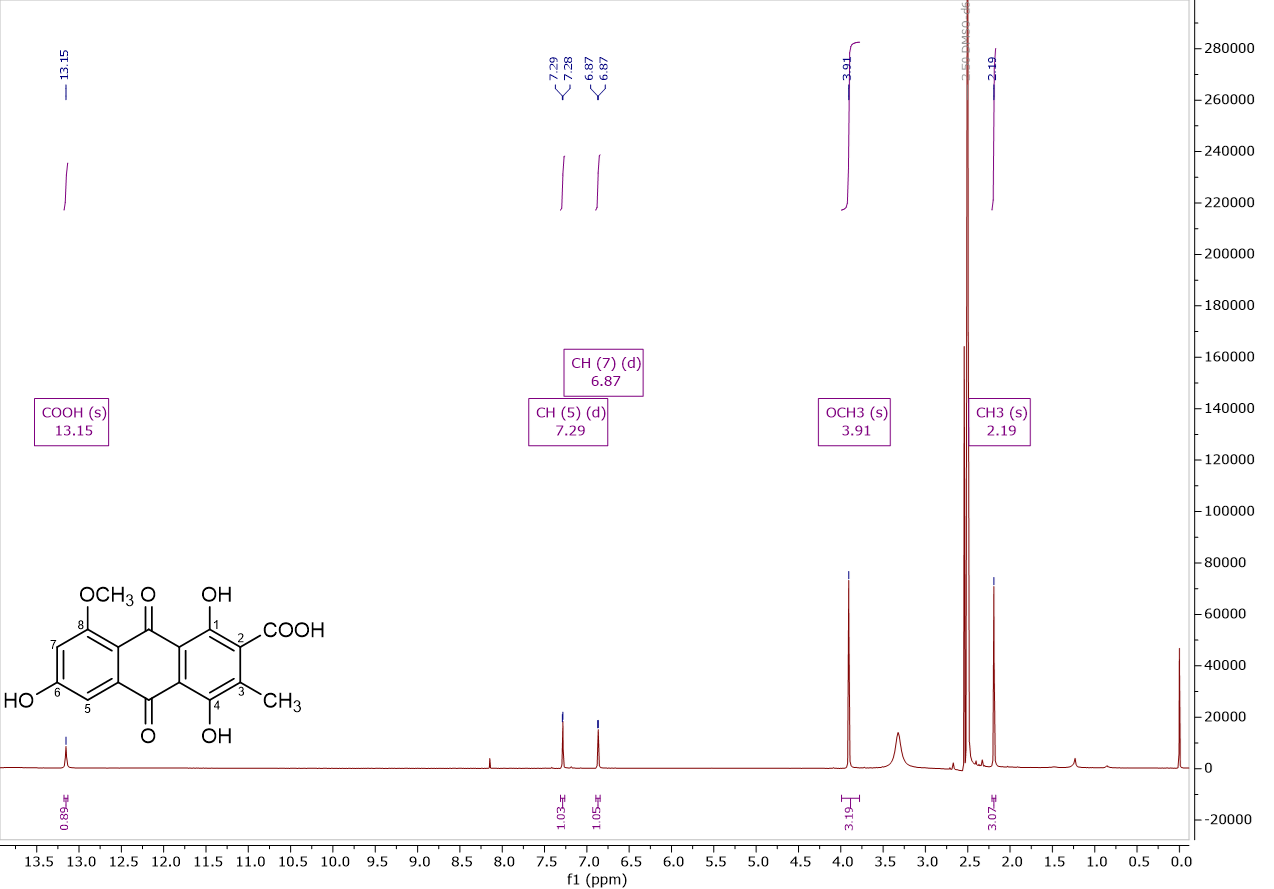


**Figure S15.** ^1^H-NMR (400 MHz, [D_6_] DMSO, 25 °C, TMS): Dermorubin (**3**).


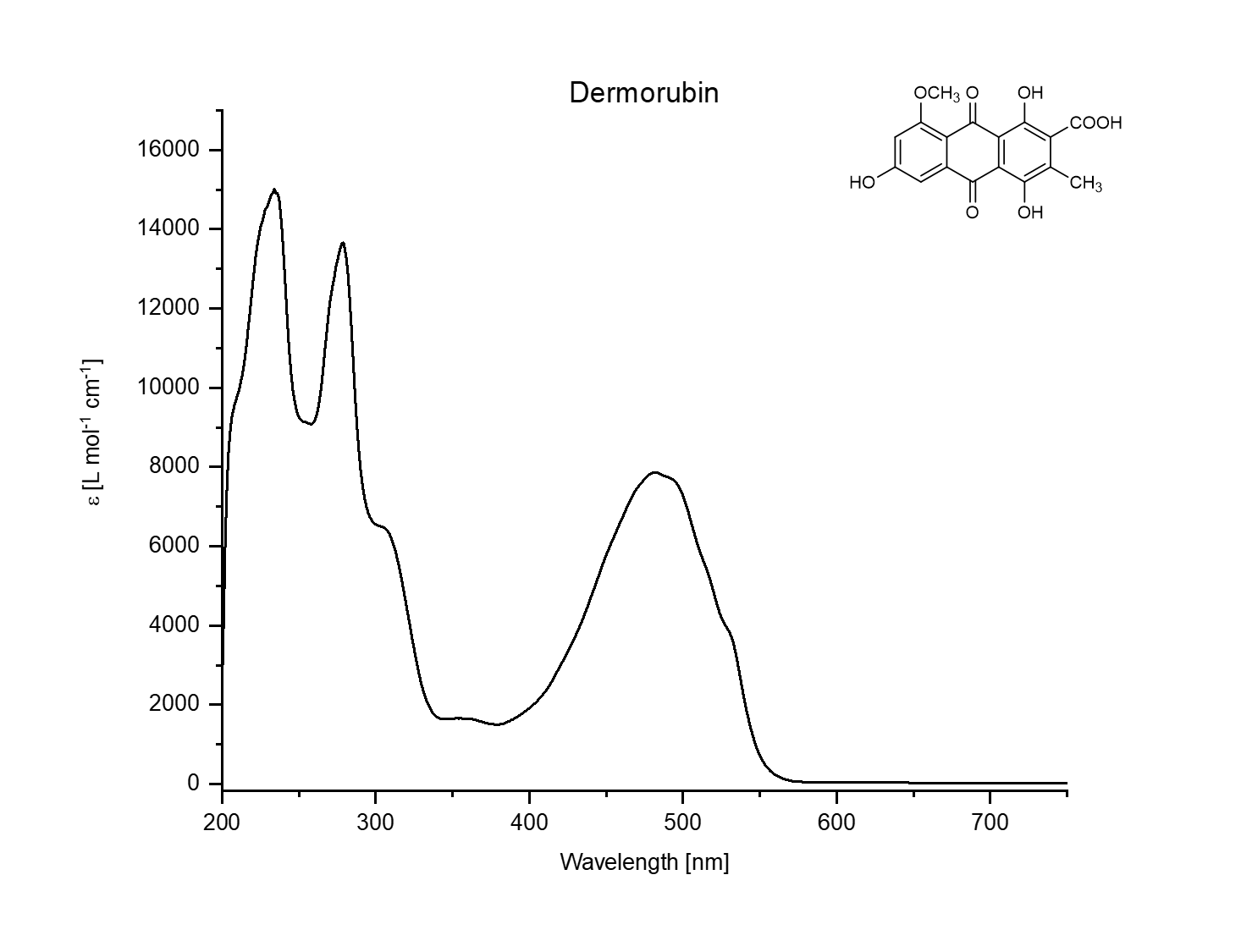


**Figure S16.** UV/Vis spectrum of dermorubin (**3**) in MeOH.

- - 1. Dermolutein (2) [CAS Registry Number: 26071-13-6]

The orange solid was obtained (yield = 0.02 %) by a targeted isolation from dried fruiting bodies as described in chapter 1.10. M.p.: decomposition above 295 °C (lit.: indistinct / decomposition at 270 °C); R_f_=0.36 (SiO_2_; Mobile phase: Toluene:MeOH:EtOAc:FA = 94:2.5:2.5:1); ^1^H-NMR (400 MHz, [D_6_]DMSO, 25 °C, TMS): $\boldsymbol{\delta}$ = 14.04 (COO*H*, s, 1H), 11.25 (O*H*, s, 1H), 7.40 (C*H* (4), s, 1H), 7.19 (C*H* (5), d, J = 2.17 Hz, 1H), 6.85 (C*H* (7), d, J = 2.23 Hz, 1H), 3.90 (OCH_3_, s, 3H), 2.42 (CH_3_, s, 3H) ppm; ^13^C-NMR (151 MHz, DMSO) δ =186.5 (*C*=O, C9), 184.6 (*C*=O, C10), 167.0 (*C*=O, COOH), 164.8 (*C*_ar_OH, C5), 163.5 ((*C*_ar_OMe, C8), 155.5 (*C*_ar_OH, C1), 152.6 (*C*_ar_CH_3_, C3), 136.4 (*C*_quart_, C14), 131.2 (*C*_quart_, C13), 124.0 (*C*_ar_COOH, C2), 112.8 (*C*_quart_, C12), 111.2 (*C*_quart,_ C11), 110.7 (*C*_ar_H, C4), 106.9 (*C*_ar_H, C6), 105.5 (*C*_ar_H, C7), 56.3 (*C*H_3_, OMe), 18.0 (*C*H_3_) ppm; IR (ART) = 3640-2200 (OH; w), 1708 (C=O; w), 1672 (C=O; w), 1623 (C=C; m), 1593 (C=C; m), 1560 (C=C; m) cm^-1^; MS (ESI, negative mode 4.5 kV) m/z (%): 327.2 (100) [M-H]^-^; UV-Vis (MeOH, ε) = 228 (21114), 275 (19404), 427 (7851) nm.

Spectral data – Dermolutein (**2**): The IR Spectrum (Figure S17), the ^1^H-NMR spectrum (Figure S18), and the UV/Vis spectrum (Figure S19) of dermolutein (2) are displayed below.


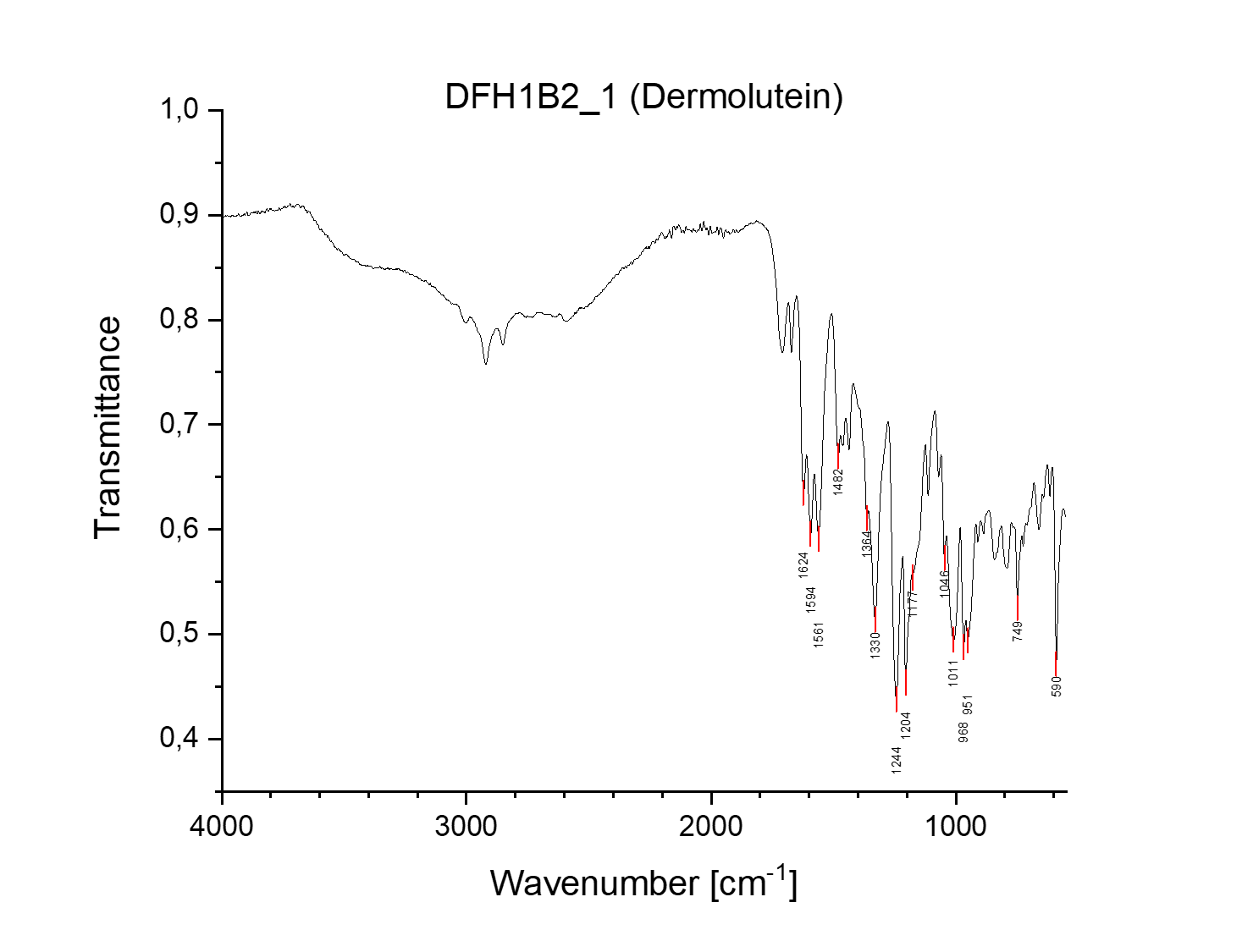


**Figure S17.** IR spectrum: Dermolutein (**2**).


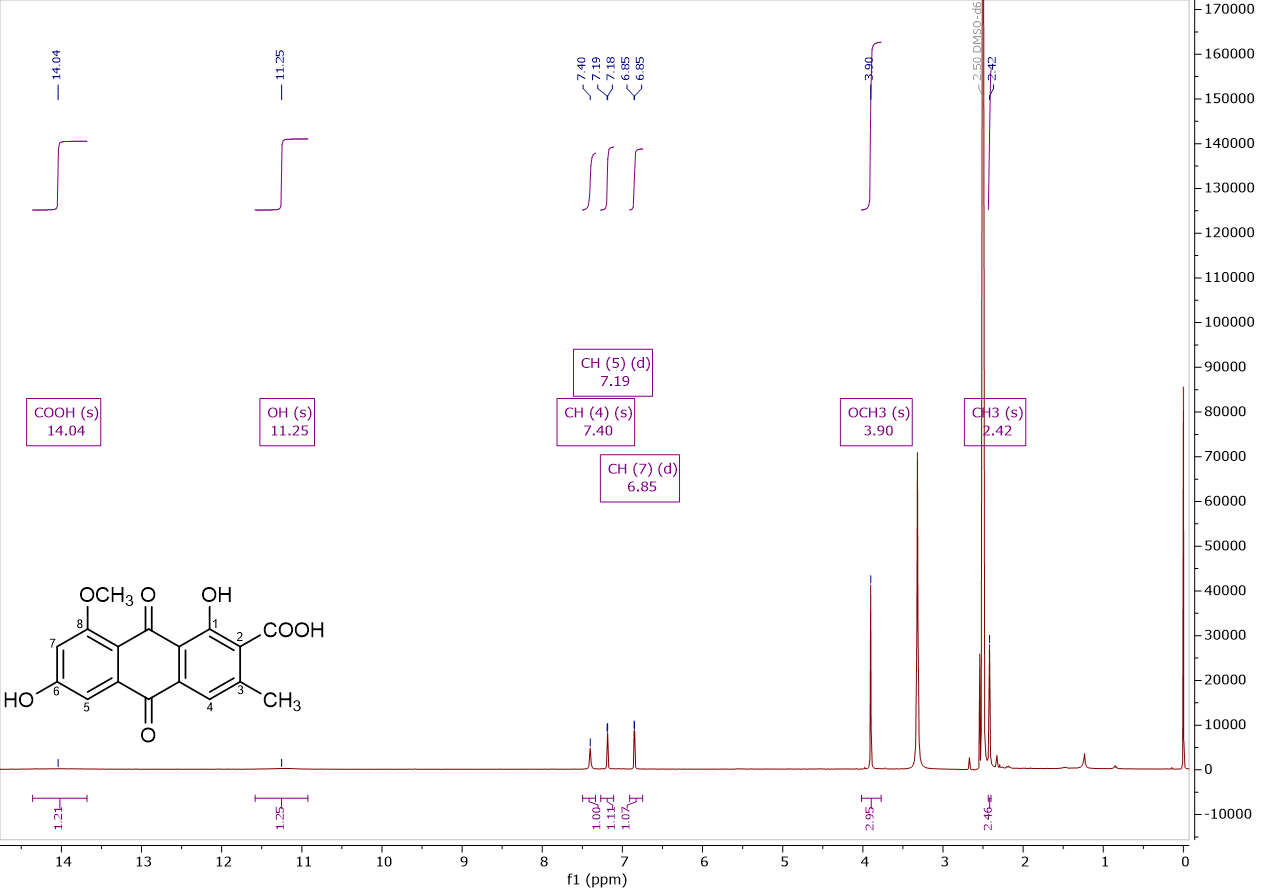


**Figure S18.** ^1^H-NMR (400 MHz, [D6] DMSO, 25 °C, TMS): Dermolutein (**2**).


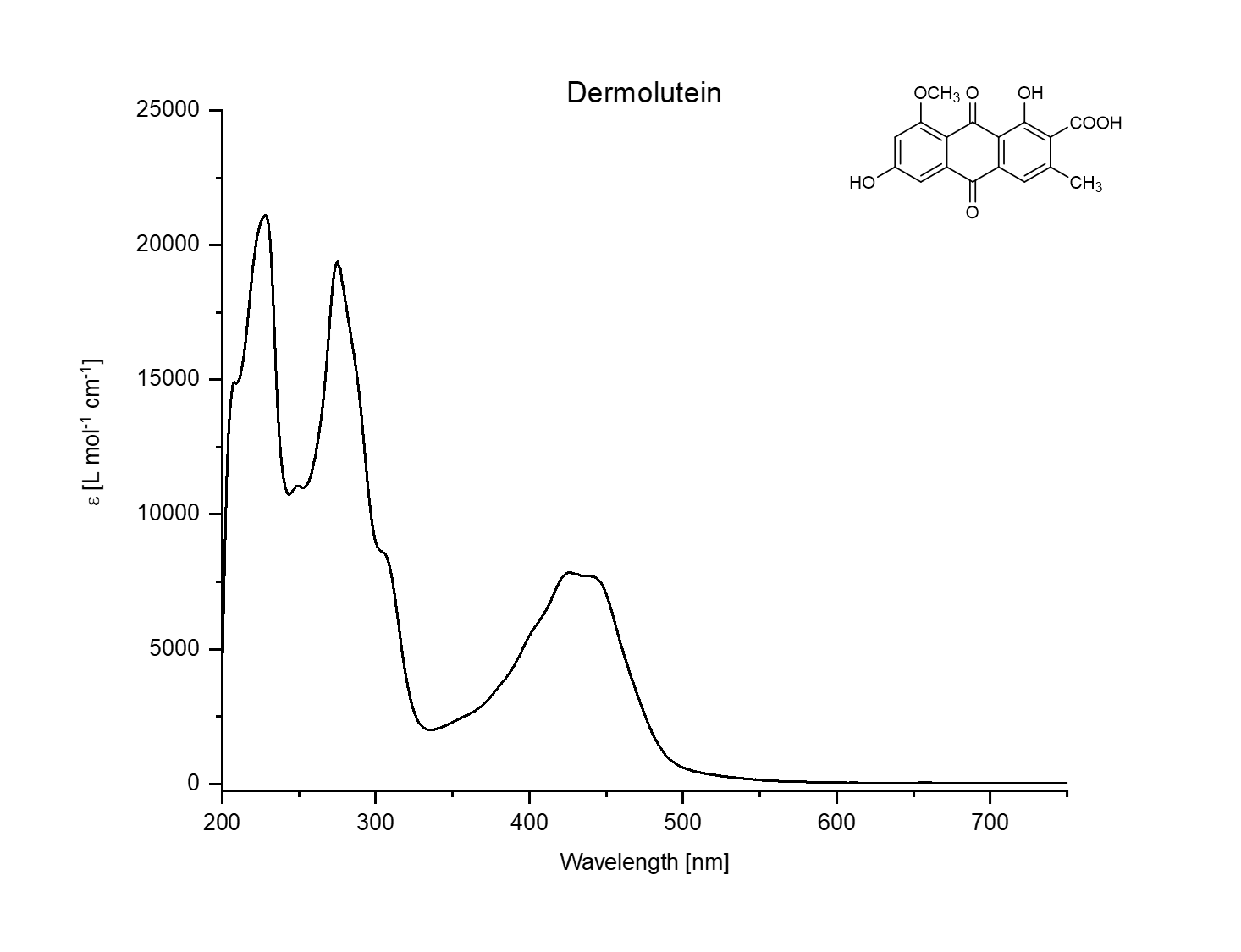


**Figure S19.** UV/Vis spectrum of dermolutein (**2**) in MeOH.

1. Phytochemical Profiling and Molecular Networking

In order to support the bioactivity-guided isolation based on the DMA-assay, a feature-based molecular network was created and annotated. In detail, an UHPLC-DAD-MS/MS analysis of the methanolic extract of *C. uliginosus*, *C. phoeniceus*, the fractions (i.e. diethyl ether (DFH1A_Et_2_O), ethyl acetate (DFH1A_EtOAc), n-butanol (DFH1A_BuOH), water (DFH1A_H_2_O) (see chapter 1.7)), and the blank was done. The methanolic extract of *C. phoeniceus* was added to the set to assign less active light-absorbing features.

The chromatographic separation was performed on a Waters Acquity UPLC system interfaced to a Q-Exactive Focus mass spectrometer (Thermo Scientific, Bremen, Germany), using a heated electrospray ionization (HESI-II) source as described elsewhere.^5^ The optimized HESI-II parameters were as follows: source voltage, 2.5 kV (neg); sheath gas flow rate (N2), 55 units; auxiliary gas flow rate, 15 units; spare gas flow rate, 3.0; capillary temperature, 350.00°C, S-Lens RF Level, 45.

- 1. MS Data Pretreatment

The MS data were converted from .RAW (Thermo) standard data format to .mzXML format using the MSConvert software, part of the ProteoWizard package.^6^ The converted files were treated using the MZmine software suite v. 2.38.^7^

The parameters were set as follows: the centroid mass detector was used for mass detection with the noise level set to 1.0E5 for MS level set to 1, and to 1 for MS level set to 2. The ADAP chromatogram builder was used and set to a minimum group size of scans of 5, minimum group intensity threshold of 5E4, minimum highest intensity of 5E4 and *m/z* tolerance of 8.0 ppm. For chromatogram deconvolution, the algorithm used was the wavelets (ADAP). The intensity window S/N was used as S/N estimator with a signal to noise ratio set at 10, a minimum feature height at 1E5, a coefficient area threshold at 10, a peak duration ranges from 0.02 to 0.5 min and the RT wavelet range from 0.00 to 0.06 min. Isotopes were detected using the isotopes peaks grouper with a *m/z* tolerance of 8.0 ppm, a RT tolerance of 0.01 min (absolute), the maximum charge set at 2 and the representative isotope used was the most intense. An adduct (Na^+^, K^+^, NH_4_^+^, CH_3_CN^+^, CH_3_OH^+^, C_3_H_8_O^+^ (IPA^+^)) search was performed with the RT tolerance set at 0.1 min and the maximum relative peak height at 500 %. Utilizing an in-house database of fungal pigments described in dermocyboid Cortinarii, dereplication was done whereby a m/z tolerance of 8 ppm was applied. Peak alignment was performed using the join aligner method (*m/z* tolerance at 8 ppm), absolute RT tolerance 0.05 min, weight for *m/z* at 8 and weight for RT at 10. The peak list was gap-filled with the same RT and *m/z* range gap filler (*m/z* tolerance at 8 ppm). Eventually the resulting aligned peaklist was filtered using the peak-list rows filter option in order to keep only features associated with MS2 scans.

- 1. Molecular Networks Generation

A feature based molecular network (<https://ccms-ucsd.github.io/GNPSDocumentation/featurebasedmolecularnetworking/>) was created utilizing the .mgf file resulting from the MZmine treatment (see chapter 2.1). This network analysis allows -based on the LC experiment- the separation of isomers as it keeps the retention time and the exact mass information of each detected feature. In detail, spectral data was uploaded on the GNPS molecular networking platform. A molecular network was created using the online workflow (https://ccms-ucsd.github.io/GNPSDocumentation/) on the GNPS website (http://gnps.ucsd.edu). The precursor ion mass tolerance was set to 0.2 Da and a MS/MS fragment ion tolerance of 0.5 Da. A network was then created where edges were filtered to have a cosine score above 0.7 and more than 5 matched peaks. Further, edges between two nodes were kept in the network if and only if each of the nodes appeared in each other's respective top 6 most similar nodes. Finally, the maximum size of a molecular family was set to 100, and the lowest scoring edges were removed from molecular families until the molecular family size was below this threshold. The spectra in the network were then searched against GNPS' spectral libraries. All matches kept between network spectra and library spectra were required to have a score above 0.7 and at least 6 matched peaks.

- 1. In-house Library

An in-house library of fungal anthraquinones was generated based on the work of Gill and Steglich {Gill, 1987 #64}. Chemical structures of anthraquinones and related compounds were drawn with ChemDraw, exported as SMILES code, and their exact masses subsequently determined with a R script/pipeline pilot workflow.

- 1. Annotation by Sirius

Sirius 4.0.1^8^ – a state-of-the-art metabolite annotation solution – was employed as annotation tool. It combines molecular formula calculation and the prediction of a molecular fingerprint of a query compound from its fragmentation tree. The parameters used to proceed selected features were the following for Sirius molecular formula calculation: possible ionization [M+H]^-^, [M+Cl]^-^, [M+Br]^-^, instrument: Orbitrap, ppm tolerance 8 ppm, Top molecular formula candidates: 10, filter: formulas from biological DBs. For the CSI^9^: FingerID step, the parameters were the following: possible adducts: [M-H]^-^, [M+Cl]^-^, [M+Br]^-^, [M+CH2O2-H]^-^ filter: compounds present in PubChem, maximal number of returned candidates: unlimited. Sirius 4.0.1 is available at the following address: <https://bio.informatik.uni-jena.de/software/sirius/>.

- 1. Taxonomically Informed Metabolite Annotation Molecular Networks Generation (ISDB-DNP-Taxo)

The spectral file (.mgf) and attributes metadata (.clustersummary) obtained after the MN step were annotated using the ISDB-DNP with the following parameters: parent mass tolerance 0.005 Da, minimum cosine score 0.2, maximal number of returned candidates: 200. A modified R script including scoring up to the kingdom level was written to proceed to the *taxonomically informed scoring* on ISDB-DNP output and return a reranked table that can be directly loaded in Cytoscape. The initial script is available online (taxo_scorer_user.Rmd) at <https://github.com/oolonek/taxo_scorer>.

- 1. Generation of the Variable "Vis Signal"

In order to primarily investigate study-relevant features (i.e. 468 nm light absorbing metabolites), the related Vis-chromatogram (468 nm) was extracted from the DAD scan. For each feature it was analyzed whether it could be assigned to an absorption peak or not. When it was possible to assign the feature to a peak, the value was set to "1". In case it was not assigned, the value of this variable was set to "0". The result was exported as .csv and later imported into Cytoscape. In total, 231 features are correlated to a Vis-peak. It needs to be mentioned, however, that an observed correlation does not necessarily mean that the feature absorbs light, as many features occur at the same retention time. Nevertheless, the probability is increased that a feature absorbs light – and therewith is a putative light-active metabolite – if it occurs where a peak occurs in the VIS chromatogram.

- 1. Untargeted Molecular Network Investigation

Figure S20 displays the generated molecular network. Each node is colorized by pie-diagram representing the composition and origin of this feature. In detail, a white coloration represents features from *C. phoeniceus*, a dark-gray coloration from the polar fractions (DFH1A_BuOH, DFH1A_H_2_O) of *C. uliginosus* and a light-red hue from apolar fractions (DFH1A_Et_2_O, DFH1A_EtOAc) of *C. uliginosus*. Interestingly, by this approach species specific clusters (e.g. the four clusters with white-nodes for *C. phoeniceus*) were identified, hinting towards a new opportunity to support taxonomy.

In a next step we highlighted nodes with a VIS-Signal by reducing the size of non-VIS feature to a tenth of the size, Figure S21. In total, 231 nodes which can be correlated to a VIS-Signal were detected. Nevertheless, not necessarily each of these 231 features needs to hold absorption properties, as they may just coelute with some other absorptive feature.

Finally, the level of bioactivity was added to aid the visual interpretation (Figure S22). In detail, a bio-scoring approach in analogy to Gaudry et al.^10^ was explored. Therefore, an activity score was calculated and the size of each feature with an activity of above 90 % was set in to 100, above 80 % to 50.

$Activity=Vis Signal* \frac{Feature Area CU}{Feature Area \left( CP+ CU \right)}*\frac{Feature Area (Apolar Fractions CU)}{Feature Area (CP+Apolar Fractions CU+Polar Fractions CU)}$ (Eq.2)

The activity score was calculated (Eq. 2) as product of three factors, which are (1) VIS signal yes/no [value = 0 or 1] (2) the activity of the extracts [value = 0…1], and (3) portion of the highest active fractions (i.e. apolar fractions of the *C.uliginosus* extract) [value = 0…1].

Adding the additional information of the activity of the extracts (Factor 2) is necessary to account for the fact that the fractions are enriched and would be overrated as compared to the non-fractionated extract of *C. phoeniceus*. The identified clusters of interest were annotated by the means of an in-house library, Sirius, and the ISDB-DNP-Taxo. The results of the annotation process are presented in Figure S22, Figure S23-Figure S26. In brief, the results of the untargeted molecular network analysis suggest that the unique anthraquinone-like pigments of *C*. *uliginosus* are most-likely responsible for the observed photo-cytotoxicity effect. It should nevertheless be stressed, that these visualization does not include any information about the content of single features in the whole extract.

***
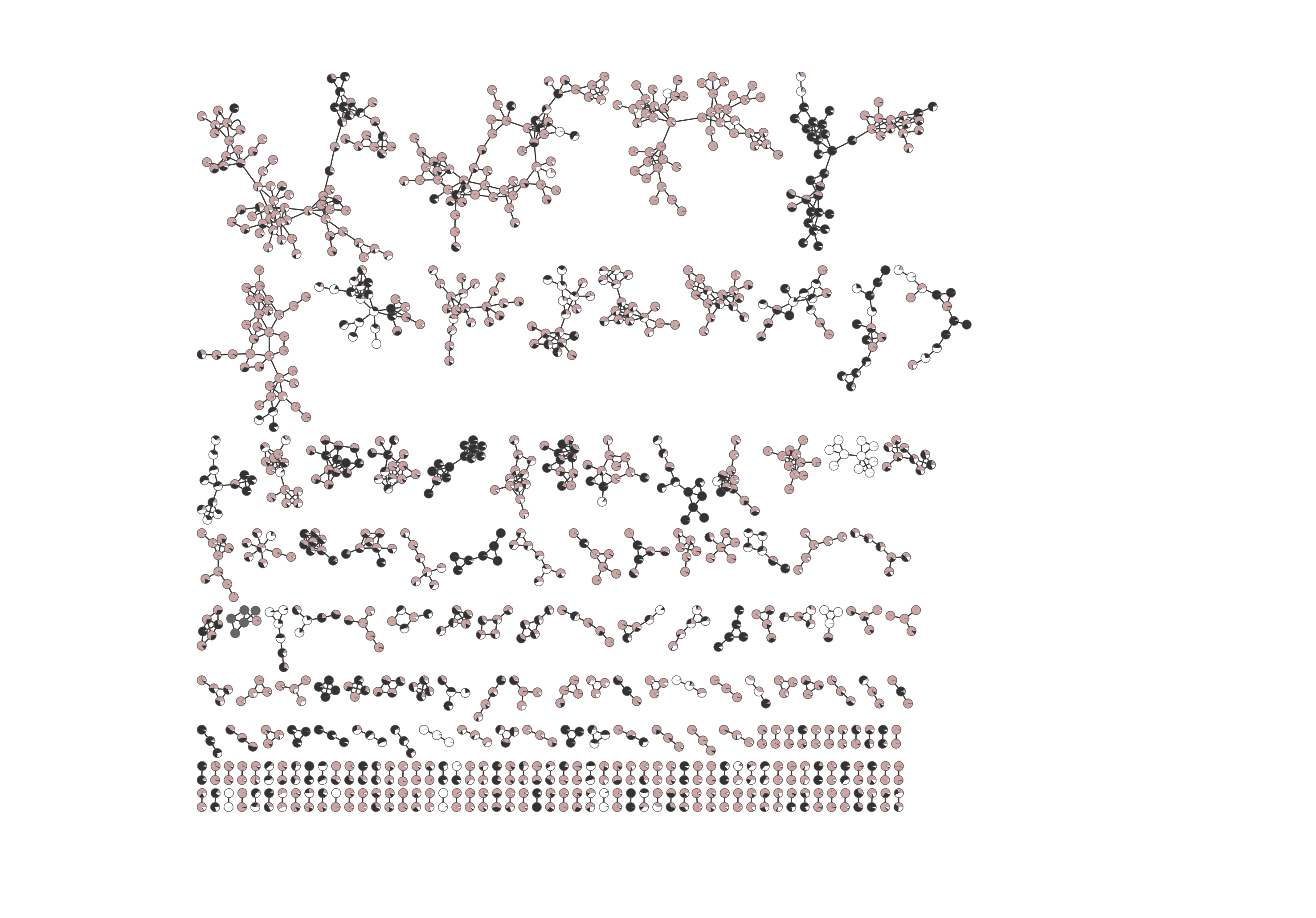
*Figure S20.** Molecular network of *C. phoeniceus* (MeOH Extract = white) and the polar (H_2_O and BuOH, dark-gray) as well as the apolar fractions (EtOAc and Et_2_O, light red) of *C. uliginosus* (MeOH extract).


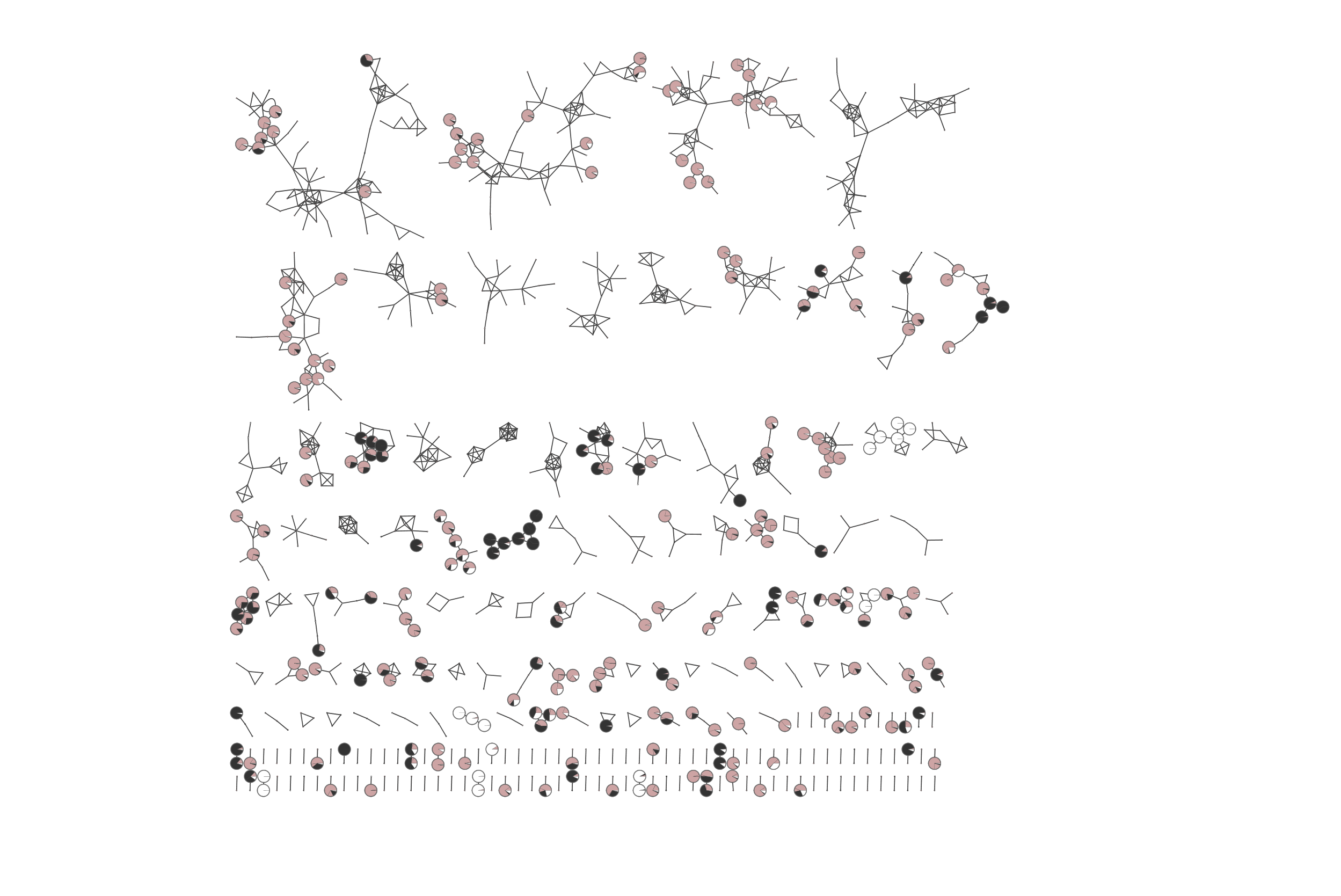
**Figure S21.** Pigment-based Molecular Network of *C. phoeniceus* (MeOH Extract = white) and the polar (H_2_O and BuOH, dark-gray) as well as the apolar fractions (EtOAc and Et_2_O, light red) of *C. uliginosus* (MeOH extract). The size of each node was defined by the absence (size = 1) or presence (size = 100) of a VIS signal (468 nm).


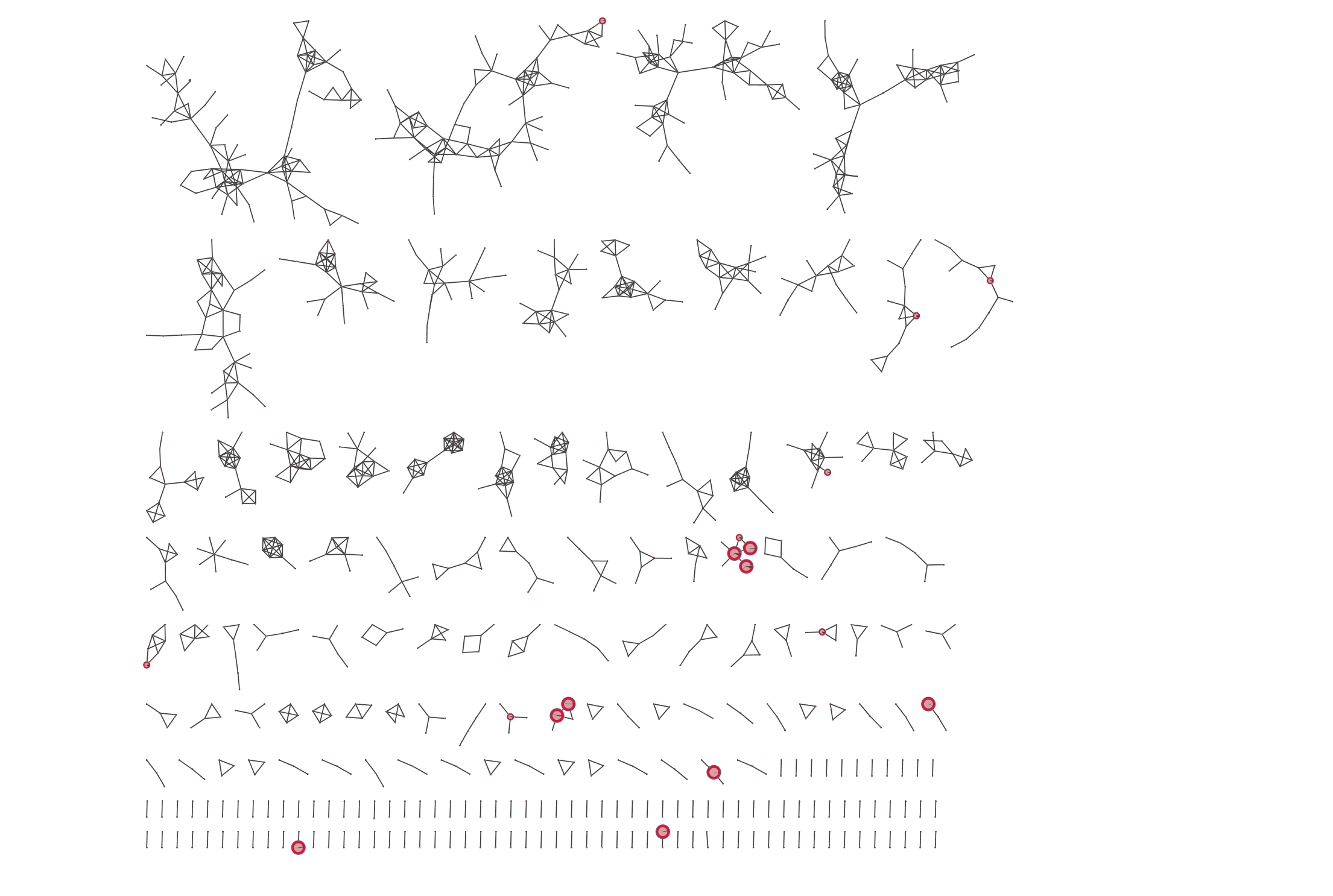


5

6

4

3

2

1

**Figure S22.** Molecular network with highlighted, potentially promising features identified via a bio-scoring approach (see chapter 2.7 for details). As for the previous networks, the light red areas represent the apolar fractions of *C*. *uliginosus,* while the red outer ring indicates a high bioactivity. The size represents the bio-scoring. Activity-Rank >90 % = size 100, Activity-rank > 80 % = size 50, everything below this percentage = size 1. The numbers are the identifier of the investigated, active clusters.

**
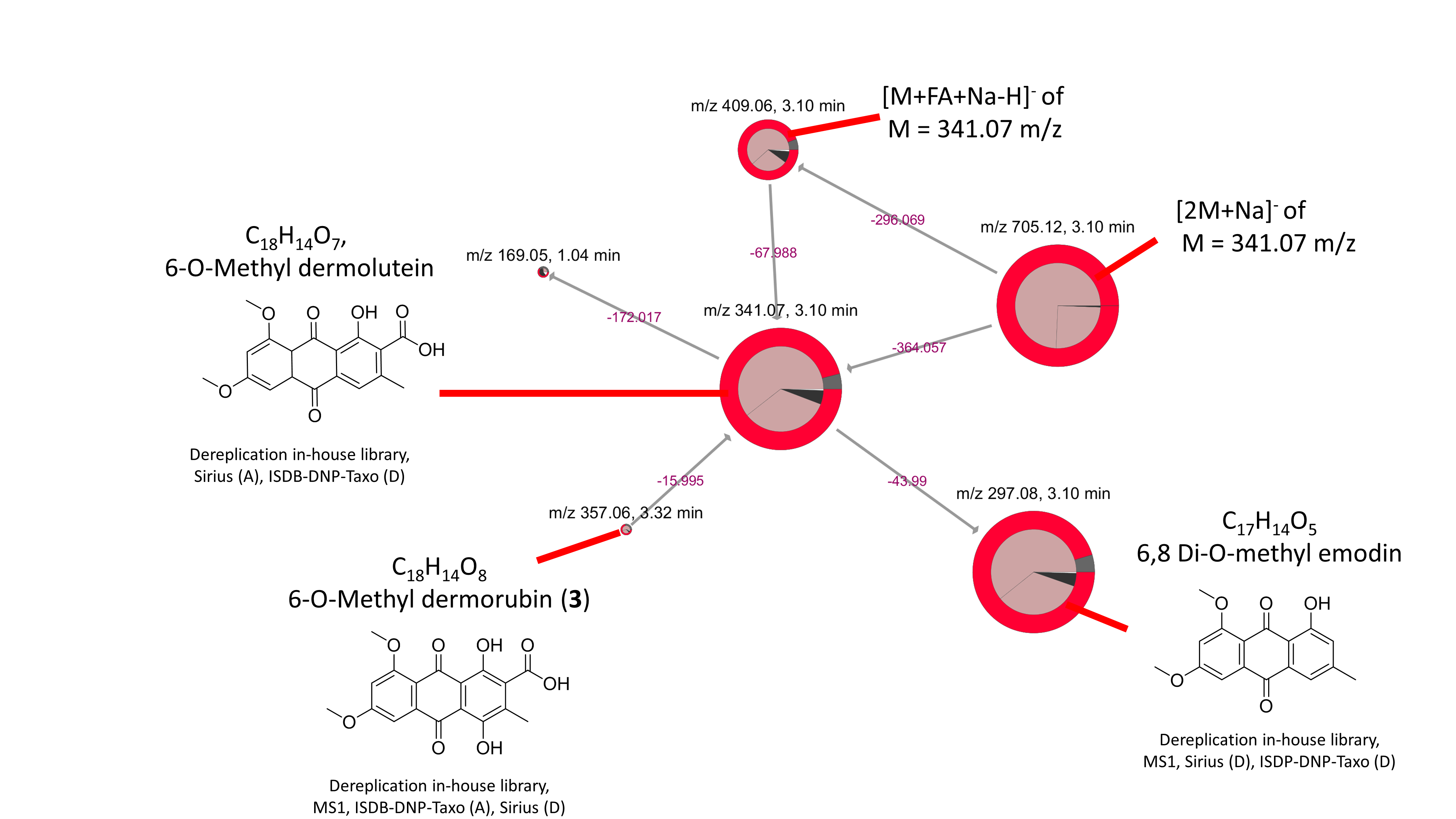
**

**Figure S23.** Annotated Cluster 1. Each node displays its molecular mass (rounded) and retention time. Each edge is labeled with the mass difference, whereby the arrow head indicates the direction. Out of the different molecule annotations for the calculated chemical formula, this structure was chosen which was either commonly identified via all three processes, or was the one previously isolated from dermocyboid Cortinarii (in-house library). The letter in brackets behind the tool indicates the quality of the annotation.


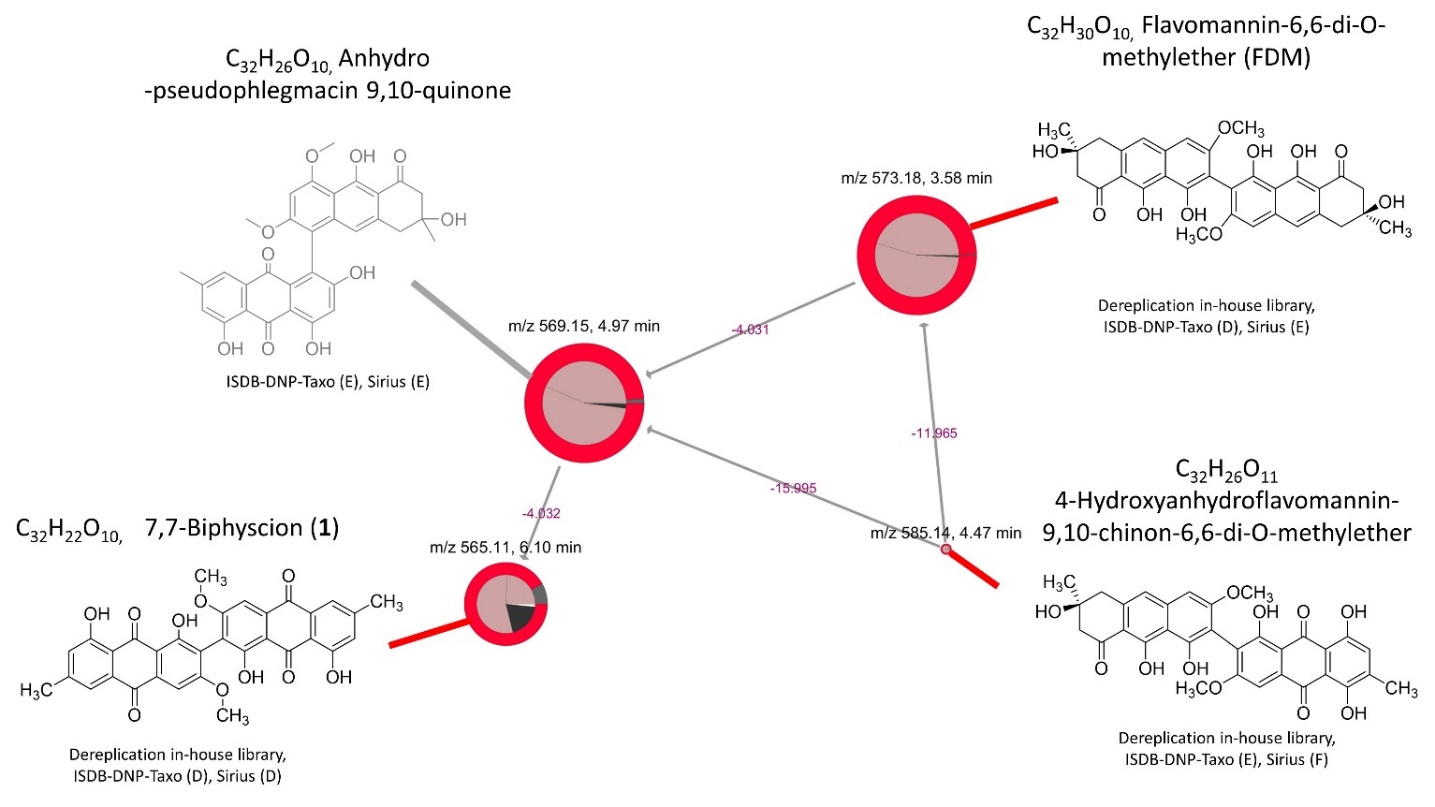


**Figure S24.** Annotated Cluster 2. Each node displays its molecular mass (rounded) and retention time. Each edge is labeled with the mass difference, whereby the arrow head indicates the direction. Out of the different molecule annotations for the calculated chemical formula, this structure was chosen which was either commonly identified via all three processes, or was the one previously isolated from dermocyboid Cortinarii (in-house library). The letter in brackets behind the tool indicates the quality of the annotation.


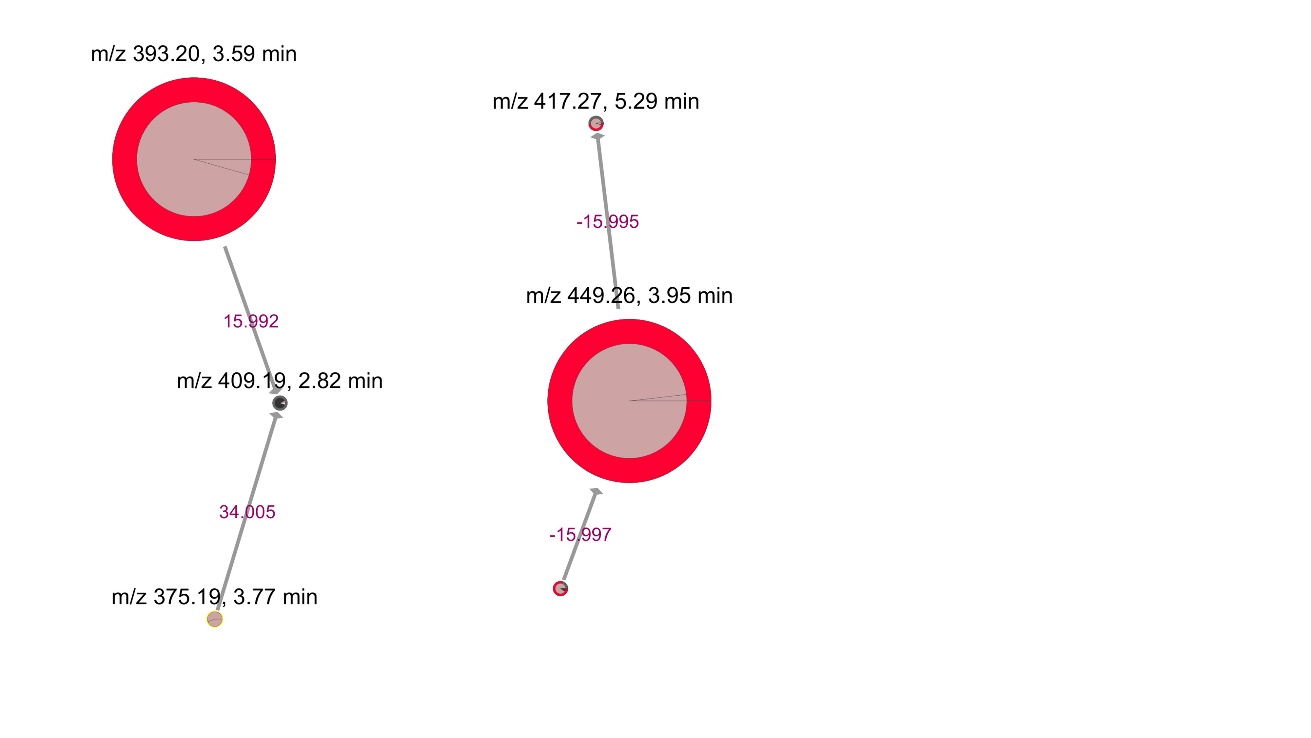


**Figure S25.** Annotated Cluster 3 (left) and 4 (right). Each node displays its molecular mass (rounded) and retention time. Each edge is labeled with the mass difference, whereby the arrow head indicates the direction. These nodes most likely represent unknown features of *C. uliginosus.*


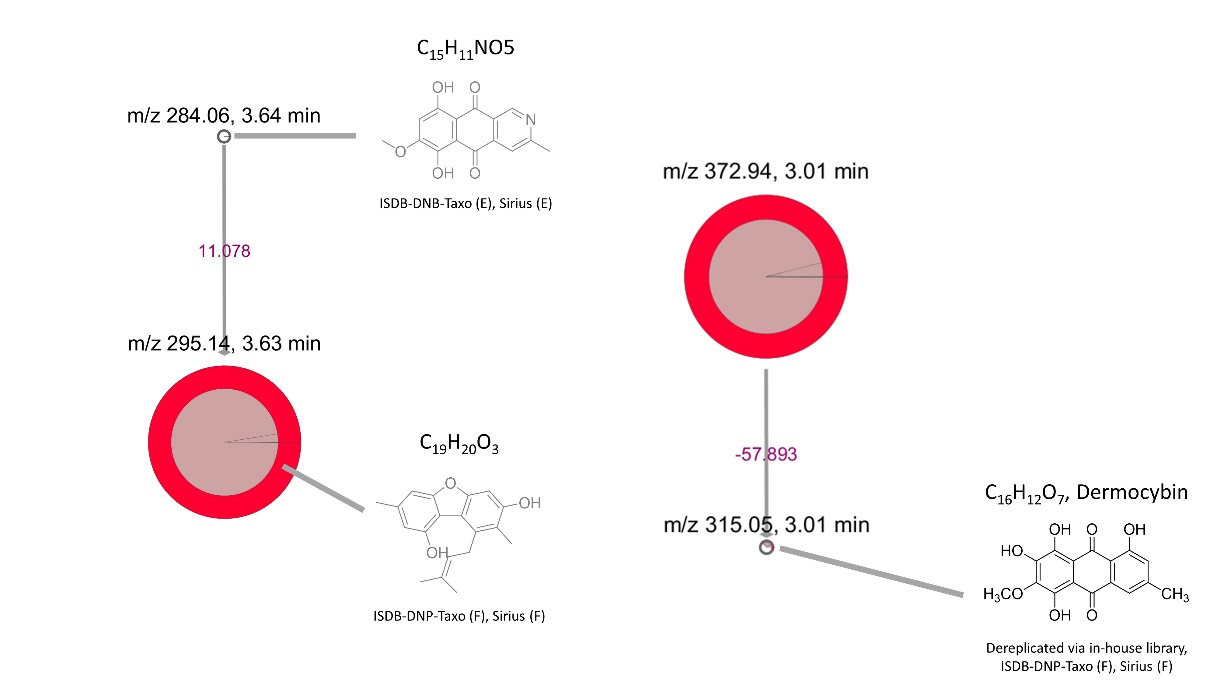


**Figure S26.** Annotated Cluster 5 (left) and 6 (right). Each node displays its molecular mass (rounded) and retention time. Each edge is labeled with the mass difference, whereby the arrow head indicates the direction. Out of the different molecule annotations for the calculated chemical formula, this structure was chosen which was either commonly identified via all three processes, or was the one previously isolated from dermocyboid Cortinarii (in-house library). The letter in brackets behind the tool indicates the quality of the annotation. The gray structures should emphatize that this structures are vague annotations.

- 1. Targeted annotation of the relevant pigments

In parallel to the untargeted bio-activity guided analysis of the molecular network, we wanted to annotate and dereplicate the major pigments of the active EtOAc fraction (Figure S3). As strategy, we first detected the molecular weight of the six relevant pigments by an HPLC-DAD-MS measurement (Figure S28) employing an optimized method (chapter 1.4). The annotated peaks were in a next step correlated to the tandem MS scan attributed to the related feature generated by MZmine (Figure S28). Finally, these tandem MS scans were submitted to (A) Sirius, (B) an in-house database containing the pigments of dermocyboid Cortinarii and (C) an ISDB-DNP. The results are listed in Table S5. Furthermore, the MS scan of pigment 4 is represented in Figure S27 and clearly shows the typical chlorine isotope pattern. The relatively weak hit rate of the ISDB-DNB-Taxo assignment can be explained by the underrepresented number of fungal metabolites in the DNP.^11^

**Table S5.** Targeted annotation of C. uliginosus pigments

|  | RT (HPLC) | Base Peak MS [M±X]^-^ | Feature ID  UPLC-MS | Ion | Annotation  In-house library | Sirius (1^st^ hit) | ISDB-DNP-Taxo  (1st Hit) |
| --- | --- | --- | --- | --- | --- | --- | --- |
| Pigment 1 | 21.6 min | 491.2 m/z | ID697 | [C_22_H_22_O_10_+CH_2_O_2_-H]^-^ | Physcion-8-O-glycoside | AQ glycoside  pos 97 | Isoflavonoid O-glycosides |
| Pigment 2 | 23.7 min | 327.1 m/z | ID426 | [C_17_H_12_O_7_-H]^-^ | Dermolutein | Endocrocin-6-methylether | Endocrocin-6-methylether |
| Pigment 3 | 25.7 min | 343.1 m/z | ID467 | [C_17_H_12_O_8_-H]^-^ | Dermorubin | Dermorubin | Subpsoromic acid |
| Pigment 4 | 28.6 min | 377.0 m/z | ID523 | not identified via Sirius | 5Cl-Dermorubin | NA | NA |
| Pigment 5 | 31.8 min | 341.1 m/z | ID458 | [C_18_H_14_O_7_-H]^-^ | Endocrocin-6,8-Dimethylether | Endocrocin-6,8-Dimethylether | 1,6,8-Trihydroxy-3-propylanthraquinone-2-carboxylic acid |
| Pigment 6 | 44.2 min | 565.1 m/z | ID756 | [C_32_H_22_O_10_-H]^-^ | 7,7’ Biphyscion | 2^nd^ Hit: 7,7’ Biphyscion | Alterporriol Q |


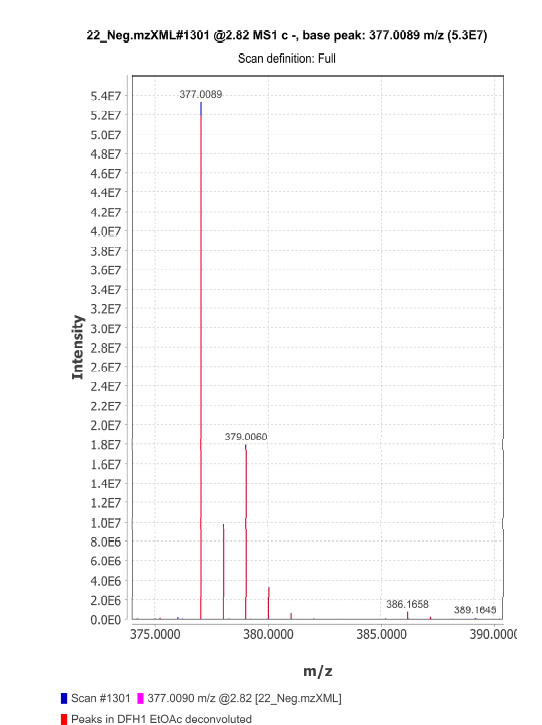


**Figure S27**. MS scan of ID523. The isotopic pattern of this scan supports the dereplication indicating that this feature corresponds to 5-Chlorodermorubin.

**
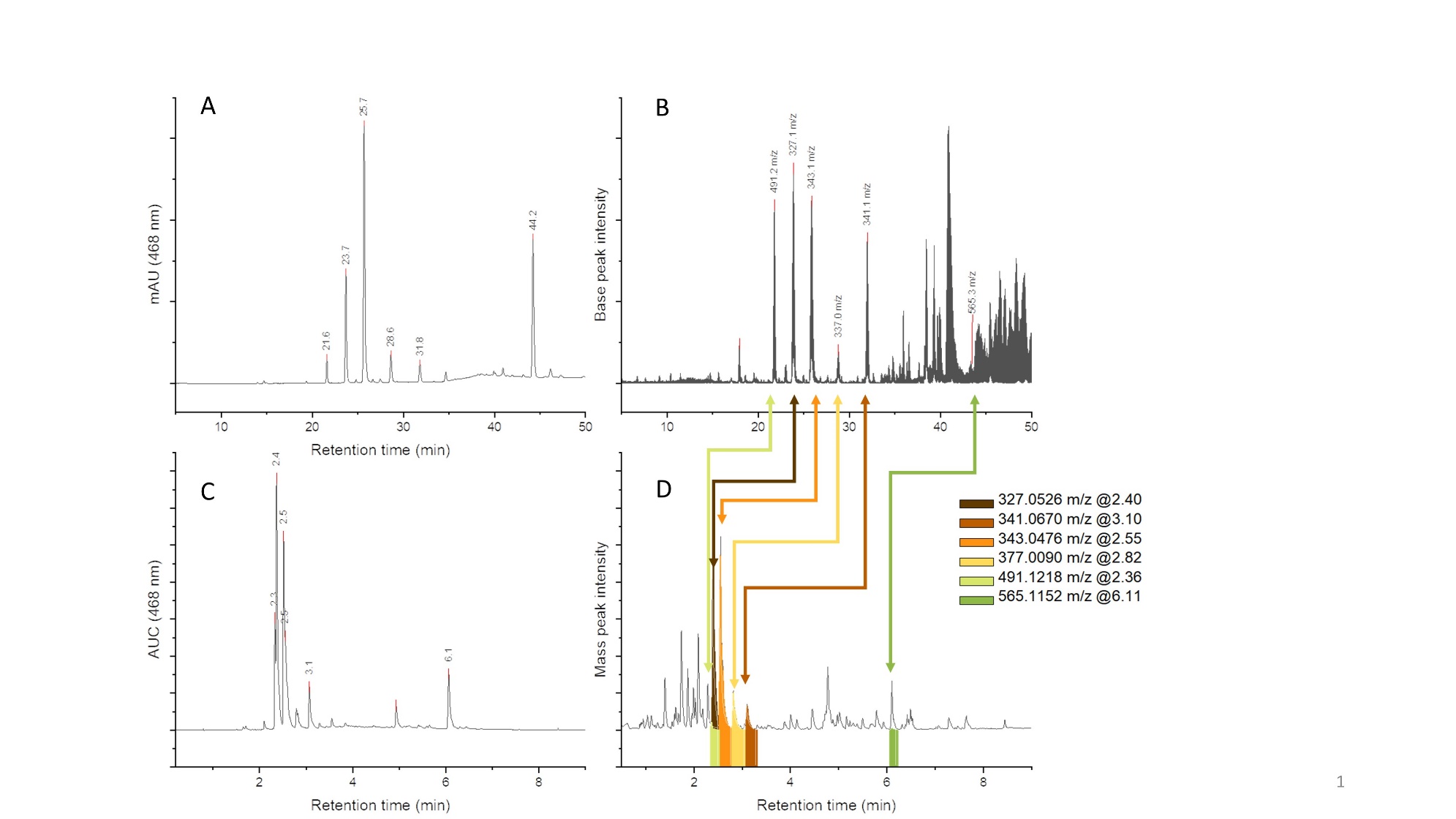
**

**Figure S28.** (A) Chromatogram (468 nm) of the HPLC-DAD-MS analysis of the ethyl acetate fraction from the methanolic extract (DFH1A_EtOAc). (B) The MS scan of the HPLC-DAD-MS analysis. (C) Chromatogram (468 nm) of the UHPLC-DAD-HRMS analysis of DFH1A_EtOAc. (D) The MS scan of the UHPLC-DAD-MS analysis. Highlighted by colors are the corresponding traces of the respective pigment-peaks (n = 6).

1. Photophysical and Photochemical Investigation
   1. Experimental Setup

The quantum yields of singlet oxygen generation and phosphorescence were determined in a custom-built setup described in detail previously.^12^ All optical parts were connected with optical fibres from Avantes, with a diameter of 600 μm. The sample, consisting of the compound in deuterated methanol (500 µL), was added to a semi-micro cuvette from fireflysci with 4 mm and 10 mm path lengths, and placed in a CUV-UV/VIS-TC temperature-controlled cuvette holder (Avantes). The sample was allowed to equilibrate at 20 °C. Emission spectroscopy was performed with a 450 nm fibre-coupled laser (LRD-0450, Laserglow), which was set to 50 mW or 15 mW at the cuvette (4 mm beam diameter; 0.4 W∙cm^−2^) at a 90° angle with respect to the spectrometer. The excitation power was measured using a S310C thermal sensor connected to a PM100USB power meter (Thorlabs). The emission spectra were recorded using two separate spectrometers for the UV-Vis and NIR emission, i.e. from 300 nm to 1000 nm for the emission (Avantes 2048L StarLine spectrometer) and from 1000 nm to 1700 nm for the phosphorescence of singlet oxygen (^1^Δ_g_) around 1275 nm (Avantes NIR256-1.7TEC spectrometer, detector set to −11 °C). The infrared emission spectrum was acquired within 9 seconds. Similarly, the visible emission spectrum was acquired within 2 seconds. UV-Vis absorption spectra before and after emission spectroscopy were measured using an Avalight-DHc halogen-deuterium lamp (Avantes) as light source (turned off during emission spectroscopy) and the before mentioned UV-Vis spectrometer as detector, both connected to the cuvette holder at a 180° angle. All spectra were recorded using Avasoft 8.5 software from Avantes and further processed using Microsoft Office Excel 2010 and Origin Pro 9.1 software.

The quantum yields of phosphorescence and singlet oxygen production was calculated using the relative method with [Ru(bpy)_3_]Cl_2_ (*Φ*_Δ_ = 0.73, *Φ*_P_ = 0.015 in MeOD)^12-14^ and Perinaphthenone (PN) (*Φ*_Δ_ = 0.98)^15^ as standard according to the equation (eq. 3):

$\Phi_{\mathrm{sam}}=\Phi_{\mathrm{std}}\times\frac{A_{\mathrm{std}}^{450}}{A_{\mathrm{sam}}^{450}}\times\frac{E_{\mathrm{sam}}}{E_{\mathrm{std}}}$ (eq. 3)

where *Φ* is the quantum yield, *A*^450^ is the absorbance at 450 nm (always kept between 0.100 and 0.093 for a 4 mm path length), *E* is the integrated emission peak of singlet oxygen at 1270 nm or the integrated compoundemission between 465 and 950 nm, and *sam* and *std* denote the sample and standard, respectively.

Emission lifetimes on the ns-time scale were recorded at room temperature with a LifeSpec-II from Edinburgh Instruments, with excitation source of 455 nm pulsed diode lasers and emission intensity recorded at 600 nm.

- 1. Near infrared spectra


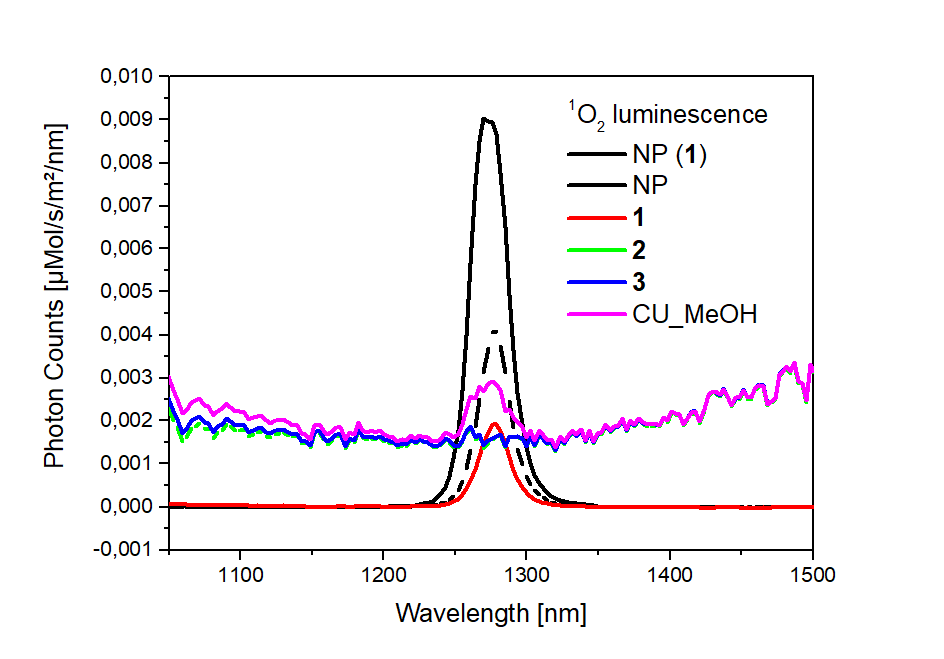

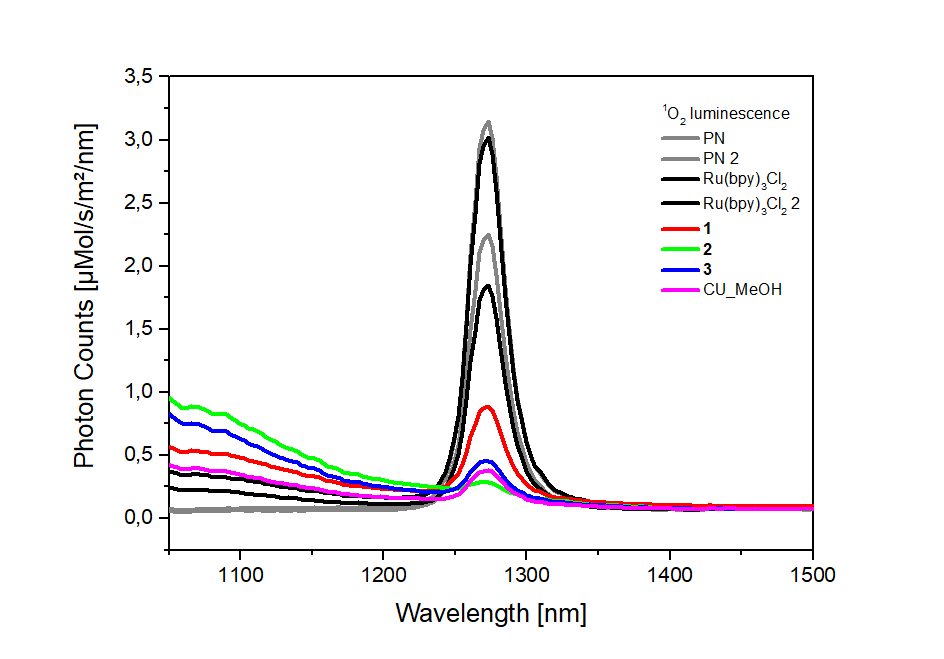
As visualized in Figure S29, irradiation of all isolated compounds and the methanol extract of *C.uliginosus* yields a band at 1270 nm indicating the production of ^1^O_2_.

PN

PN

**Figure S29.** Calibrated raw data of all measured ^1^O_2_ phosphorescence bands including the reference standards Perinaphthenone (PN) and Ru(bpy)_3_Cl_2_ for two series of measurements. Left MeOH, Right CHCl_3_. CU_MeOH = Methanol extract of *Cortinarius ulignosus*.

- 1. Emission spectra


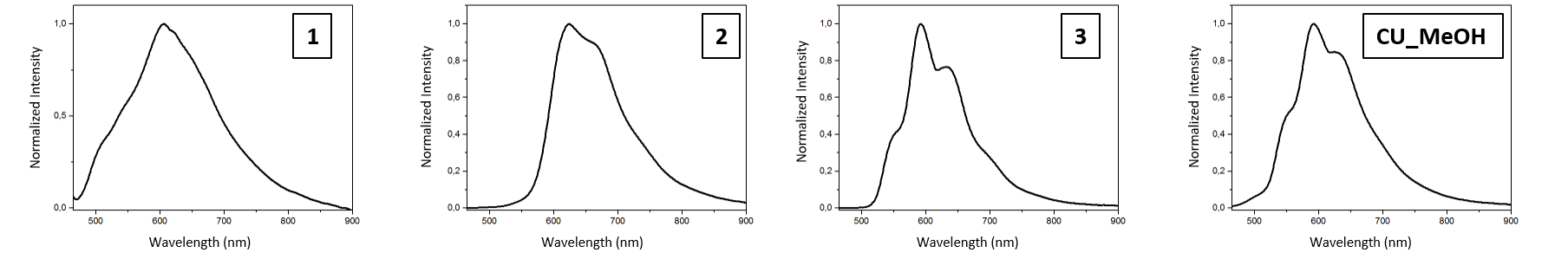


**Figure S30.** Normalized emission spectra of all compounds in MeOH.

- 1. Photostability assay – Instrumental setup

For the photostability assay, the absorption spectra of the compounds in 3 mL MeOH were recorded with a Cary 50 or Cary 60 spectrometer from Agilent using quartz cuvettes with 1x1 cm path lengths and a temperature-controlled sample holder (T = 20 °C). Irradiation of the sample solution occurred from the top of the open cuvette using an in-house fabricated adapter for 450 nm LEDs from Roithner Lasertechnik with an output intensity of 15.0 or 9.4 mW which translates to photon fluxes of 1.36 · 10^-7^ Einstein/s or 8.50 · 10^-8^ Einstein/s. More details of the setup are described in a previous study.^16^

1. Stability testing of **1**

The pronounced ability of **1** to produce singlet oxygen upon irradiation with light as well as the photo-instability of this compound require the defining of optimal storage conditions. Thus, following experiment was carried out:

Aliquots of a dimethylformamide solution of **1** (c = 0.105 mg/mL) were pipetted into four white-glass HPLC-vials and stored for seven days under different conditions. Vial #1 was stored at room temperature and under the influence of sunlight, vial #2 at room temperature and under the influence of ambient light, vial #3 at room temperature and under exclusion of light, and vial #4 in a fridge at 6 °C and under exclusion of light. After seven consecutive days the four solutions were submitted to HPLC-DAD- and HPLC-DAD-MS analysis. Additionally, another aliquot of the dimethylformamide solution (vial #5) was stored in a freezer at -35 °C for four days and submitted to HPLC-DAD-MS analysis. The equipment and the conditions for the chromatographic analyses are depicted in the table below (Table S6). The results of this experiment are depicted in the table at the end of chapter 4 (Table S7).

**Table S6.** The HPLC-systems, mobile phases, conditions, and the column used for the stability testing.

|  | HPLC-system | Mobile phase | Conditions | Column |
| --- | --- | --- | --- | --- |
| HPLC-DAD | Agilent Technologies 1200 Series | isocratic mode  A [H_2_O], B [ACN + 0.1 % FA]  0 min: A 10 %, B 90 %  20 min: A 10 %, B 90 % | flow rate: 0.5 mL/min  column temperature: 35 °C | Phenomenex Synergi MAX-RP 80 A 150x4.60 mm 4 micron |
| HPLC-DAD-MS | Agilent Technologies 1260 Infinity II | gradient mode  0 min: A 90 %, B 10 %  30 min: A 50 %, B 50 %  34 min: A 10 %, B 90 %  50 min: A 10 %, B 90 %  55 min: A 90 %, B 10 % |  |  |

The influence of sunlight (vial #1) for the duration of seven days showed with a reduction of 83.20 % relative to the original concentration of 0.104 mg/mL a distinct decrease. Ambient light (vial #2) exhibited a weaker influence on the stability of the compound than sunlight. A reduction of 33.65 % was measured for the solution stored under ambient light at room temperature. Storing the solution at room temperature under the exclusion of light (vial #3) amounted to 76.86 % of the original concentration. Therefore, it can be concluded that the exclusion of light is necessary to ensure the stability of the compound. By combining the absence of light with low temperatures (vials #4 and #5) the degradation of **1** could be lowered even further. Vial #4 (fridge: 6 °C) still contained 86.06 %, whereas in vial #5 (freezer: -35 °C) a concentration of 99.56 % was measured. Based on the results of this experiment, it can be deduced, that light (sunlight > ambient light) and high temperatures lead to the degradation of **1**. Consequently, excluding the influence of any light source as well as low temperatures are recommended when working with this compound. Provided that a dimethylformamide solution of **1** is stored in a freezer at -35 °C under the exclusion of light, stability can be guaranteed.

**Table S7.** Results of the stability testing of **1** dissolved in DMF (c = 0.104 mg/mL).

| Vial # | Light conditions | Temperature [°C] | Storage time [days] | Residual concentration of 1 (relative to the original concentration of 0.104 mg/mL) [%] |
| --- | --- | --- | --- | --- |
| 1 | Sunlight | 25 | 7 | 16.80 |
| 2 | Ambient light | 25 | 7 | 66.35 |
| 3 | Light exclusion | 25 | 7 | 76.86 |
| 4 | Light exclusion | 6 | 7 | 86.06 |
| 5 | Light exclusion | -35 | 7 | 99.56 |

- 1. Stability testing under cell culture conditions
  2. The influence of time and temperature

The stability of solutions of **1-3** in DMSO as well as the dilutions of those solutions in Opti-MEM® (2.5 % FCS, P/S) used for the (photo)cytotoxicity-assay were assessed.

For determining the stability of the compounds **1-3** while being solved in DMSO (concentrations are depicted in Table S8), the solutions were stored in brown-glass HPLC-vials and analysed via HPLC (gradient mode / parameters depicted in Table S6). The respective peak area of the first injection (detection wavelength λ = 468 nm) was set as 100 % and all following injections were compared with the initial area. For a duration of 33 hours and 19 minutes (room temperature, exclusion of light) the stability of the three compounds solved in DMSO could be confirmed as no significant decrease in the area under the curve was being observed. The results are depicted in the table below (Table S8). Even 240 hours after the first injection no significant decrease in the concentration of the three compounds solved in DMSO and stored as mentioned above was detectable.

**Table S8.** Results of the stability assessment of DMSO solutions of **1-3** stored in brown-glass HPLC-vials at room temperature and under the exclusion of light. The time of measurement is given in brackets behind t_1-8_. During a time-span of 33 hours and 19 minutes no distinct decrease in the concentration of the solutions was being observed.

| (-)-7,7´-Biphyscion (1) (DMSO, c = 0.017 mg/mL, 30 µM) | | | | | | | | |
| --- | --- | --- | --- | --- | --- | --- | --- | --- |
|  | t_1_ | t_2_ (13h 19min) | t_3_ (16h 39 min) | t_4_ (19h 59 min) | t_5_ (23h 19min) | t_6_ (26h 39min) | t_7_ (29h 59min) | t_8_ (33h 19min) |
| Area [mAU*s] | 479.50 | 481.87 | 480.31 | 481.93 | 483.30 | 489.66 | 482.36 | 473.89 |
| Area% [relative to t1 (100 %)] | 100.00 | 100.49 | 100.17 | 100.51 | 100.79 | 102.12 | 100.60 | 98.83 |
| Dermolutein (2) (DMSO, c = 0.219 mg/mL, 667 µM) | | | | | | | | |
|  | t_1_ | t_2_ | t_3_ | t_4_ | t_5_ | t_6_ | t_7_ | t_8_ |
| Area [mAU*s] | 2093.19 | 2140.91 | 2082.62 | 2100.75 | 2076.50 | 2082.95 | 2076.98 | 2139.32 |
| Area% [relative to t1 (100 %)] | 100.00 | 102.28 | 99.49 | 100.36 | 99.20 | 99.51 | 99.23 | 102.20 |
| Dermorubin (3) (DMSO, c = 0.230 mg/mL, 668 µM) | | | | | | | | |
|  | t_1_ | t_2_ | t_3_ | t_4_ | t_5_ | t_6_ | t_7_ | t_8_ |
| Area [mAU*s] | 2708.55 | 2733.51 | 2832.44 | 2738.24 | 2782.28 | 2751.43 | 2689.83 | 2762.91 |
| Area% [relative to t1 (100 %)] | 100.00 | 100.92 | 104.57 | 101.10 | 102.72 | 101.58 | 99.31 | 102.01 |

To assure the stability of the tested compounds under cell culture conditions, dilutions of the aforementioned DMSO solutions of **1-3** in Opti-MEM® were analyzed via HPLC prior to and after an incubation period of 24 hours at 37°C. Comparison of the respective peak areas before and after the incubation period showed that for **1**,**2**, and **3** a possible degradation process is neglectable. The results of the conducted experiment are shown in Table S9.

**Table S9.** Results of the stability testing of **1-3** under cell culture conditions (prior to and after an incubation period of 24h at 37 °C). No significant decrease in concentration was being observed for the dilutions of **1-3** which are being used in the (photo)cytototxicity-assay.

|  | Dilution | Concentration (µM) | Area (t0) [mAU*s] | Area (t1 / 24h later) [mAU*s] | Residual area (relative to t0) [%] |
| --- | --- | --- | --- | --- | --- |
| (-)-7,7´-Biphyscion (1) | High (initial) | 8.83 | 131.22 | 125.67 | 95.77 |
|  | 3+1 | 2.21 | 36.42 | 35.98 | 98.77 |
|  | 3.5+0.5 | 1.10 | no signal detectable | | |
| Dermolutein (2) | High (initial) | 100.00 | 291.93 | 286.65 | 98.19 |
|  | 3+1 | 12.50 | 72.60 | 72.18 | 99.42 |
|  | 3.5+0.5 | 6.25 | 35.32 | 35.29 | 99.91 |
| Dermorubin (3) | 3+1 | 12.50 | 92.22 | 85.58 | 92.80 |
|  | 3.5+0.5 | 6.25 | 48.06 | 47.38 | 98.60 |
|  | 3.8+0.2 | 2.50 | 17.94 | 17.78 | 99.07 |

- 1. The influence of blue light irradiation on the stability of **1**

The influence of blue light irradiation (468 nm, 9.3 J/cm^2^) on the stability of **1** dissolved in PBS and DMEM® was investigated. First, a stock solution of **1** was prepared in DMSO and diluted with PBS or DMEM, yielding solutions with concentrations of 24.36 µM. Aliquots were kept in the dark or irradiated with blue light (7.5 min, 9.3 J/cm^2^). Subsequently, the solutions were analyzed via HPLC (gradient mode / parameters depicted in Table S6). The peak with a retention time of 44.04 min (**1**) was integrated (detection wavelength = 440 nm) and the area of **1** in the irradiated solution was calculated relative to the area of **1** in the solution which was kept in the dark. After 7.5 min of blue light irradiation the area of **1** was decreased by 52.28 % in DMEM and 65.41 % in PBS. The results showed that **1** is sensitive to light and disintegrates into products lacking a relevant absorbance at 440 nm. The chromatograms and results are depicted in Figure S31.


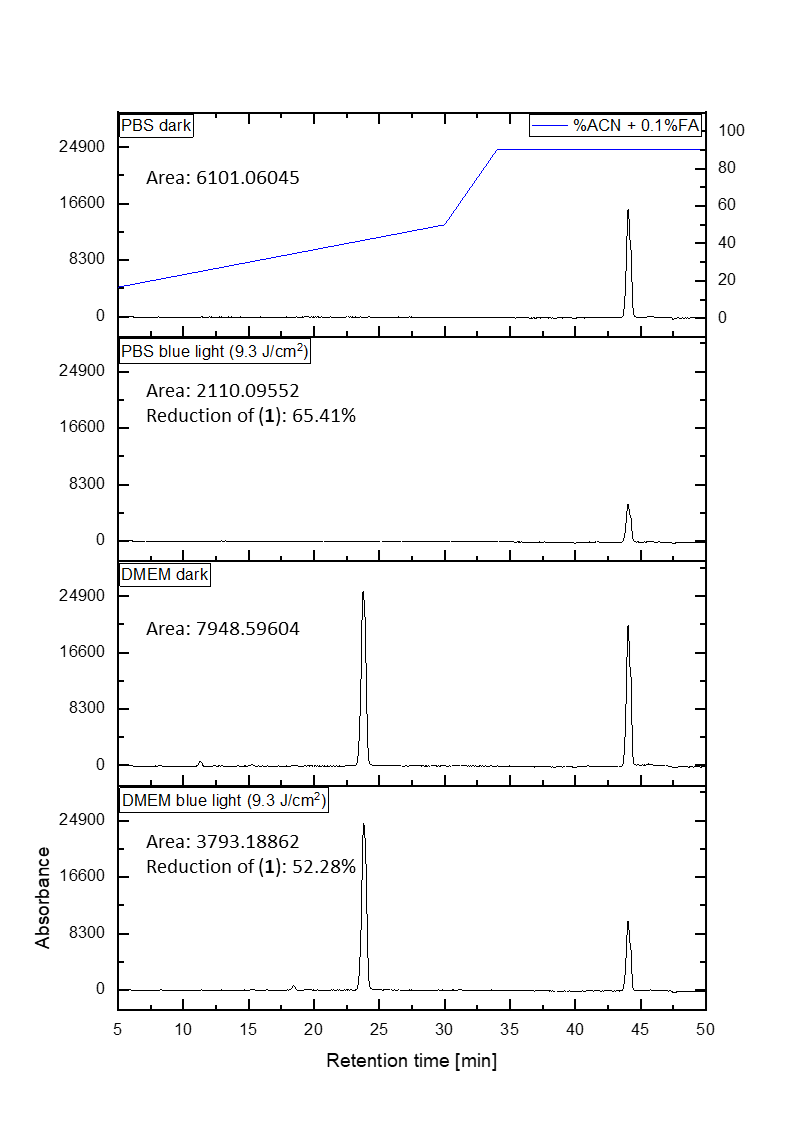


**Figure S31.** HPLC chromatograms (detection wavelength = 440 nm) and results of the investigation regarding the stability of **1** in PBS and DMEM under blue light irradiation (7.5 min, 9.3 J/cm^2^). A strong decrease (> 50 %: PBS and DMEM) in the concentration of **1** in was observed after irradiation.

- - 1. The influence of blue light irradiation on the stability of **1** dissolved in OPTI-MEM® (cell culture conditions)

The influence of blue light irradiation (468 nm, 9.3 & 18.6 J/cm^2^) on the stability of (**1**) solved in Opti-MEM® was investigated. First, a stock solution of **1** was prepared in DMSO and diluted with Opit-MEM®, yielding a solution with a concentration of 24.36 µM. An aliquot was kept in the dark or irradiated with blue light (7.5 min, 9.3 J/cm^2^ | 15 min, 18.6 J/cm^2^), respectively. Subsequently, the solutions were analyzed via HPLC using a Phenomenex Synergi MAX-RP 80 A 150x4.60 mm 4 micron column. The liquid-chromatographic investigation was performed in gradient mode: (A: H2O, B: ACN + 0.1% FA) 0 min – 10 % B, 3 min – 50 % B, 5 min – 90 % B, 7 min – 99 %, 11 min – 99 %, 11.01 min – 10 %, 15 min – 10 %, and flow rate, injection volume, column temperature and detection wavelength were set to 1 mL/min, 10 µL, 40 °C, respectively. The peak with a retention time of 10.22 min **1** was integrated with Origin 2020 and the area of **1** in the non-irradiated solution (dark) was defined as 100 %. Irradiating the solution for 7.5 min with blue light (468 nm) yielded a reduction of **1** of 6.1 %. An irradiation duration of 15 min reduced the concentration of **1** by 10.9 %. The chromatograms and results are depicted in Figure S32.


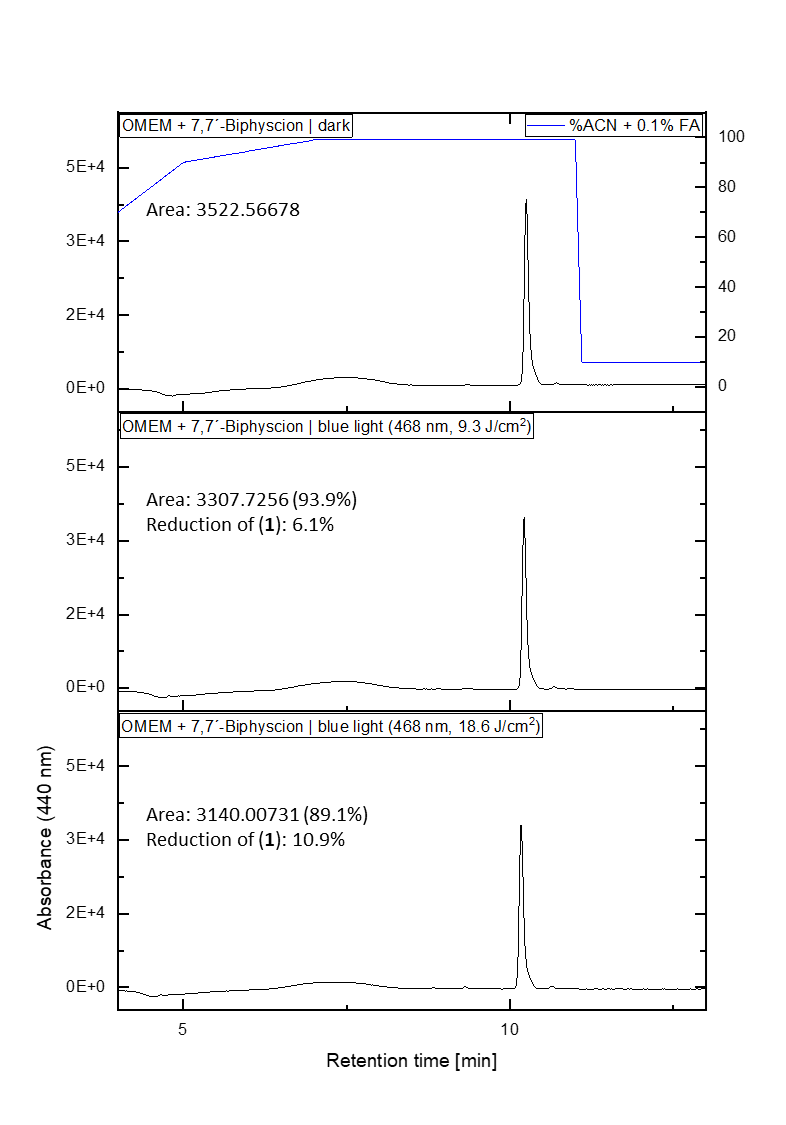


**Figure S32.** HPLC chromatograms (detection wavelength = 440 nm) and results of the investigation regarding the stability of **1** in Opti-MEM® under blue light irradiation (468 nm: 7.5 min, 9.3 J/cm^2^ | 15 min 18.6 J/cm^2^). A decrease in the concentration of **1** was observed after irradiation (7.5 min: - 6.1 %, 15 min: - 10.9 %).

1. Biological testing
   1. (Photo)cytotoxicity-Assay and Cell Culture Maintenance

Investigating the photocytotoxicity of extracts and pure compounds was done as published elsewhere.^1^ Cell culture maintenance was performed according to standard procedures. The complete experimental protocol is included in the main manuscript. In Figure S 33 the irradiation instrument is depicted.


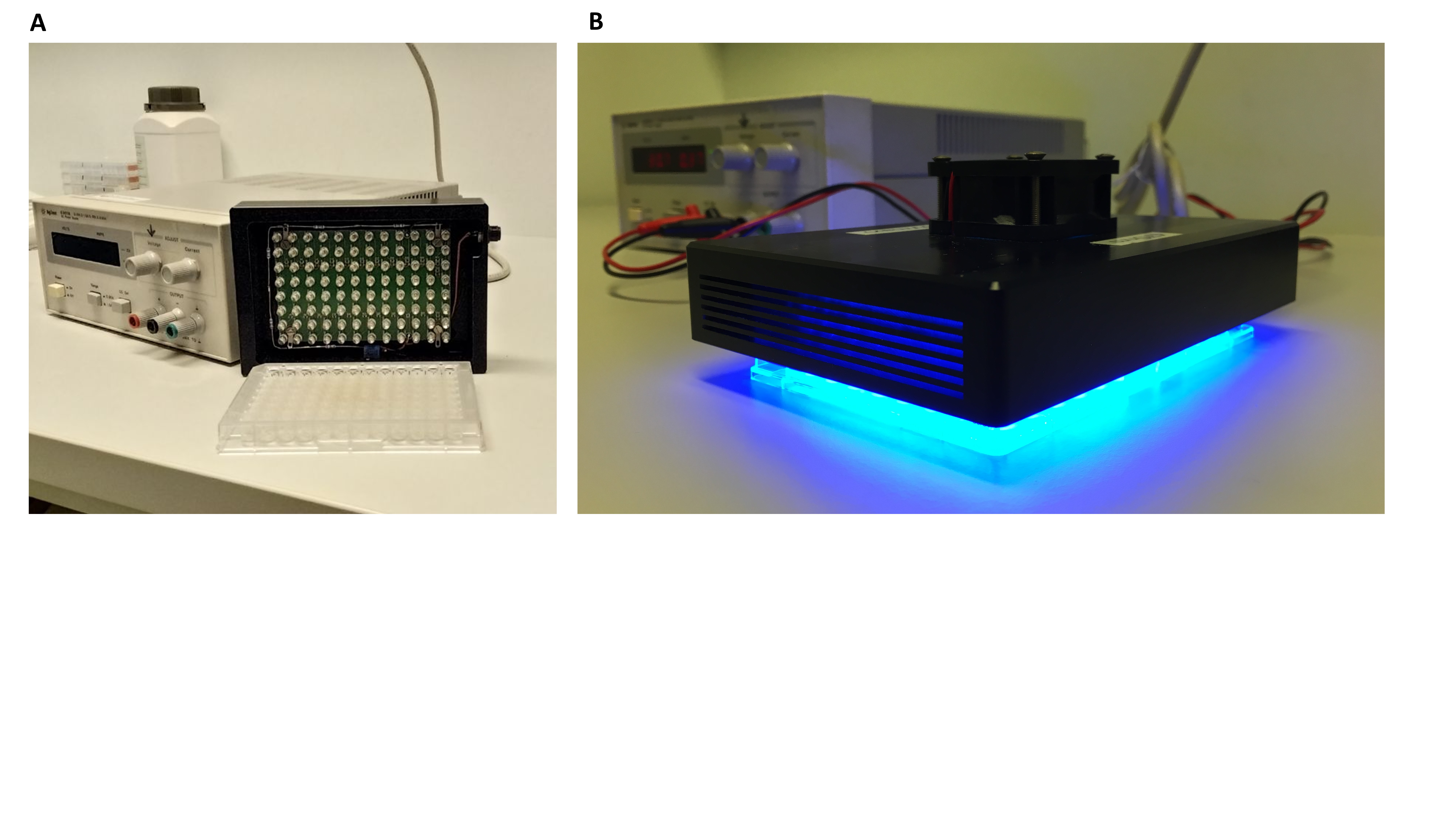


Figure S 33. Irradiation setup used for the cell irradiation experiments. A) "Offline" modus showing the 96 LEDs insight the lid. B) Irradiation setup in its active modus illuminating a 96 well plate.

- 1. (Photo)toxicity results of the extracts

All extracts were tested against A549 and HeLa cells. The results are presented in Table S10. The apolar extracts are generally characterized by a weak selectivity (S.I. max. 19), whereas the polar extracts hold a potentially higher selectivity: All MeOH extracts were non-toxic in the dark up to a concentration of 50 µg/mL.

**Table S10.** The results of the (photo)cytotoxicity evaluation of the seven investigated *Cortinarius* species. EC_50_ values (µg/mL) are provided including the upper and lower limits of the confidence interval (95 %) presented in grey. The irradiation was done employing blue light (λ = 468 nm). Different radiant exposures were applied, as listed in the table.

| EC_50_ |  | A549  (Dark) | | A549  (3.1 J/cm²) | | A549 (6.2 J/cm²) | | A549  (9.3 J/cm²) | | HeLa  (Dark) | | HeLa  (3.1 J/cm²) | |
| --- | --- | --- | --- | --- | --- | --- | --- | --- | --- | --- | --- | --- | --- |
| *C. uliginosus* | PE | 28 | 2 | 25 | 3 | 12 | 7 | 12 | 6 | 27 | 2 | 24 | 2 |
|  |  |  | 2 |  | 3 |  | 3 |  | 4 |  | 2 |  | 2 |
|  | MeOH | >50 | | >50 | | 19 | 5 | 7 | 1 | >50 | | >50 | |
|  |  |  |  |  |  |  | 4 |  | 1 |  |  |  |  |
| *C. phoeniceus* | PE | 28 | 2 | 26 | 2 | 19 | 5 | 18 | 5 | 27 | 2 | 25 | 2 |
|  |  |  | 2 |  | 2 |  | 4 |  | 4 |  | 1 |  | 2 |
|  | MeOH | >50 | | >50 | | >50 | | >50 | | >50 | | >50 | |
| *C. semisanguineus* | PE | 35 | 3 | 18 | 5 | 9 | 1 | 9 | 3 | 28 | 2 | 14 | 1 |
|  |  |  | 3 |  | 4 |  | 1 |  | 2 |  | 2 |  | 1 |
|  | MeOH | >50 | | >50 | | >50 | | >50 | | >50 | | >50 | |
| *C. cinnabarinus* | PE | 28 | 4 | 9 | 2 | 10 | 2 | 9 | 2 | 16 | 4 | 8 | 1 |
|  |  |  | 3 |  | 2 |  | 2 |  | 2 |  | 3 |  | 1 |
|  | MeOH | >50 | | >50 | | >50 | | >50 | | >50 | | >50 | |
| *C. olivaceofuscus* | PE | 28 | 3 | 9 | 3 | 7 | 1 | 6 | 1 | 26 | 2 | 9 | 1 |
|  |  |  | 3 |  | 2 |  | 1 |  | 1 |  | 1 |  | 1 |
|  | MeOH | >50 | | >50 | | 20 | 7 | 7 | 2 | >50 | | >50 | |
|  |  |  |  |  |  |  | 5 |  | 1 |  |  |  |  |
| *C. cinnamomeoluteus* | PE | 37 | 4 | 6 | 1 | 4 | 1 | 2 | 2 | 32 | 3 | 8,1 | 2 |
|  |  |  | 4 |  | 1 |  | 1 |  | 1 |  | 3 |  | 2 |
|  | MeOH | >50 | | >50 | | 6 | 1 | 2 | 1 | >50 |  | >50 |  |
|  |  |  |  |  |  |  | 1 |  | 1 |  |  |  |  |
| *C. malicorius* | PE | 38 | 41 | n.d | | n.d. | | 6 | 3 | n.d. | | n.d. | |
|  |  |  | 20 |  |  |  |  |  | 2 |  |  |  |  |
|  | MeOH | >50 |  | n.d |  | n.d. |  | 45 | 46 | n.d |  | n.d. |  |
|  |  |  |  |  |  |  |  |  | 23 |  |  |  |  |

n.d. not determined in this study

- 1. (Photo)toxicity results of **1-3**

Solutions of **1-3** were tested against A549, AGS, and T24 cells. Neither **2** nor **3** showed a (photo)cytotoxic effect on the three cell lines. The results for **1** are presented in the main manuscript.

- 1. Uptake studies

For this experiment, cells of the lung cancer cell line A549 were seeded (100.000 cells/well – three 6-well plates) in Opti-MEM® (2.5 % FCS, P/S) and after 24h treated with either medium (**CTR**), a fraction containing a mixture of **2** and **3** (**Dermo**, 3.37 µg/mL), which originated from the mycochemical analysis of *Cortinarius rubrophyllus*, or a fraction containing a high concentration of **1** as well as **2** and **3** (**EtOAc**, 50 µg/mL), which resulted from the liquid-liquid extraction of the methanolic *Cortinarius uliginosus* extract with ethyl acetate. Out of the twelve available wells, four wells were used for the treatment with **CTR**, **Dermo**, or **EtOAc** respectively. After additional 24h the supernatants were removed. The respective treatment solutions (i.e. **CTR**, **Dermo**, **EtOAc**) of two 6-well plates were pooled and stored in 15 mL falcon tubes. The wells of one 6-well plate were supplied with fresh Opti-MEM and the plate was irradiated with blue light (468 nm, 9.3 J/cm^2^). After 48h the light induced effect was investigated via microscopy. The results of the microscopic analysis are depicted below (Figure S34, **B**). The pooled treatment solutions of the first two 6-well plates were lyophilized and then extracted via sonication (1x 5 mL acetone, 1 x 3 mL methanol). After 5 minutes of sonication each, the solutions were centrifuged, the supernatants were combined and the extracts were dried. The extracts were solved in 400 µL of DMSO, filtered through cotton wool and stored in brown-glass HPLC vials until further analysis. The cells were washed with 2 mL DMEM (10 % FCS, P/S) and two times with 2 mL PBS. Subsequently the cells were trypsinized with 500 µL Trypsin-EDTA (0.25 %) and suspended in additional 2 mL of DMEM. Again, cells belonging to the same treatment group were pooled, centrifuged, and the supernatants were removed. The cells were extracted and the extracts prepared for liquid chromatographic analysis as described above. HPLC-DAD analysis was carried out using a Phenomenex Synergi MAX-RP 80 A 150x4.60 mm 4 micron column in gradient mode (A: H_2_O, B: ACN + 0.1 % FA – 0 min 10 % B, 20 min 65 % B, 25 min 65 % B, 35 min 99 % B, 40 min 99 % B, 40.10 10 % B, 45 min 10 % B) and flow rate, injection volume, column temperature, and detection wavelength were set to 0.6 mL/min, 5 µL, 40 °C, and 455 nm, respectively. The results of this investigation can be seen in Figure S34 (A).


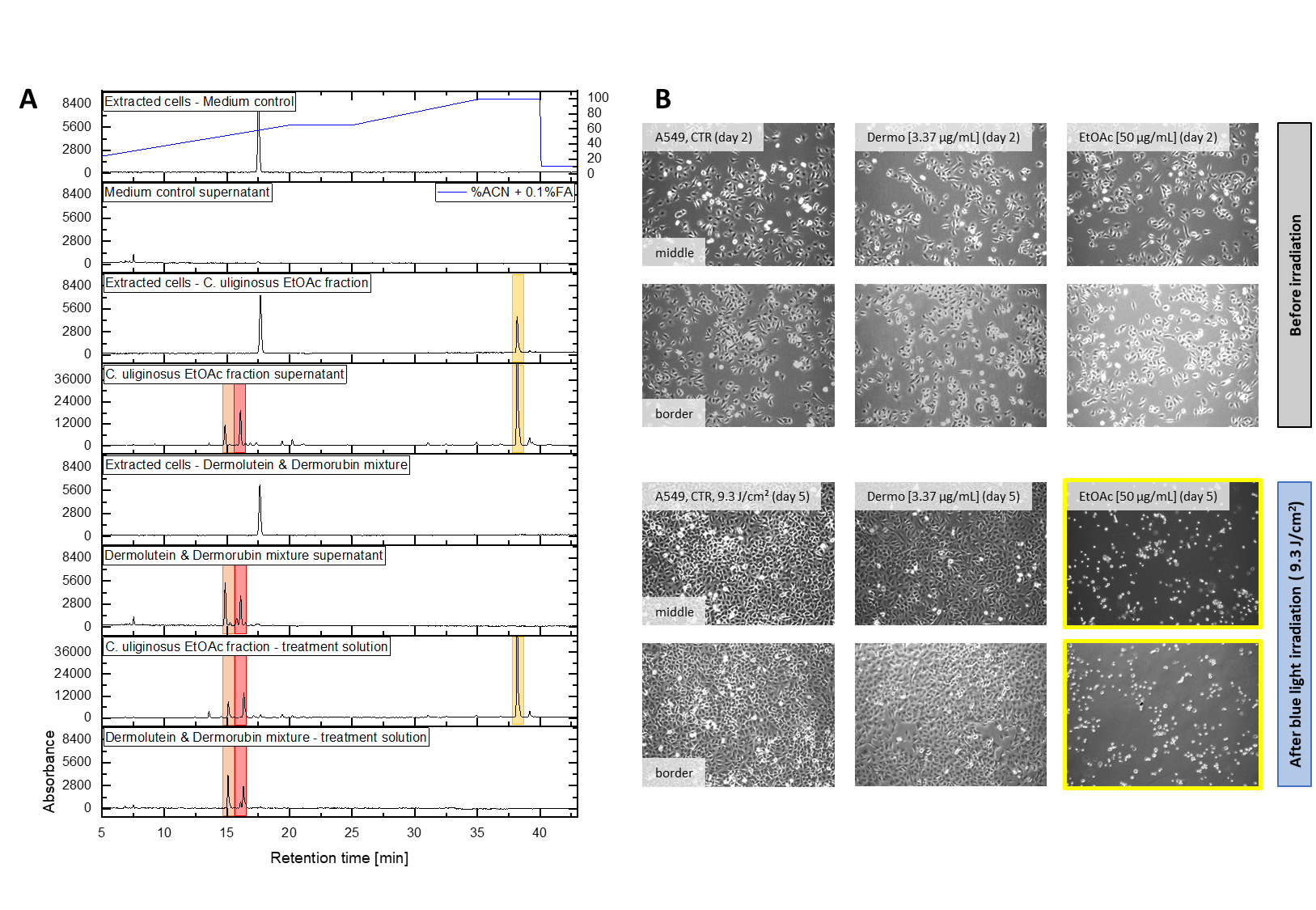


**Figure S34.** Results of the anthraquinone uptake studies via the combination of cell culture experiments and HPLC-DAD analysis. **A**) HPLC chromatograms (detection wavelength = 455 nm) of the extracts of A549 cells treated with **CTR**, **Dermo**, and **EtOAc** as well as the respective supernatants and treatment solutions. The peaks of interest are highlighted in different colors: dermolutein (**2**) is marked in orange (RT = 15.10 min), dermorubin (**3**) in red (RT = 16.34 min), and (-)-7,7´-biphyscion (**1**) in yellow (RT = 38.18 min). This study revealed that out of the three anthraquinones of interest just **1** is absorbed by A549 cells in a quantitative manner and was therefore detectable via HPLC-DAD. The uptake of **2** and **3** was not observable in our experimental setup. **B**) Micrographs of the A549 lung cancer cell line (100x magnification). Pictures were taken from the middle of the wells and from their borders as indicated in the figure via "middle" and "border" given in grey boxes. The upper two rows show the cells before irradiation (day 2 = 24h after seeding) and the two lower rows depict the A549 cells after blue light irradiation (468 nm, 9.3 J/cm^2^) (day 5 = 96h after seeding). Irradiation of the A549 cells treated with **EtOAc** (i.e. a fraction containing a high concentration of **1**, highlighted with a yellow box) resulted in cell death.

- - 1. Uptake of 1

In order to describe ratio of **1** absorbed by the cancer cells versus **1** remaining in the treatment solution supernatant (**EtOAc**) the respective solutions (chapter 5.4) were analyzed via HPLC-DAD. The parameters were chosen as depicted in chapter 5.4. The area of **1** was determined by integrating the peak at a retention time of 38.18 min (detection wavelength = 455 nm) with Origin 2020. The factor describing the absorbed amount of **1** was calculated as follows: Area **1** in the extracted cells / Area **1** in the supernatant. The chromatograms and the result of the calculation can be seen in Figure S35. The calculation resulted in a factor of 0.06.


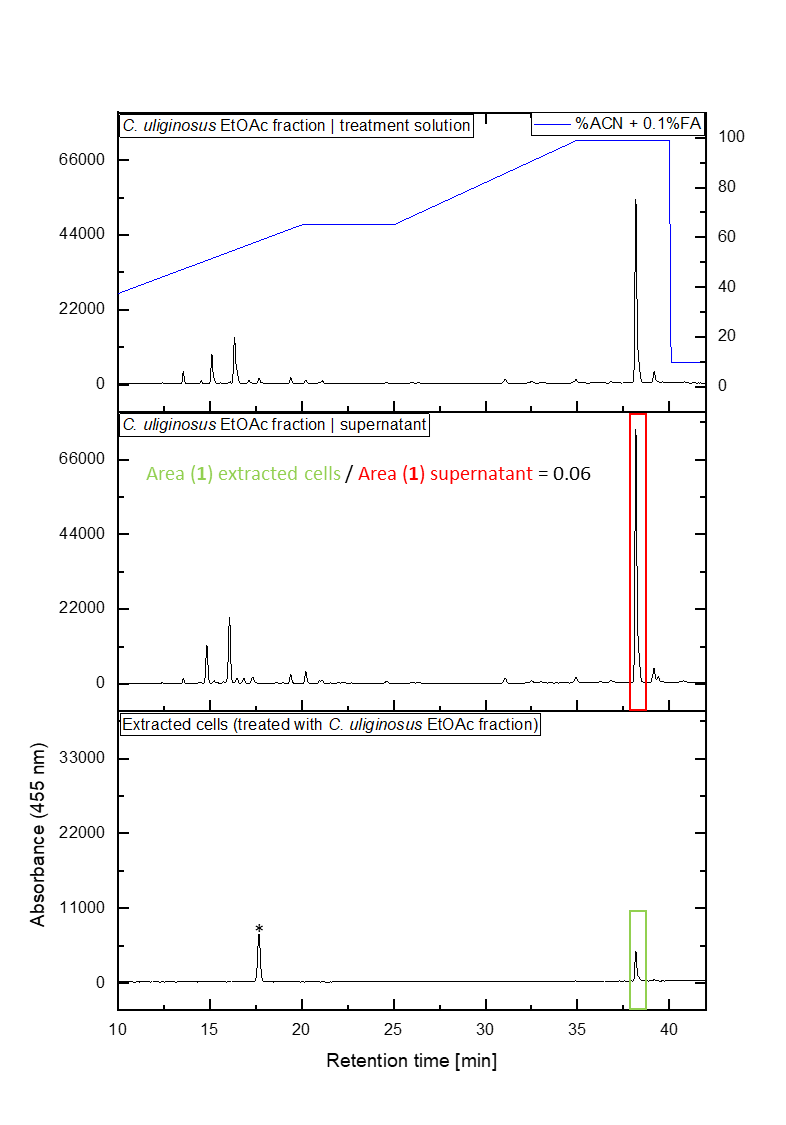


**Figure S35.** HPLC-chromatograms of the **EtOAc** treatment solution, the **EtOAc** supernatant, and the extracted A549 cells treated with **EtOAc** (detection wavelength = 455 nm). The peak representing (**1**) is highlighted with a red/green colored box. The peak indicated with a * at RT = 17.66 min was identified as phenol red. A factor describing the absorbed amount of (**1**) was calculated as area of **1** in the extracted cells divided by the area of **1** remaining in the supernatant. The calculation resulted in a factor of 0.06.

- - 1. Anthraquinone uptake studies: conclusions

The HPLC-DAD analysis showed that treating A549 cells with a mixture of dermolutein (**2**) and dermorubin (**3**) (**Dermo**) didn't result in a cellular uptake as no peaks were observed. Additionally, an irradiation experiment revealed that the treatment with **Dermo** didn't induce cell death. Although **2** and **3** are capable of generating singlet oxygen upon blue light irradiation, they are not able to kill cancer cells in a light dependent manner. It can be concluded that this is due to a lacking cellular uptake.
However, extracting **1** from the harvested cells was possible as it can be seen in the HPLC-DAD experiment (Figure S34, Figure S35). The blue light-induced cell death observed for cells treated with **EtOAc** can be clearly attributed to the effect of **1**.
To sum up, **2** and **3** don't show signs of photocytotoxicity, because of a hampered uptake whereas **1** is quantitatively absorbed by the cancer cells, resulting in the ability to kill them light-dependent.

- 1. Metabolic Activity assay

The metabolic activity of A549 cancer cells treated with **1** was tested employing the resazurin assay or so-called Alamar-assay^17^. Resazurin is reduced to resorufin by NADPH or NADH in normal functioning mitochondria. Impairment of the mitochondrial function or of the cell itself denies such reductions, thus indicating mitochondrial dysfunction.

Cells resuspended in Opti-MEM (complete) were seeded into 96 well plates (2000 cells/well). After 24h, cells were treated with **1** (concentration series ranging from 10 nM to 2 µM), staurosporine (5µM), H_2_O_2_ (1 %), and DMSO (0.5 %). After additional 24h, the medium was replaced by fresh Opti-MEM complete media prior to irradiation (468 nm, 9.3 J/cm²). The measurement, according to the manufacturers' protocol, was done 48h after irradiation. In brief, the medium was replaced by medium containing resazurin (30 µg/mL). The reduction of resazurin to resorufin was measured as increase in fluorescence (λ_exc_ = 540 nm/ λ_em_ = 590 nm). The results (**Figure S36**) are in line with the results obtained via the SRB assay and prove the harmlessness of **1** in the dark. Solely under irradiation, the metabolic activity of the cells is impaired.


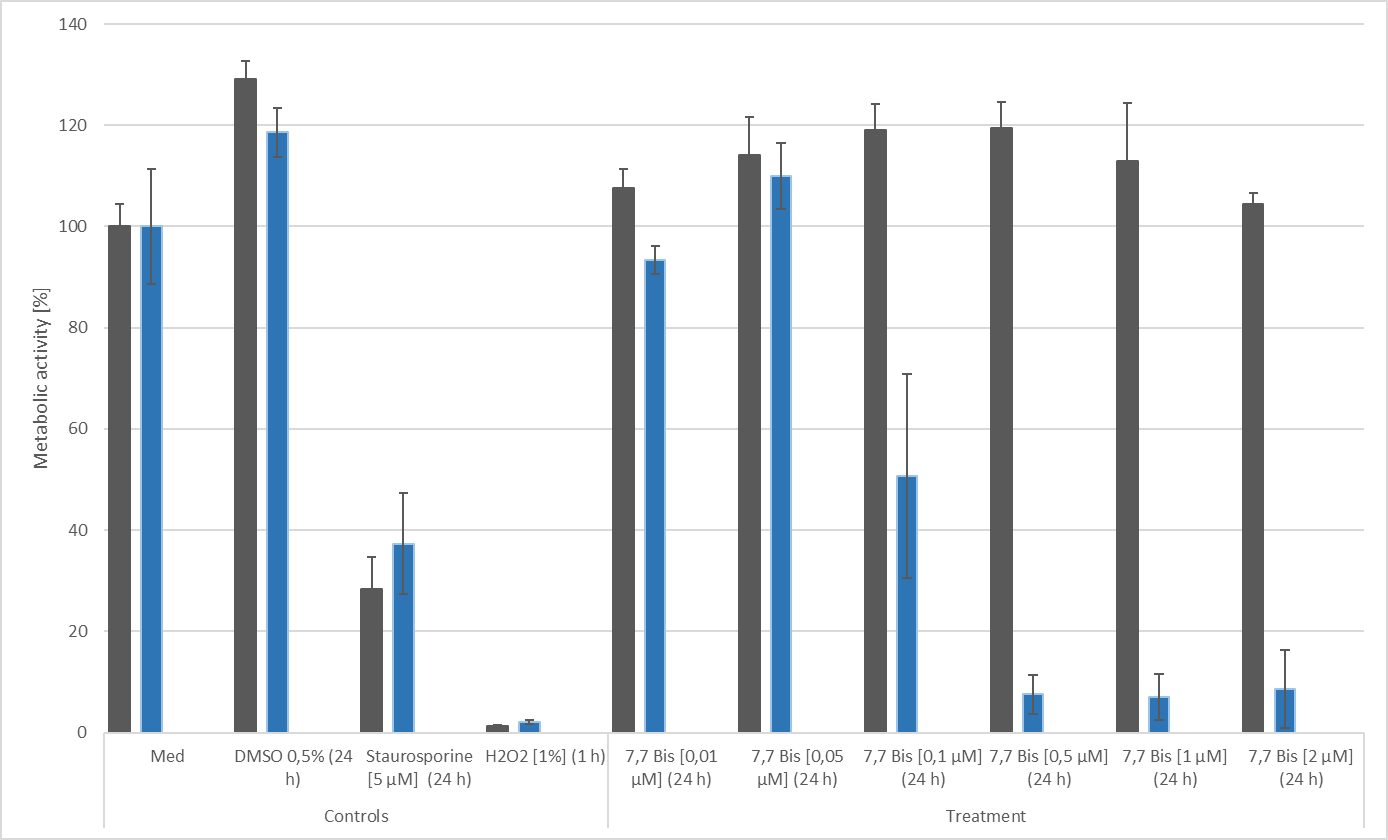


**Figure S36.** Results of the metabolic activity assay of A549 cells treated in the dark (black bars) or under irradiation (blue bars, 468 nm, 9.3 J/cm²).

- 1. Cell Cycle Analysis

Cells treated as for the metabolic activity assay (see 5.4) were submitted to a cell cycle analysis 24h after light irradiation. In brief, the supernatant was removed and EDTA (200 µL, 10 mM) directly added and incubated at 37°C for 10-15 minutes). After this incubation time, the cells rounded up and easily can be easily detached by gently shaking the plate. In a next step, the staining buffer (300 µL, MEM medium supplemented with FBS (10 %), saponin (0.1 %), propidium iodide (3.3 µg/ml), and RNAse A (5µg/mL)) was added and the mixture incubated under constant shaking in the dark and at room temperature for 30 minutes. Afterwards, the cells were directly submitted to a flow cytometric analysis (Guava® easyCyte 8HT Benchtop Flow Cytometer (Merck Millipore)).

The data processing was done utilizing FCS Express 7 (De Novo Software) as previously described.^18^ Shortly, the forward scatter (FSC-HLin) was plotted against the side scatter (SSC-HLin) and the cell population gated. Aggregates of cells, potentially caused by the ethanol fixation, were excluded by plotting the signal height against the signal area of the red-fluorescence (633nm) channel and gating single cells. Finally, a DNA-histogram fit analysis was performed with FCS-Express utilizing the method of Dean and Jett.^19^

The results (**Figure S37**) show that **1** has no effect on the cell proliferation of A549 cancer cells, neither in the dark nor under blue light irradiation. Thus, indicating that **1** is harmless in the dark and that the selectivity caused by light irradiation compensates the non-existing selectivity between non-malignant and malignant cells.


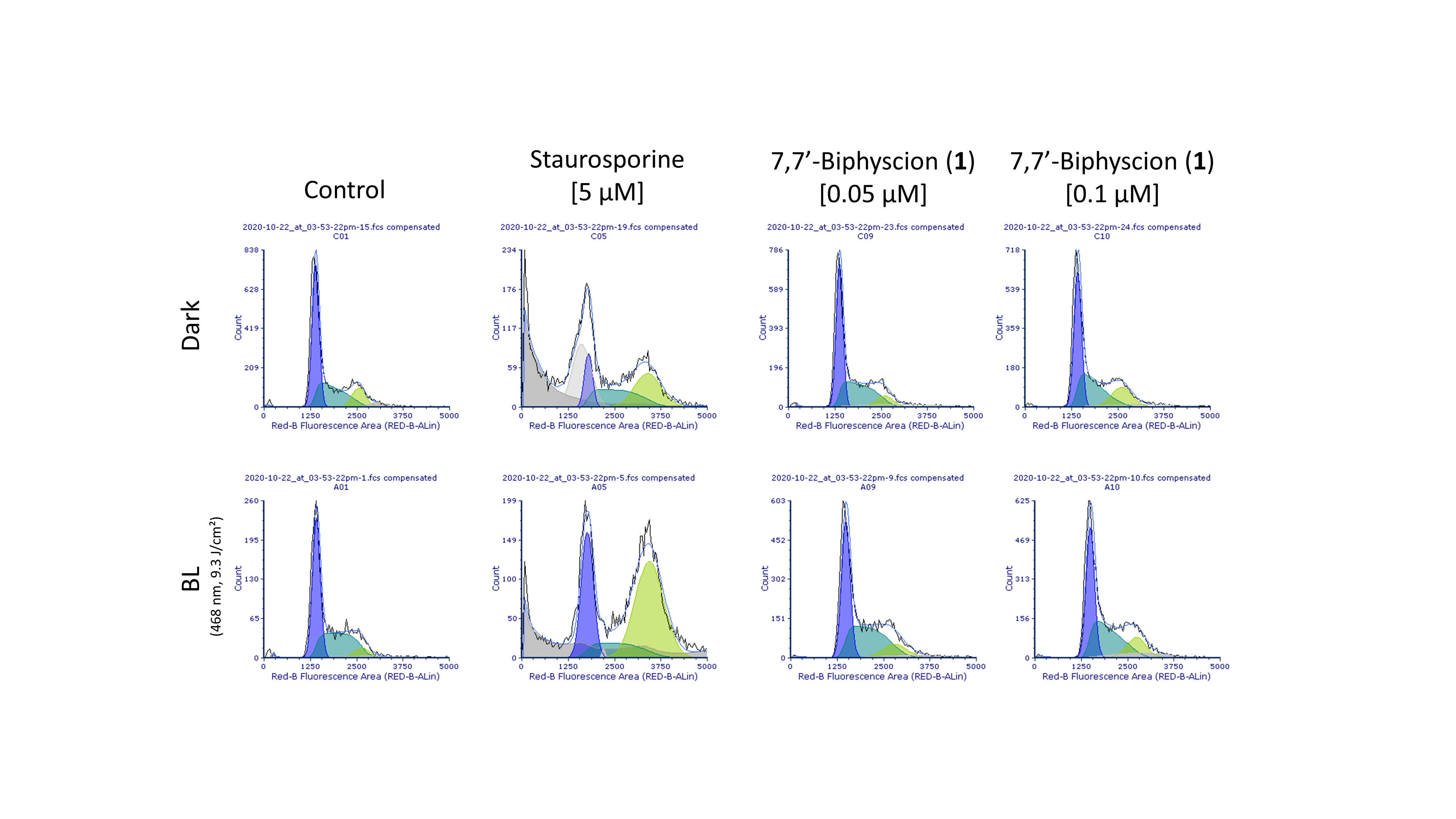
**Figure S37.** Cell cycle analysis (A549 cells) treated with **1**. While staurosporine effects the cell cycle, **1** showed no effect at the chosen concentrations.

- 1. Cell viability assay

A cell viability assay was performed utilizing Annexin V, an Annexin V binding buffer, and DRAQ7 from Becton Dickinson (BD) Austria, Wien. In brief, 2.4E5 cells of the cancer cell line A549 were seeded in six well cells and treated with an adjusted concentration of **1**, which resulted in a similar effective concentration per cell. 24h after treatment the cell media was replaced by fresh medium and the cells were subsequently irradiated. After additional 24h of incubation the viability assay was performed according to the manufacturers' protocol. In brief, the supernatant was collected, and the cells were washed with cold PBS (w/ Ca^2+^). After removing the PBS (which was combined with the supernatant) the cells were trypsinated at 37°C for 3-5 min. Afterwards, the cells were suspended, cold PBS added, and the suspension combined with the supernatant and both centrifuged (500g, 5 min). After removing the supernatant, the cell pellet was resuspended in PBS, the cells counted, and again centrifuged. After removing the PBS, approx. 2E6 cells were resuspended in binding buffer (500 µL) and an aliquot (100 µL) thereof transferred into a fresh reaction tube. After adding the staining mix (10 µL, annexin V/DRAQ7 1:1) the cells were gently shaken and incubated in the dark at room temperature for 15 minutes. The reaction was quenched by adding binding buffer (400 µL) and the stained cells were submitted to flow cytometric analysis (Guava® easyCyte 8HT Benchtop Flow Cytometer (Merck Millipore, Darmstadt, Germany)). The processing of the obtained raw data was done utilizing FCS express 7 (De Novo Software). The scatter plots and numeric analysis of the utilized controls are displayed in **Figure S38** and **Figure S40**. **Figure S39** and **Figure S41** show the analysis of **1**. The results show that **1** induces concentration dependent apoptotic cell death in A549 cells under irradiation. Under dark control conditions, however, no sign of cell death was observed, thus underlining the harmlessness of **1** in the dark.


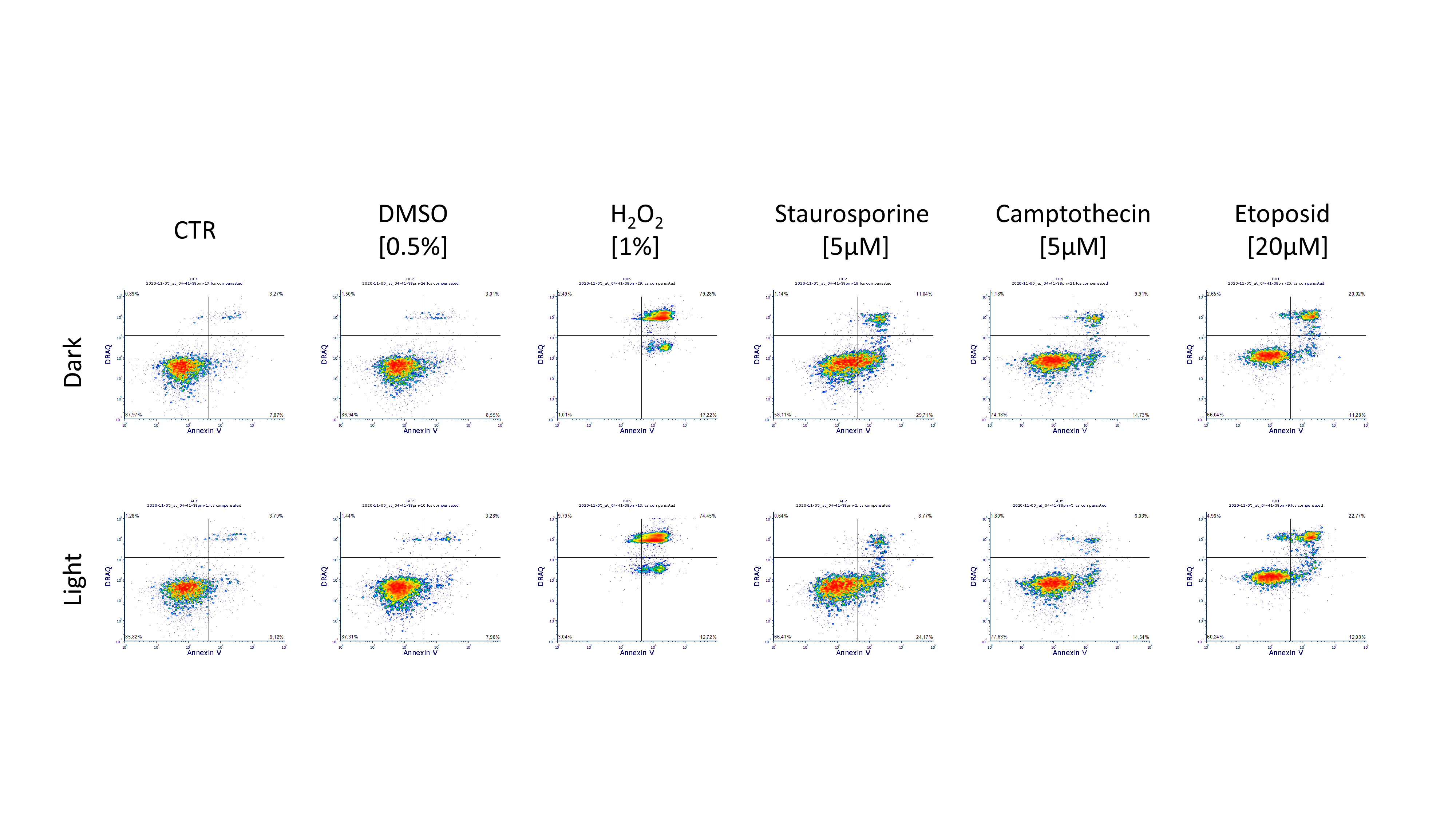


**Figure S38.** Density plots of the viability assay (A549 cells) showing on the x-axis the annexin V binding behavior and on the y-axis the DRAQ staining. An increasing population in the lower right quadrant indicates apoptotic cell death, while an increase in the upper left population indicates necrotic cell death. Displayed are all utilized controls under dark and irradiation conditions (468 nm, 9.3 J/cm²).


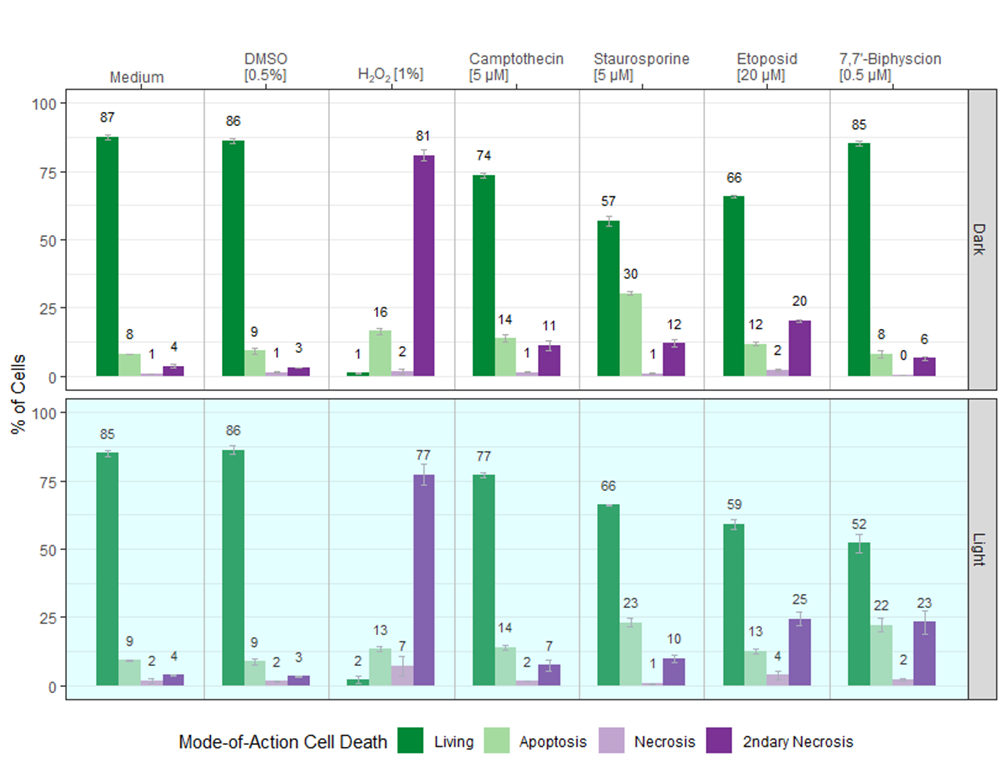


**Figure S39.** Distribution of the by the utilized controls and **1** induced cell death types. Error bars indicate standard errors obtained from up to four replicates.


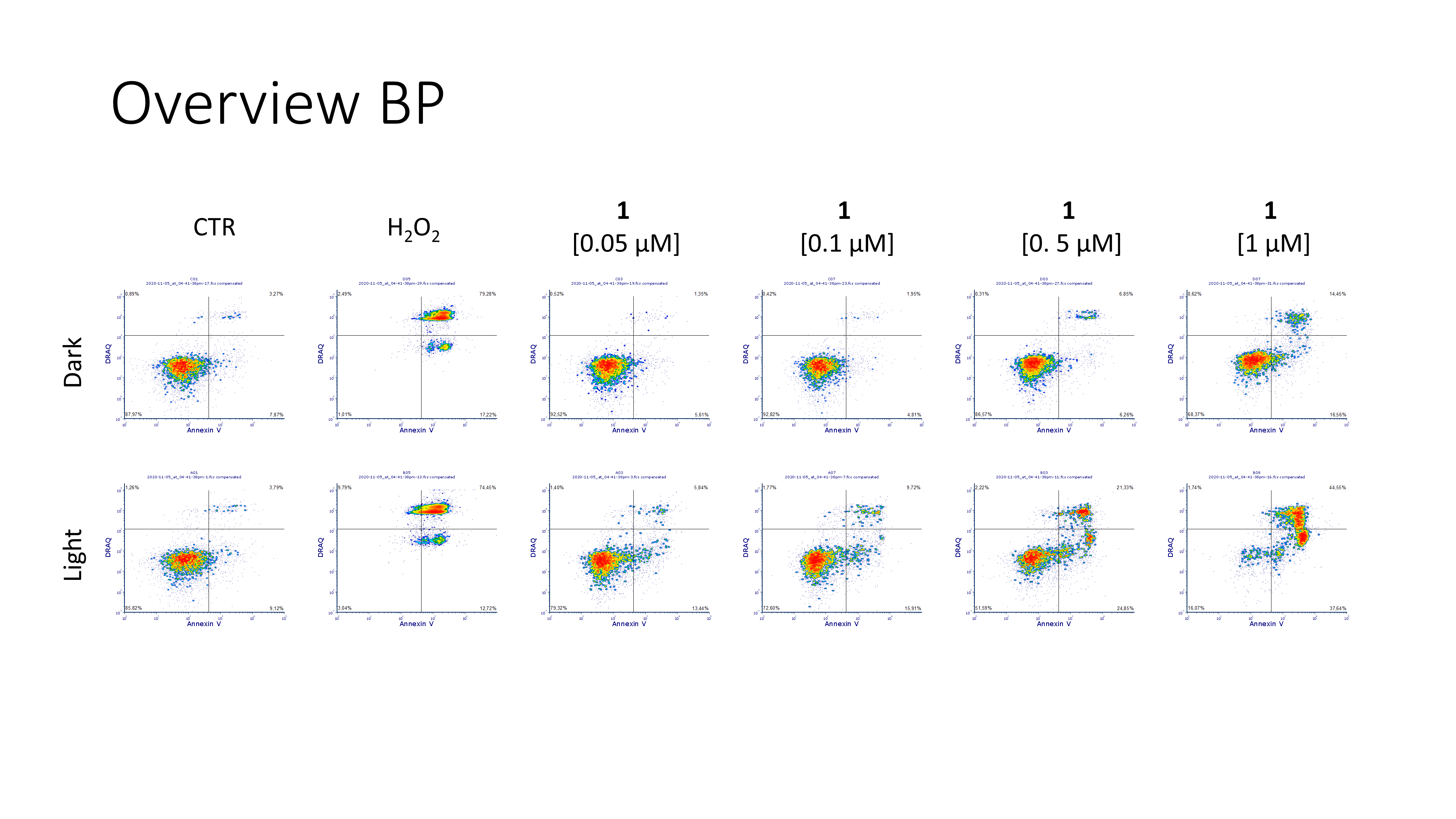


**Figure S40.** Density plots of the viability assay (A549 cells) showing on the x-axis the annexin V binding behavior and on the y-axis the DRAQ staining. An increasing population in the lower right quadrant indicates apoptotic cell death, while an increase in the upper left population indicates necrotic cell death. Displayed are a concentration series of **1** and hydrogen peroxide under dark and irradiation conditions (468 nm, 9.3 J/cm²).


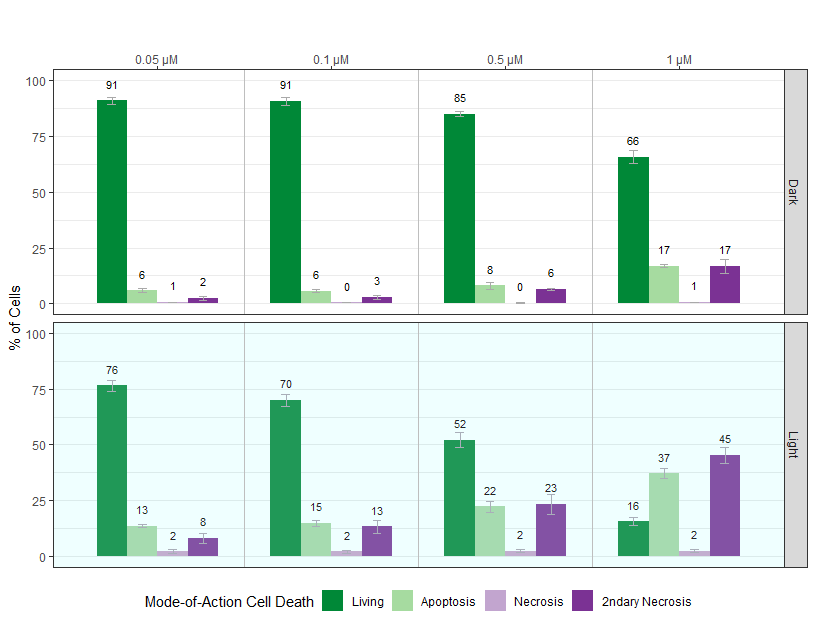


**Figure S41.** Distribution of the in A549 cells induced types of cell death by **1**. Error bars indicate standard errors obtained from up to four replicates.

- 1. Photocytotoxicity Inhibition Studies

The ability of different antioxidants to chemically quench singlet oxygen (DMA studies) as well as their protective capabilities against photodamage induced by **1** on A549 and T24 cancer cells were assessed. Following well-known radical scavengers were chosen for this experiment: L-cysteine, N-acetyl-L-cysteine, L-histidine, sodium azide, L-ascorbic acid,
and β-carotene.^20-24^

- - 1. Singlet oxygen quenching (DMA studies)

The radical scavenging abilities of the aforementioned antioxidants (chapter 5.8) were assessed with the help of the singlet oxygen probe 9,10-dimethylanthracene (DMA). In the presence of ^1^O_2_, DMA selectively reacts to the endoperoxide, exhibiting a decrease in absorbance at 377 nm. Radical scavengers inhibit this decrease in absorbance (e.g. L-ascorbic acid used in our DMA-assay^3^). In this experiment the four photosensitizers (PSs) – curcumin, emodin, rose bengal, **1** – were used.

The photosensitizers were dissolved in DMSO (0.1 mg/mL). Stock solutions of L-cysteine, N-acetyl-L-cysteine, L-histidine, sodium azide, and β-carotene were prepared in HPLC-grade water or DMSO according to their optimal solubility. Subsequently, the solutions were diluted with HPLC-grade ethanol (96 % v/v) and mixed with an ethanolic DMA solution (final concentration of DMA: 0.3325 mM). 190 µL of the respective dilutions were pipetted into a 96-well plate and mixed with 10 µL of photosensitizer (PS) solution. The final concentrations of the antioxidants are given in Table S11. Then, the plates were irradiated with blue light (λ = 468 nm, 2 x 5 min, 12.4 J/cm^2^; curcumin, emodin, and **1**) or green light (λ = 519 nm, 2 x 4.6 min, 8.46 J/cm^2^; rose bengal). The absorbance at 377 nm was measured at three time points (BL: 0 min, 5 min, 10 min; GL: 0 min, 4.6 min, 9.2 min) with a plate reader and the difference between the third and the first time point was calculated. The absorbance difference of the PS solutions in the presence of antioxidants was calculated relative to the difference of the PS solutions alone. L-Cysteine and L-histidine were not soluble in this experimental setup and therefore failed to yield interpretable results. N-Acetyl-L-cysteine, sodium azide, and β-carotene were able to quench singlet oxygen and therefore inhibit the reduction of absorbance to a variable extent. The exact results are depicted in the table below (Table S11).

**Table S11.** The results of the singlet oxygen quenching studies. N-Acetyl-L-cysteine, sodium azide, and β-carotene were able to inhibit the reduction of absorbance at 377 nm. Therefore, those antioxidants were capable of reducing the photoactivity of four different photosensitizers to a variable extent. Sodium azide (final concentration 50 mM) proved to be the most efficient quencher of singlet oxygen (reduction of photoactivity > 75 %).

|  |  | Reduction of photoactivity [%] | | | |
| --- | --- | --- | --- | --- | --- |
| Antioxidant | Final concentration in the well | Curcumin | Emodin | (-)-7,7´-Biphyscion (**1**) | Rose bengal |
| N-Acetyl-L-cysteine | 30 mM | 10.69 ± 2.59 | 33.51 ± 1.45 | 24.58 ± 2.20 | 37.49 ± 2.62 |
| Sodium azide | 50 mM | 98.52 ± 0.80 | 95.82 ± 0.74 | 76.17 ± 0.77 | 86.43 ± 0.93 |
| β-Carotene | 50 µM | 68.38 ± 3.76 | 42.48 ± 5.45 | 38.55 ± 3.55 | 6.41 ± 8.04 |

- - 1. Inhibition of (-)-7,7´-biphyscion (1) photodamage

As depicted in chapter 5.1, A549 and T24 cells were seeded (2000 cells/well) in Opti-MEM® and were treated with **1** (final concentrations: 0.5, 0.25, and 0.125 µM) after 24 hours. Two hours prior to aspiration of the medium (one hour: sodium azide), the cells were treated with solutions of different antioxidants (i.e., N-acetyl-L-cysteine (10 mM), L-cysteine (10 mM), L-ascorbic acid (100 µM), sodium azide (50 mM), L-histidine (50 mM), and β-carotene (50 µM)) in Opti-MEM®/DMSO (99:1). After the medium was exchanged by fresh Opti-MEM®, the cells were irradiated with blue light (λ = 468 nm, 7.5 min, 9.3 J/cm^2^). Thereafter, the cells were fixed, stained, and the absorbance at 540 nm was measured as described in chapter 5.1.
Out of the investigated antioxidants neither exhibited cytotoxicity on both cell lines in the tested concentrations, with the exception of sodium azide. Furthermore, the antioxidant solutions failed to diminish photodamage induced by **1** in the concentration range 0.5-0.25 µM. Solely N-acetyl-L-cysteine (NAC) and β-carotene (BC) were capable of reducing the photocytotoxicity resulting from a 0.125 µM solution of **1** in both cancer cell lines: NAC – 52 % (T24), 14 % (A549); BC – 38 % (T24), 19 % (A549). The results of the experiment using the 0.125 µM solution of **1** are depicted below in Figure S42.


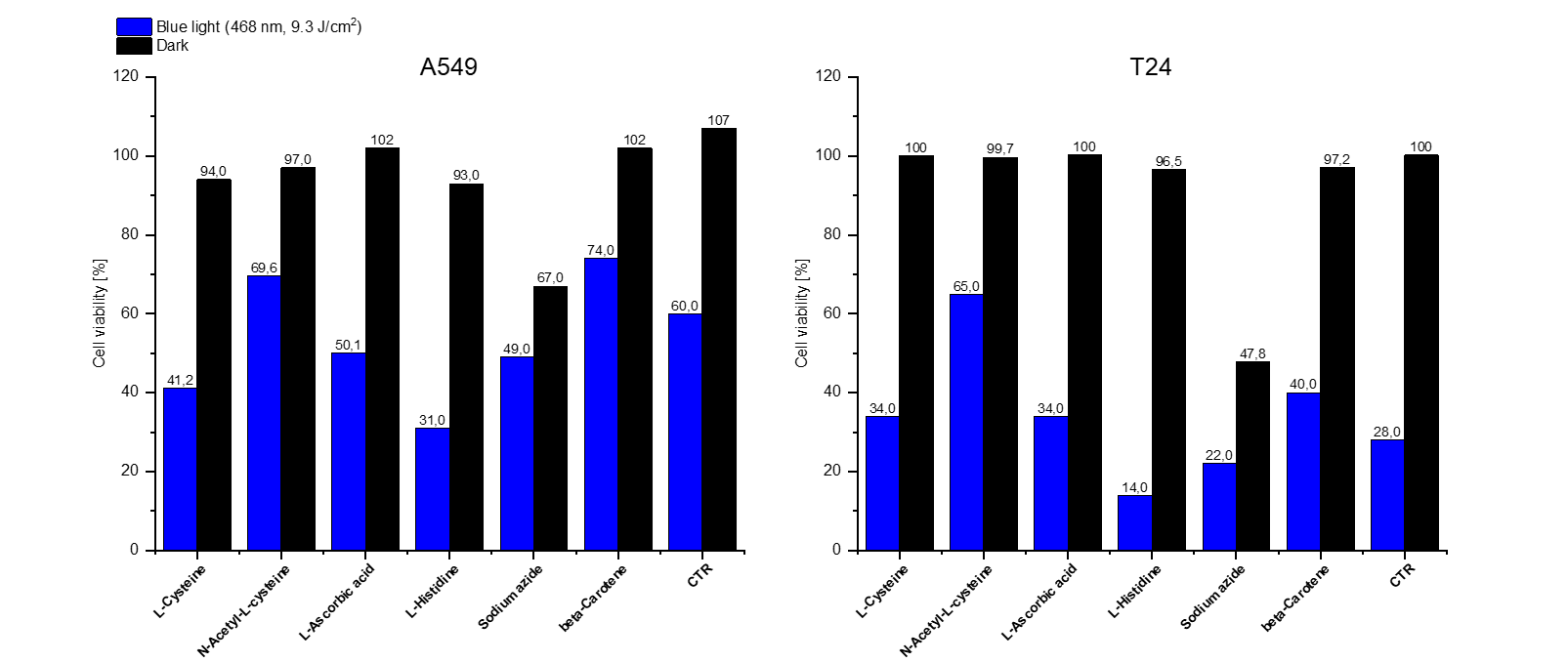


**Figure S42.** The photocytotoxicity-inhibiting potential of different antioxidants. The radical scavengers L-cysteine (10 mM), N-acetyl-L-cysteine (10 mM), L-ascorbic acid (100 µM), L-histidine (50 mM), sodium azide (50 mM), and β-Carotene (50 µM) were tested for their ability to diminish photodamage on the cancer cell lines A549 and T24 resulting from a 0.125 µM solution of **1** (= CTR), which was irradiated for 7.5 min with blue light (468 nm / 9.3 J/cm^2^). The results are given as cell viability in percent.

- - 1. Photocytotoxicity studies: conclusions

N-Acetyl-L-cysteine and β-carotene not only showed the ability to chemically quench singlet oxygen generated by various photosensitizers (DMA studies), but were also capable of reducing **1** photodamage (0.125 µM) by up to 52 % in a cell-based assay without exhibiting cytotoxicity on their own.

- 1. Micrographs of treated cells.


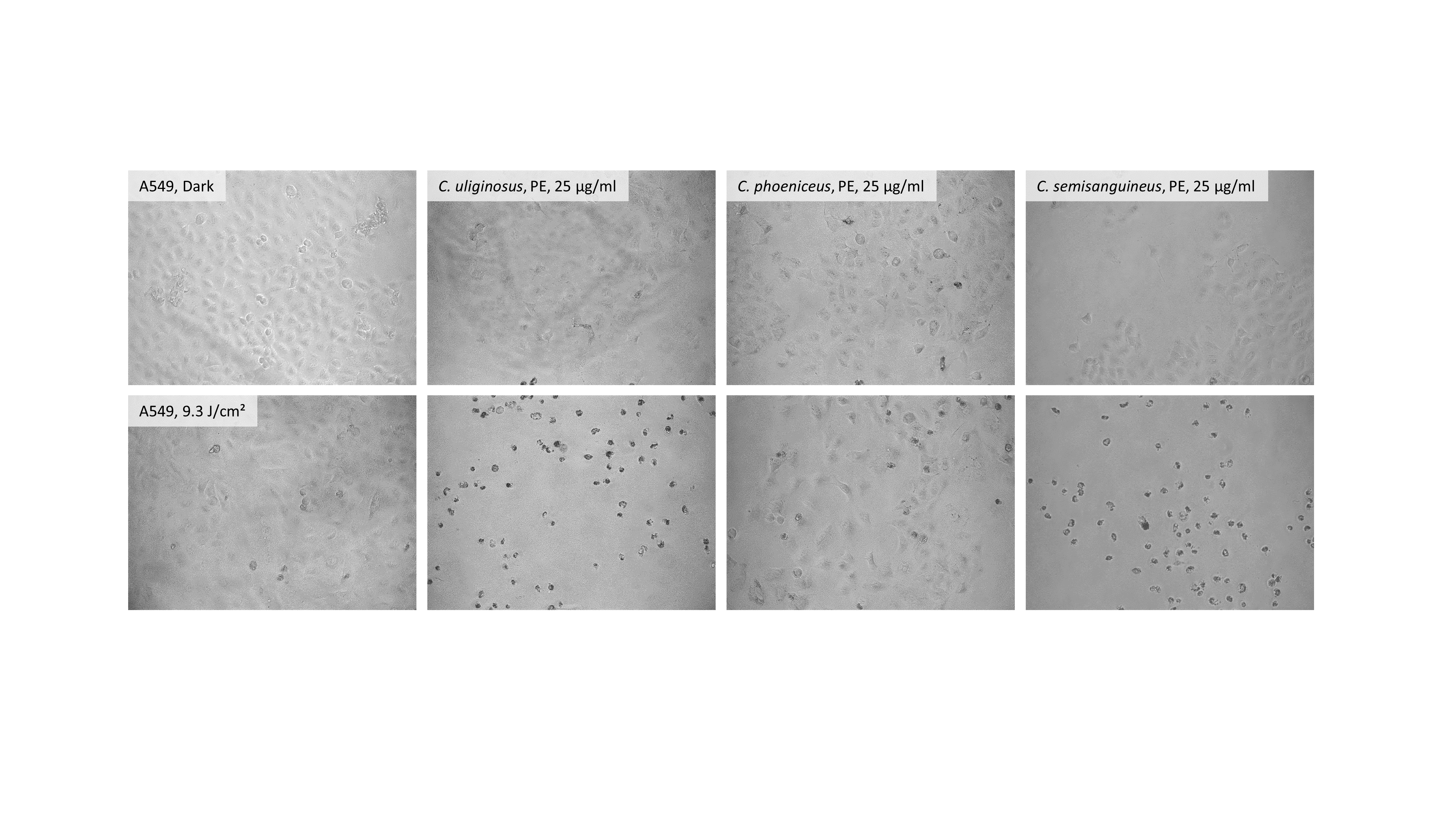


**Figure S43.** Micrographs (200x magnification) of cells of the lung cancer cell line A549 treated (24h) with the apolar extracts (25 µg/mL) of *C. uliginosus*, *C. phoeniceus,* and *C. semisanguineus.* The upper line of pictures shows treated cells in the dark, the lower line after irradiation with blue light (468 nm, 9.3 J/cm²). The shrunken cells observed for cells treated with *C. uliginosus* and *C. semisanguineus* and irradiated indicate that a programmed cell death was induced while the reduced cell density of cells treated in the dark with e.g. *C. phoeniceus* implicates a cell proliferation inhibiting effect.


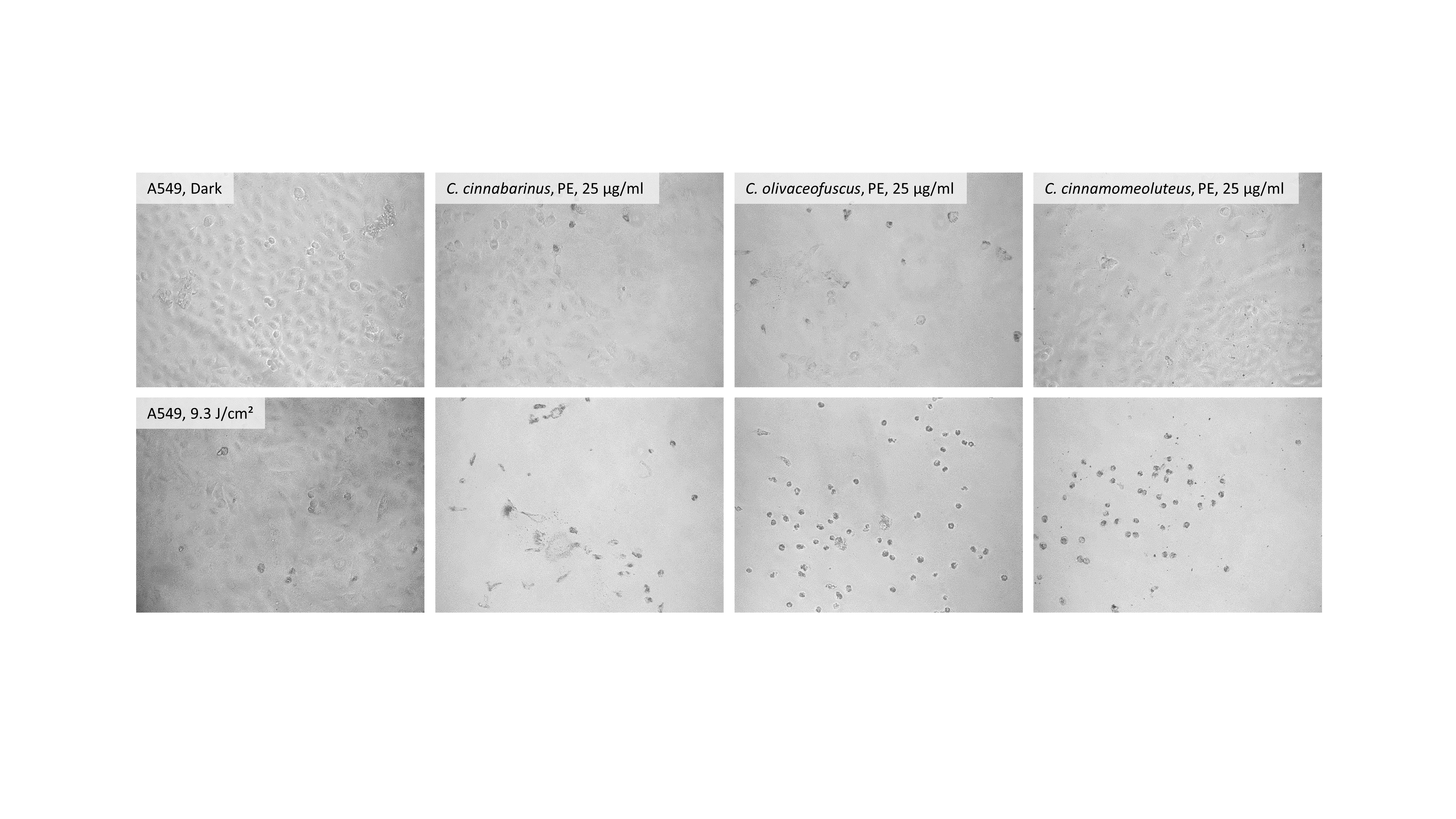


**Figure S44.** Micrographs (200x magnification) of cells of the lung cancer cell line A549 treated (24h) with the apolar extracts (25 µg/mL) of *C. cinnabarinus*, *C. olivaceofuscus*, and *C. cinnamomeoluteus.* The upper line of pictures shows treated cells in the dark, the lower after irradiation with blue light (468 nm, 9.3 J/cm²). The shrunken cells observed for cells with *C. cinnabarinus*, *C. olivaceofuscus*, and *C. cinnamomeoluteus* treated and irradiated indicate that a programmed cell death was induced while the reduced cell density e.g. *C. olivaceofuscus* under dark conditions implicates a cell proliferation inhibiting effect.


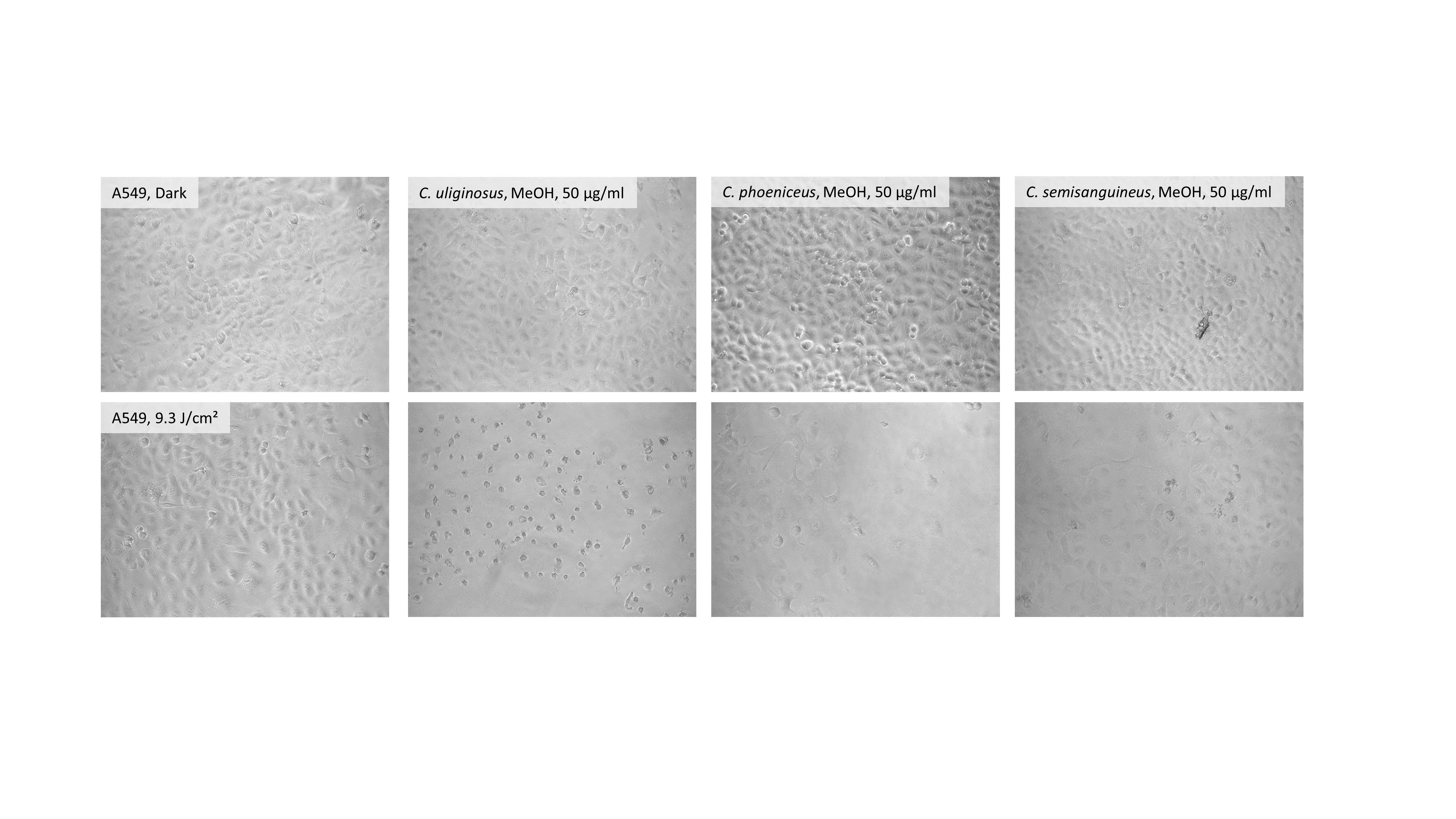


**Figure S45.** Micrographs (200x magnification) of cells of the lung cancer cell line A549 treated (24h) with the polar extracts (50 µg/mL) of *C. uliginosus*, *C. phoeniceus* and *C.semisanguineus.* The upper line of pictures shows treated cells in the dark, the lower after irradiation with blue light (468 nm, 9.3 J/cm²). The shrunken cells observed for cells treated with the polar extract of *C. uliginosus* and irradiated indicate that a programmed cell death was induced.


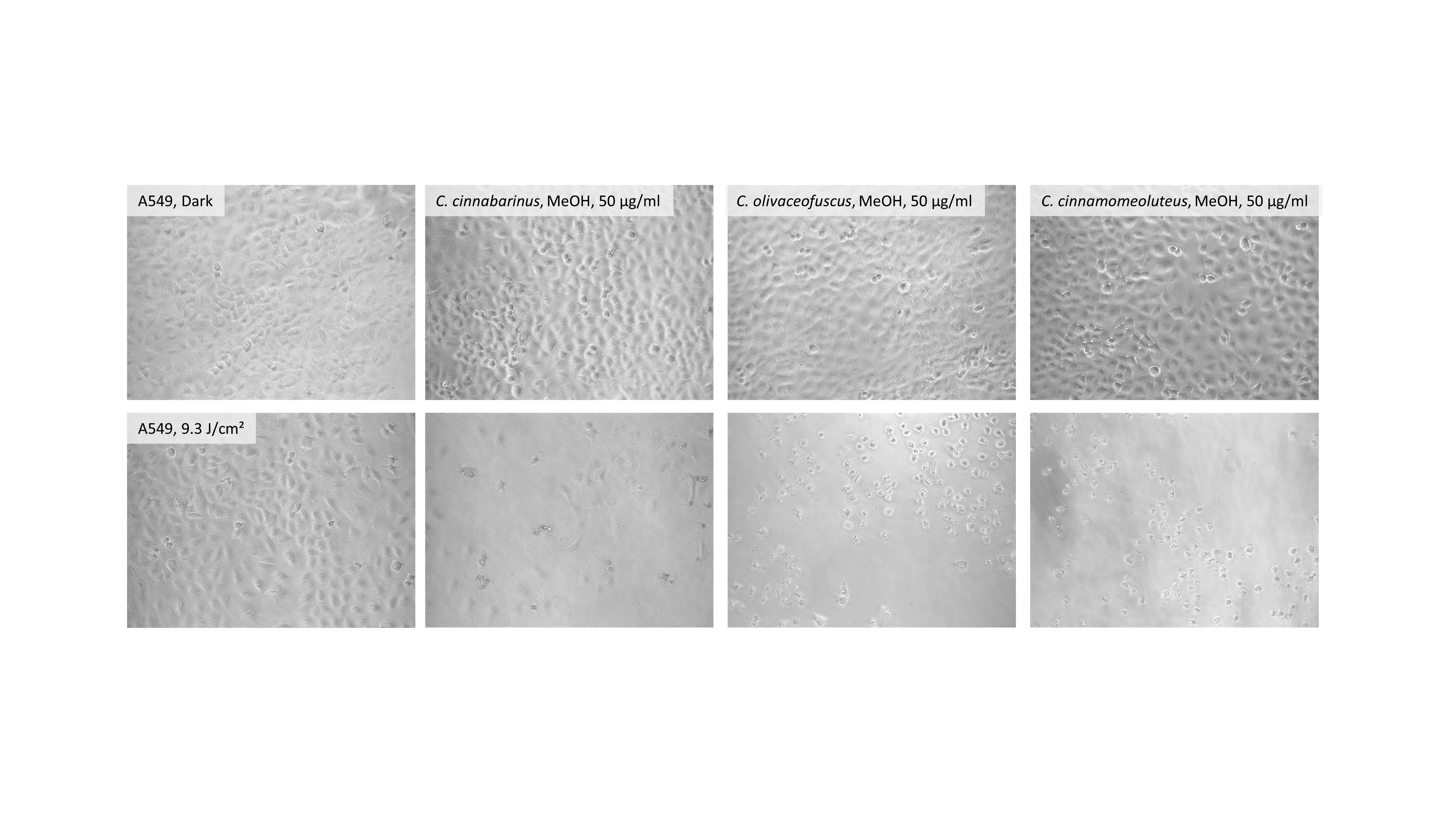


**Figure S46.** Micrographs (100x magnification) of cells of the lung cancer cell line A549 treated (24h) with the polar extracts (50 µg/mL) of *C. cinnabarinus*, *C. olivaceofuscus*, and *C. cinnamomeoluteus.* The upper line of pictures shows treated cells in the dark, the lower after irradiation with blue light (468 nm, 9.3 J/cm²). The shrunken cells observed for cells treated with the methanol extract of *C. olivaceofuscus*, and *C. cinnamomeoluteus* and irradiated indicate that a programmed cell death was induced.


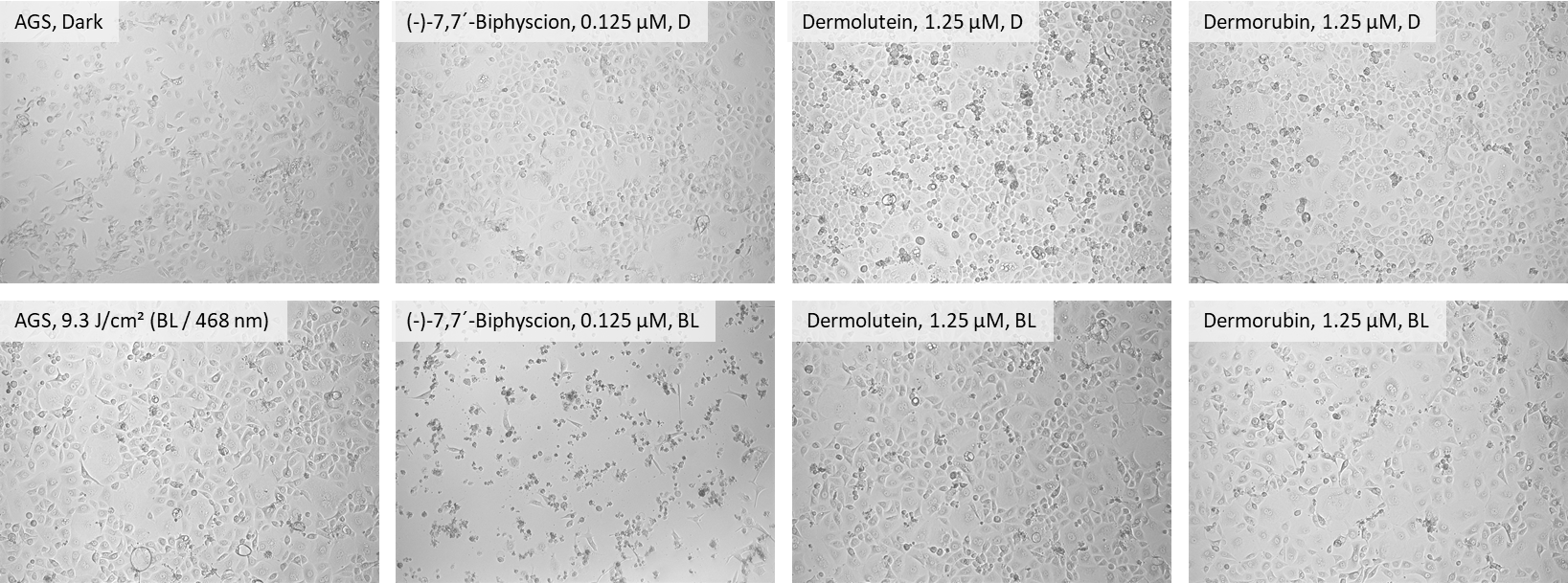


**Figure S47.** Micrographs of cells of the stomach cancer cell line (AGS / human Caucasian gastric adenocarcinoma, 100x magnification) treated (24h) with solutions of **1** (0.125 µM), **2** (1.25 µM), and **3** (1.25 µM). The upper line of pictures shows treated cells in the dark, the lower after irradiation with blue light (468 nm, 9.3 J/cm²). The shrunken cells observed for cells treated with a solution of **1** in combination with irradiation with blue light indicate that a programmed cell death was induced. Whereas no light-dependent apoptosis was detectable for the solutions of **2** or **3**.


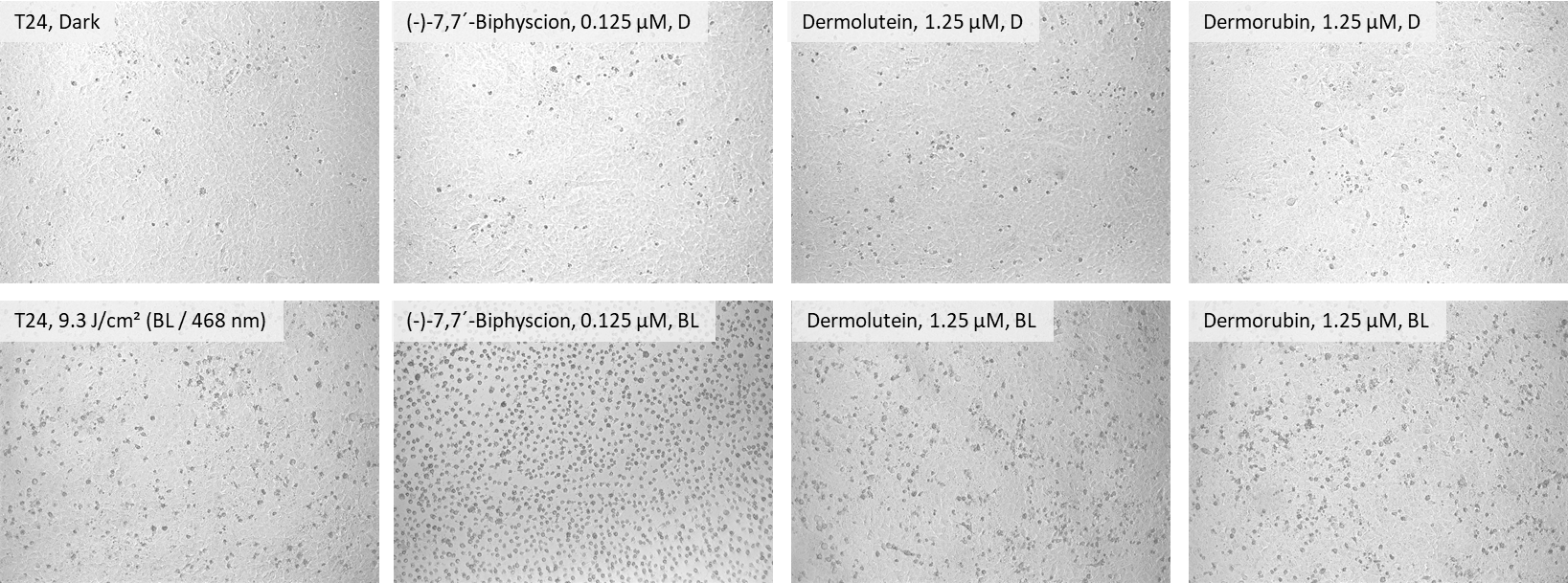


**Figure S48.** Micrographs of cells of the bladder cancer cell line (T24 / human bladder carcinoma, 100x magnification) treated (24h) with solutions of **1** (0.125 µM), **2** (1.25 µM), and **3** (1.25 µM). The upper line of pictures shows treated cells in the dark, the lower after irradiation with blue light (468 nm, 9.3 J/cm²). The shrunken cells observed for cells treated with a solution of **1** in combination with irradiation with blue light indicate that a programmed cell death was induced. Whereas no light-dependent apoptosis was detectable for the solutions of **2** or **3**.


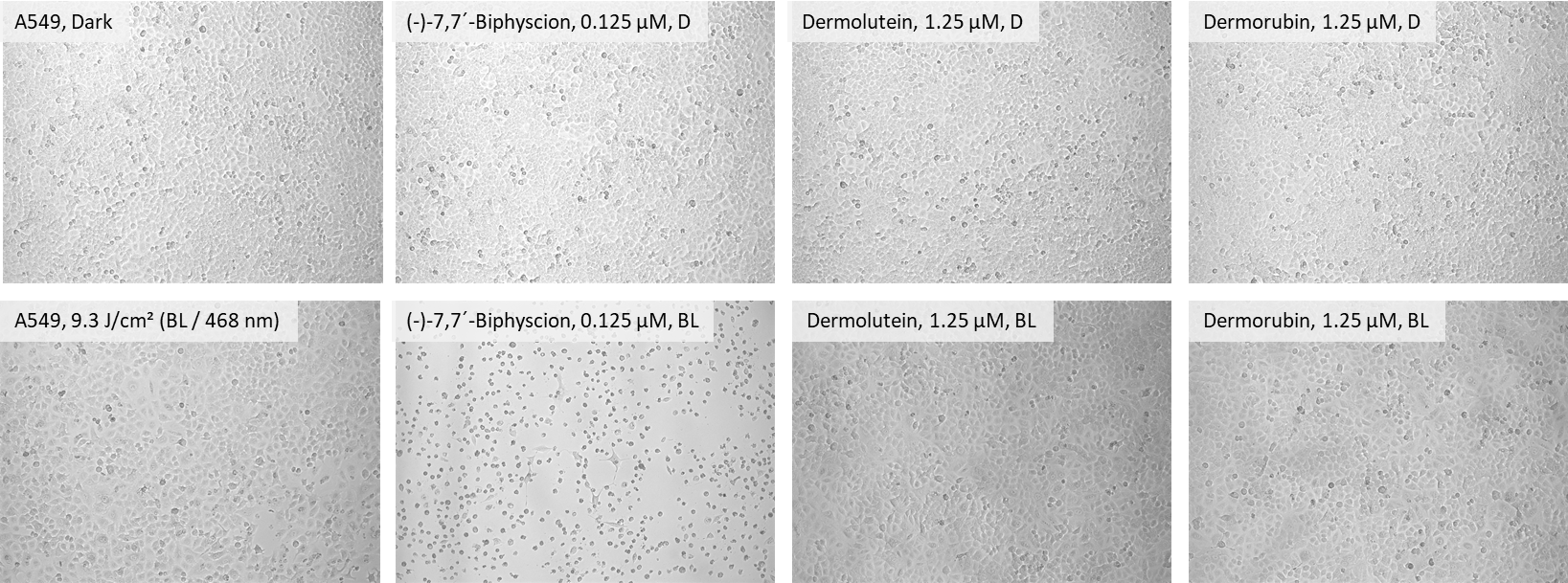


**Figure S49.** Micrographs of cells of the lung cancer cell line (A549 / human Caucasian lung carcinoma, 100x magnification) treated (24h) with solutions of **1** (0.125 µM), **2** (1.25 µM), and **3** (1.25 µM). The upper line of pictures shows treated cells in the dark, the lower after irradiation with blue light (468 nm, 9.3 J/cm²). The shrunken cells observed for cells treated with a solution of **1** in combination with irradiation with blue light indicate that a programmed cell death was induced. Whereas no light-dependent apoptosis was detectable for the solutions of **2** or **3**.

# References

1. S. L. Hopkins, B. Siewert, S. H. C. Askes, P. Veldhuizen, R. Zwier, M. Heger and S. Bonnet, *Photochemical & Photobiological Sciences*, 2016, **15**, 644-653.

2. G. Keller, *Sydowia*, 1982, **35**, 110-126.

3. B. Siewert, P. Vrabl, F. Hammerle, I. Bingger and H. Stuppner, *RSC Advances*, 2019, **9**, 4545-4552.

4. A. Gomes, E. Fernandes and J. L. F. C. Lima, *Journal of Biochemical and Biophysical Methods*, 2005, **65**, 45-80.

5. A. Rutz, M. Dounoue-Kubo, S. Ollivier, J. Bisson, M. Bagheri, T. Saesong, S. N. Ebrahimi, K. Ingkaninan, J.-L. Wolfender and P.-M. Allard, *Frontiers in Plant Science*, 2019, **10**.

6. M. C. Chambers, B. Maclean, R. Burke, D. Amodei, D. L. Ruderman, S. Neumann, L. Gatto, B. Fischer, B. Pratt, J. Egertson, K. Hoff, D. Kessner, N. Tasman, N. Shulman, B. Frewen, T. A. Baker, M.-Y. Brusniak, C. Paulse, D. Creasy, L. Flashner, K. Kani, C. Moulding, S. L. Seymour, L. M. Nuwaysir, B. Lefebvre, F. Kuhlmann, J. Roark, P. Rainer, S. Detlev, T. Hemenway, A. Huhmer, J. Langridge, B. Connolly, T. Chadick, K. Holly, J. Eckels, E. W. Deutsch, R. L. Moritz, J. E. Katz, D. B. Agus, M. MacCoss, D. L. Tabb and P. Mallick, *Nature Biotechnology*, 2012, **30**, 918-920.

7. T. Pluskal, S. Castillo, A. Villar-Briones and M. Orešič, *BMC Bioinformatics*, 2010, **11**, 395.

8. K. Dührkop, M. Fleischauer, M. Ludwig, A. A. Aksenov, A. V. Melnik, M. Meusel, P. C. Dorrestein, J. Rousu and S. Böcker, *Nature Methods*, 2019, **16**, 299-302.

9. K. Dührkop, H. Shen, M. Meusel, J. Rousu and S. Böcker, *Proceedings of the National Academy of Sciences*, 2015, **112**, 12580-12585.

10. A. Gaudry, L. Quirós, A. Rutz, M. Dounoue, M. Kaiser, B. David, L. Marcourt, E. F. Queiroz, J. L. Wolfender and P. M. Allard, *Planta Med*, 2019, **85**, P-109.

11. Z. An, *Handbook of Industrial Mycology*, CRC Press, 2004.

12. X.-Q. Zhou, A. Busemann, M. S. Meijer, M. A. Siegler and S. Bonnet, *Chemical Communications*, 2019, **55**, 4695-4698.

13. M. C. DeRosa and R. J. Crutchley, *Coordination Chemistry Reviews*, 2002, **233-234**, 351-371.

14. D. Garcìa-Fresnadillo, Y. Georgiadou, G. Orellana, A. M. Braun and E. Oliveros, *Helvetica Chimica Acta*, 1996, **79**, 1222-1238.

15. R. Schmidt, C. Tanielian, R. Dunsbach and C. Wolff, *Journal of Photochemistry and Photobiology A: Chemistry*, 1994, **79**, 11-17.

16. A. Bahreman, B. Limburg, M. A. Siegler, E. Bouwman and S. Bonnet, *Inorganic Chemistry*, 2013, **52**, 9456-9469.

17. B. Page, M. Page and C. Noel, *Int J Oncol*, 1993, **3**, 473-476.

18. B. Siewert, J. Wiemann, A. Koewitsch and R. Csuk, *Eur. J. Med. Chem.*, 2014, **72**, 84-101.

19. P. N. Dean and J. H. Jett *Journal of Cell Biology*, 1974, **60**, 523-527.

20. A. R. Wielgus, B. Zhao, C. F. Chignell, D.-N. Hu and J. E. Roberts, *Toxicology and Applied Pharmacology*, 2010, **242**, 79-90.

21. K. Fatima, N. Masood and S. Luqman, *Biomedical Research and Therapy*, 2016, **3**.

22. A. Casas, C. Perotti, H. Fukuda and A. Batlle, *Proc. SPIE-Int. Soc. Opt. Eng.*, 2001, **4248**, 179-188.

23. A. Bozkir, B. Simsek, A. Gungort and M. Torun, *J Clin Pharm Ther*, 1999, **24**, 43-47.

24. W. Baumler, C. Abels, S. Karrer, T. Weiss, H. Messmann, M. Landthaler and R. M. Szeimies, *Br. J. Cancer*, 1999, **80**, 360-363.
